# PubMedPortable: A Framework for Supporting the Development of Text Mining Applications

**DOI:** 10.1371/journal.pone.0163794

**Published:** 2016-10-05

**Authors:** Kersten Döring, Björn A. Grüning, Kiran K. Telukunta, Philippe Thomas, Stefan Günther

**Affiliations:** 1 Pharmaceutical Bioinformatics, Institute of Pharmaceutical Sciences, Albert-Ludwigs University, 79104 Freiburg, Germany; 2 Bioinformatics, Institute of Computer Science, Albert-Ludwigs University, 79110 Freiburg, Germany; 3 Language Technology Lab, German Research Center for Artificial Intelligence, DFKI GmbH, 10559 Berlin, Germany; University of Colorado, UNITED STATES

## Abstract

Information extraction from biomedical literature is continuously growing in scope and importance. Many tools exist that perform named entity recognition, e.g. of proteins, chemical compounds, and diseases. Furthermore, several approaches deal with the extraction of relations between identified entities. The BioCreative community supports these developments with yearly open challenges, which led to a standardised XML text annotation format called BioC. PubMed provides access to the largest open biomedical literature repository, but there is no unified way of connecting its data to natural language processing tools. Therefore, an appropriate data environment is needed as a basis to combine different software solutions and to develop customised text mining applications. PubMedPortable builds a relational database and a full text index on PubMed citations. It can be applied either to the complete PubMed data set or an arbitrary subset of downloaded PubMed XML files. The software provides the infrastructure to combine stand-alone applications by exporting different data formats, e.g. BioC. The presented workflows show how to use PubMedPortable to retrieve, store, and analyse a disease-specific data set. The provided use cases are well documented in the PubMedPortable wiki. The open-source software library is small, easy to use, and scalable to the user’s system requirements. It is freely available for Linux on the web at https://github.com/KerstenDoering/PubMedPortable and for other operating systems as a virtual container. The approach was tested extensively and applied successfully in several projects.

## Introduction

Large progress has been made in the field of text mining and natural language processing (NLP) in the biomedical domain [1]. This includes identification of protein-protein interactions [2], drug-drug interactions [3], compound-protein interactions [4, 5], and their connection to diseases [6]. Nevertheless, research efforts are still hindered by a lack of standardised ways to process the vast amount of data. This matter can be divided into two issues. First, there is the problem of interoperability between NLP components for named entity recognition (NER) and relation extraction methods. Second, literature-related data needs to be accessible for large-scale applications.

In this publication, the problem of data accessibility is approached with a combination of relational database and full text index. While a full text index can be built for complex Boolean text queries, a relational database is suitable for storing all meta information of PubMed articles and collecting statistics.

The issue of connecting different software solutions for natural language processing is covered by the implementation of an interface to the BioC interchange format. The aim of PubMedPortable is to enable users to develop text mining applications and use cases with very basic programming knowledge. This is understood in terms of a stand-alone application without dependency on web services, but with the possibility to query them if desired. Any tool in a customised workflow supporting BioC input and output modules can be applied to perform NLP tasks independently, but there are also other directly usable data formats, as shown in the remainder of this article.

### Related work on Software Interoperability

Several proposals have been made to tackle this research area, but only a few of them have obtained wide interest in the community, namely the Unstructured Information Management Architecture (UIMA) [7, 8], the General Architecture for Text Engineering (GATE) [9], and the BioC XML data format [10]. UIMA defines Text Analysis Engines (TAEs), the text processing software modules, and a common analysis structure (CAS), the XML-based input and output format for TAEs [10]. U-Compare is a Java Web Start application that offers drag-and-drop construction of workflows for UIMA-compatible NLP tools [11]. The GATE Developer is an integrated development environment in Java similar to U-Compare [7]. GATE provides an interface to UIMA as well. A large-scale example for the application of UIMA is the AusTalk corpus [12], a 3,000 hour auditory-visual corpus of Australian English. Its data sets as well as well as other corpora are accessible via the Alveo Virtual Laboratory [13], a web platform consisting of tools in different programming languages, ensuring interoperability with UIMA. As an alternative to the complex architectures of UIMA and GATE, docrep [14] represents a lightweight document representation framework for serialisation of textual data with linguistic annotations.

BioC uses a minimalistic approach, as only the data format of XML files is defined in a document type definition (DTD) file, accompanied by the user-specific semantics of data and annotations, which are described in an extra key file. The interoperability is ensured within the BioC workflow, defining an Input Connector to read and an Output Connector to write BioC XML data. The interface to these BioC classes is implemented in several programming languages [10, 15].

Rak *et al*. claimed that there is a tendency towards workflow construction platforms, but that their software dependencies on a source platform can restrict the development process [7]. Therefore, web services became popular to solve NLP tasks, especially because of the Representational State Transfer (REST) architecture. This was their motivation to develop Argo, an UIMA-based online text mining workbench, supporting different data formats like CAS and BioC. Argo offers a range of web service components to be used in a workflow, without additional programming efforts [7]. There are advantages and disadvantages for choosing UIMA or BioC, but the integration of UIMA-compatible modules can be considered as a more complex process than generating a BioC-compatiple XML document [15]. Therefore, PubMedPortable implements a BioC interface. End users can easily apply and combine BioC NLP tools to arbitrary PubMed data sets, whereas developers can focus on sophisticated pipelines, including e.g. machine learning approaches.

### Approaches to Process PubMed Data

At the beginning of 2016, PubMed consisted of more than 25 million records and the number increases quickly. Considering the issue of how to deal with this large amount of data and to apply NLP methods effectively, quite a few published as well as unpublished approaches exist.

There are efforts to simplify literature searches in PubMed and to support text annotation. Two of the most recent web service developments are OntoGene [6] and PubTator [16], which provide a BioC interface.

Working with PubMed publications on a local machine is possible, too. The complete data set of PubMed can be downloaded as XML files from the NLM FTP server, including example data sets. The user can also apply the NCBI interface to download a set of PubMed XML files related to a specific search term [17]. Biopython can be used to connect to this interface, named EFetch. It also contains a library to parse PubMed XML files [18]. The content of XML files does not have to be processed multiple times if the text elements are stored in a database. The LingPipe project is implemented in Java and offers a library to parse PubMed XML files and build a full text index with Lucene [19]. It contains a short tutorial about loading the abstract and title texts into a MySQL relational database in a version from 2010. It is worth noting that the PubMed XML schema is updated annually. Biopython and LingPipe are considered sophisticated tools, but this also means that it is a complex task to modify existing and develop new functions to process and index all recently available PubMed XML attributes.

There are finished implementations building a relational database from PubMed XML files. According to the tendency towards web services as mentioned by Rak *et al*. [7], these approaches were already published around 10 years ago [20, 21]. Yoo *et al*. describe a complex system for downloading PubMed and PMC articles in XML format, storing them in a MySQL relational database, and searching the documents with a Lucene full text index [21]. Unfortunately, their service is not hosted anymore. In 2004, Oliver *et al*. compared different approaches to loading PubMed XML files into a relational database [20]. This included an Oracle 9i and an IBM DB2 relational database, combined with Java and PERL code. There is an unpublished update version from 2010 using Java 6 and MySQL 5.1 [22].

In PubMedPortable, this SQL schema is completely adapted and slightly modified, but combined with object-relational mapping (ORM) in Python to generate a PostgreSQL database from PubMed XML files. That means, changes of SQL tables or columns can be introduced directly in the parser itself, and the whole uploading process can be upscaled to the number of desired CPU cores. PubMedPortable implements a Xapian full text index similar to Yoo *et al*. [21], as any PostgreSQL column can be indexed with this technology with only a few lines of code. Currently, abstract titles and texts, MeSH terms, keywords, and chemical substances are indexed in the standard implementation. There is also a modified version only indexing abstract titles and texts. Using a Xapian index offers fast and straight forward keyword and context search, as shown by the use cases described in the results section.

While PubMedPortable was tested in Ubuntu and Fedora, there is also a one-click-solution based on Docker [23], so that the relational database and the full text index can be used in more operating systems. Docker is similar to a virtual machine and easy to deploy. Therefore, PubMedPortable helps to standardise the way of processing large literature data sets and making them accessible for text mining applications.

## Methods

### Data Accessibility

**Fig 1:**
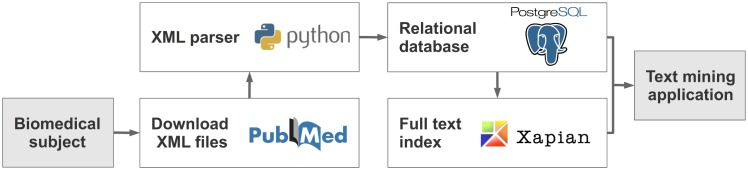
PubMedPortable workflow. 1) Download XML files from PubMed. 2) Parse and upload data into a PostgreSQL relational database. 3) Build a Xapian full text index. 4) Develop text mining applications.

The basic requirements are an installation of Python, Xapian, and PostgreSQL, as well as around 300 GB free disk space in case of processing all PubMed XML files. Fig 1 shows the general workflow for loading PubMed XML files into a relational database and generating a full text index.

PubMedPortable is usable via a command-line interface and requires PubMed XML files as input. A database needs to be created and configured at first. The tables are built based on user-provided data. The SQL schema can be seen in the GitHub project folder. All PubMed XML attributes are transformed into PostgreSQL tables and columns with an ORM approach using a SAX parser. After processing the PubMed XML files, a full text index is built by querying titles, abstracts, MeSH terms, keywords, and chemical substances from the relational database. These installation steps can also be executed at once using the virtual container Docker without installing additional software packages.

Based on this data environment, phrase and Boolean searches can be executed with user-defined terms. It is possible to generate charts and statistics by combining the full text search with SQL queries. A range of examples and detailed installation instructions are precisely described on the GitHub project page.

### BioC Interface

A multitude of BioC compatible tools can be applied by exporting articles from PubMedPortable to BioC format. This includes the NER tools from Wei *et al*., identifying chemicals/species, diseases, mutations/variations, and genes/proteins [24], included in the PubTator web service [16]. Tokenisation, part-of-speech (POS) tagging, and sentence parsing can be e.g. performed with the BioC NLP pipeline from Comeau *et al*. [25].

**Fig 2:**
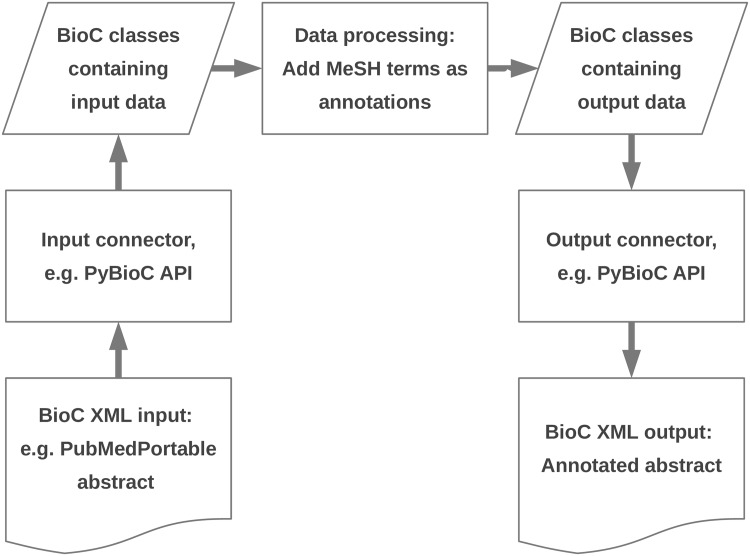
General BioC workflow. This is the minimalistic approach from Comeau *et al*. [10] with the example how to add MeSH terms to BioC PubMed titles and abstracts from the PubMedPortable PostgreSQL database.

The BioC workflow as shown in Fig 2 implies that any tool supporting this input and output format can add annotations to a document, supporting the idea of interoperability. The PubMedPortable documentation describes how MeSH terms from the relational database can be added to a BioC XML document.

**Fig 3:**
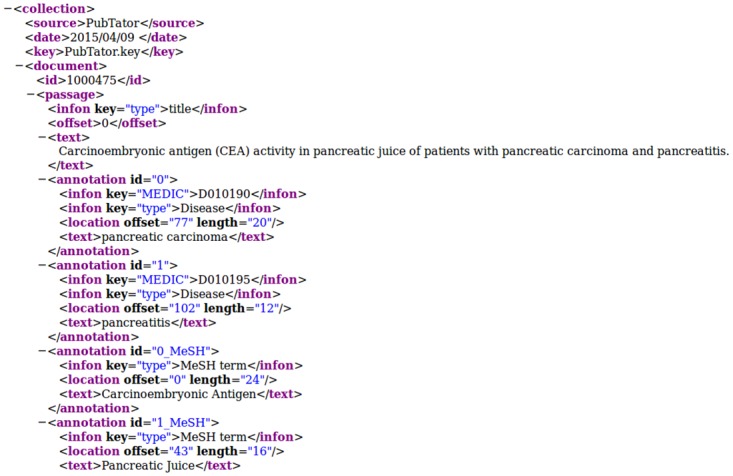
Excerpt of a BioC XML document. The document ID 100475 is a PubMed ID. PubTator annotations are shown with infon elements that contain the key *type* with the value *Disease* and the key *MEDIC* referring to a MeSH ID, such as *D010190* for the given disease *pancreatic carcinoma*. The PubMedPortable MeSH term annotations are shown with the annotation IDs *0_MeSH* and *1_MeSH* to make them distinguishable from the normally iterating PubTator annotation IDs. They were added after calling the PubTator web service.

Fig 3 shows the output of invoking PubTator and merging the results with MeSH term annotations as a BioC document. All implementation details can be found in the GitHub project folder.

As mentioned in the introduction, the requirements for BioC are a DTD file to define the XML document structure and a key file to explain which structural elements are used in a particular BioC XML document. XML infon elements are used to differentiate between distinct XML elements. Every infon element contains a key and a value. This basic element can refer to the document sections title or text, to token IDs, or to NER annotations. Every BioC XML file starts with naming the original creation source and the creation date. Within the XML schema of Fig 3, the text collection consists of PubMed articles with their PubMed ID as document ID. Each document consists of a title and the abstract text, if given. In this case, the file PubTator. key describes the semantics used by this web service. For the sake of clarity, Comeau *et al*. intend that only one type of annotation should be used within one BioC XML file consisting of several documents [10], but they can also be combined as illustrated here.

**Fig 4:**
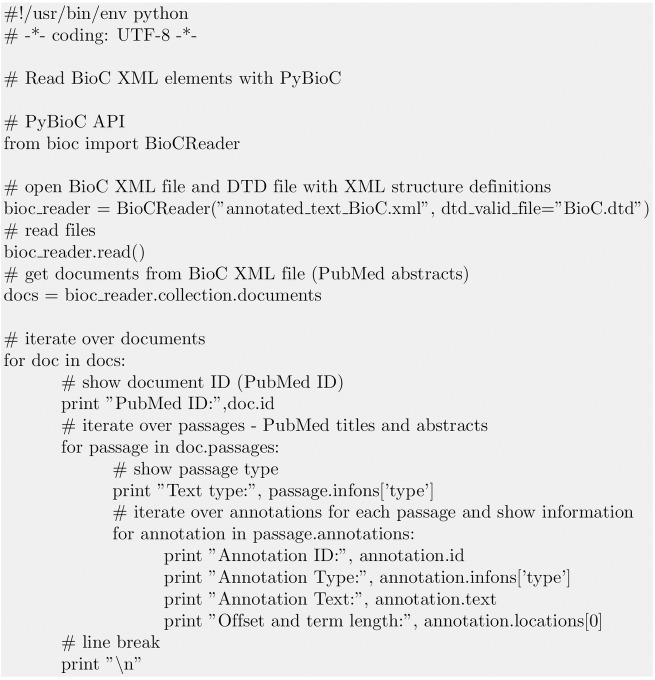
Read BioC elements. All BioC XML elements can be read with the BioC API. The script refers to the left part of the workflow shown in Fig 2. Iterating over the given annotations as shown in Fig 3 will e.g. show *Annotation ID: 0*, *Annotation Type: Disease*, *Annotation Text: pancreatic carcinoma*, and *Offset and term length: 77:20*.

Fig 4 shows a code snippet how to read in and iterate over BioC XML elements with their annotations.

## Results and Discussion

### Performance

For the complete XML data set available in 2015 with a size of 114 GB, it took 10.5 days to build the PostgreSQL relational database and another 27 hours to generate the full text index using a 2.8 GHz quad-core processor. The time of the indexing process and the size of the index depends on the number of extracted fields. A modification of the PubMedPortable scripts, including only abstract titles and texts, but not MeSH terms, keywords, and substances, led to a runtime of 10 hours. The size of the full text index also decreased from 154 GB to 124 GB. It is difficult to compare the runtime to the results from Oliver *et al*. [20] due to different hardware and software system requirements, but increasing computational resources will speed up this process in general. Using 48 CPU cores with 2.1 GHz reduced the result calculation time of 10.5 days to 20 hours.

### Pancreatic Cancer Data Set

The PubMedPortable documentation refers to a small data set of 23,258 PubMed IDs (272 MB) related to pancreatic cancer, which is processed in a few minutes. The documents were selected by performing a phrase search with the term pancreatic cancer using the NCBI web service. Pancreatic cancer is one of the most dangerous cancer types. Currently, the only way to cure a patient is surgery, beside several therapeutic strategies that cannot significantly increase survival rates [26]. The research progress in this area can be supported with text mining methods, e.g. by covering findings about gene-disease and compound-protein relationships.

### Use Case: BioC Applications

To get a first impression of important genes or proteins, drugs, and diseases related to pancreatic cancer, these entities were automatically identified in the approximately 23,000 abstracts. Gene and protein synonyms were extracted with GeneTUKit [27]. Disease terms were annotated with the DNorm [28]. Both tools were used without retraining their models on an annotated corpus. Chemical compounds were identified with tmChem [29] via the PubTator web service [16]. DNorm and tmChem as stand-alone applications work with BioC as well as with an individual tab-separated format. GeneTUKit takes texts in a pseudo XML format as input and generates tab-separated annotations as output. The software was embedded into a short pipeline to be used with PubMedPortable, described in a GitHub side project [30].

**Fig 5:**
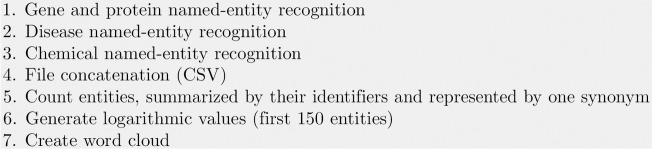
Documentation to generate a word cloud using PubMedPortable. Different tools and different data formats might be used for named-entity recognition. Tab-separated files (CSV) with PubMed ID, synonym, and identifier in each line are used to collect all abstracts in which a match for identifier-specific synonyms appeared.

**Fig 6:**
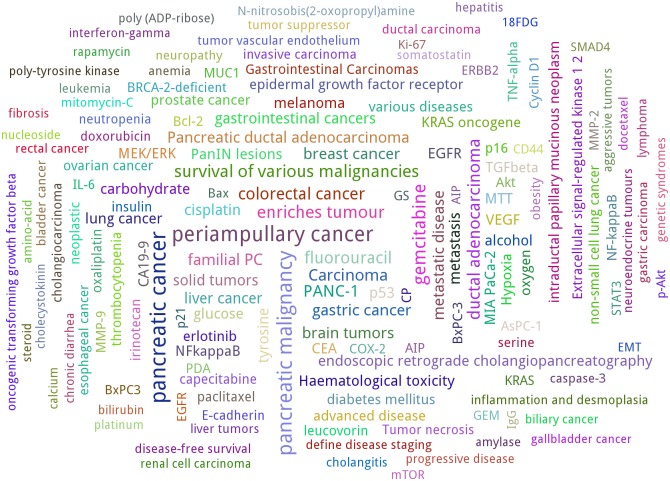
Genes, proteins, chemicals, and diseases related to pancreatic cancer. The 150 most frequently appearing entities in terms of their number of abstracts were identified with DNorm [28], GeneTUKit [27], and PubTator [16]. Fig 5 shows the steps to generate this word cloud.

The number of abstracts for each entity was counted based on summarising the synonyms for each identifier provided, e.g. Entrez-GeneID numbers. One identifier probably refers to different synonyms, and some annotated examples did not receive such a code. From all extracted entities, the 150 most commonly used identifiers were extracted to be illustrated in a word cloud. Fig 5 shows the steps to create the word cloud shown on Fig 6. All details can be found in the project wiki on GitHub.

One approach to evaluate the main entities shown in Fig 6 is to compare diseases and gene or protein synonyms with information provided by the database OMIM [31]. Chemicals identified as drugs can be reviewed in DrugBank [32]. These databases are focused on human diseases, their interrelated gene mutations, and how to treat them. One assumption is that if e.g. specific oncogenes like *KRAS* are presented in OMIM, the NER search should result in a reasonable number of publications. The relative frequencies of search terms are displayed in the word cloud in Fig 6 by different sizes. The absolute numbers of the 15 most frequently appearing diseases, genes or proteins, and chemicals are summarized in Table 1. The majority of terms is clearly related to cancer or inflammation processes. *KRAS* is part of the largest displayed genes. The protein *p16* refers to the gene *CDKN2A*. *PANC-1* and *MIA PaCa-2* refer to cell lines. Another point to mention about the word cloud is that Entrez-GeneID numbers are organism-specific. Therefore, the large *EGFR* example refers to the human gene (GeneID 37455) and the slightly smaller term *epidermal growth factor receptor* to *Drosophila melanogaster* (GeneID 1956). However, the process of gene normalisation needs to be treated cautiously in the case of short texts as PubMed titles and abstracts. Gemcitabine (DrugBank ID DB00441) is the most frequently occurring chemical, a nucleoside analog commonly used in chemotherapy [26]. Periampullary cancer is located close to the ampulla of Vater (pancreatic duct), possibly related to jaundice [33]. Pancreatic ductal adenocarcinoma is the most frequently appearing type of pancreatic cancer and the most common lethal cancer [34]. Fig 6 and Table 1 illustrate that texts referring to pancreatic cancer are also related to colorectal, breast, gastric, and liver cancer, as well as diabetes, and other disease-associated terms. The importance or relevance of a relation between pancreatic cancer and a selected entity can be inferred by considering the number of co-occurrences or analysed with more sophisticated methods, e.g. the approach of kernel methods as applied for the identification of protein-protein interactions [2] and compound-protein interactions [35].

**Table 1:** 15 most commonly found entities from the 3 entity types disease, gene/protein, and chemical with the number of identified PubMed abstracts.

Rank	Disease	#	Gene/protein	#	Chemical	#
1	pancreatic cancer	22,823	*PANC-1*	884	gemcitabine	2,806
2	periampullary cancer	13,302	*p53*	409	fluorouracil	1,055
3	pancreatic malignancy	9,364	*VEGF*	380	cisplatin	442
4	ductal adenocarcinoma	1,544	*EGFR*	361	tyrosine	420
5	survival of various malignancies	1,279	*MIA PaCa-2*	307	carbohydrate	383
6	enriches tumour	1,260	*CA19-9*	294	alcohol	381
7	colorectal cancer	1,082	*NFkappaB*	277	glucose	373
8	Carcinoma	818	*CEA*	273	MTT	354
9	breast cancer	747	*MEK/ERK*	272	erlotinib	311
10	gastric cancer	677	*epidermal growth factor receptor*	266	oxygen	263
11	familial PC	647	*TGFbeta*	259	oxaliplatin	227
12	metastatic disease	602	*KRAS oncogene*	258	insulin	223
13	brain tumors	506	*NF-kappaB*	237	capecitabine	217
14	Pancreatic ductal adenocarcinoma	506	*p16*	229	leucovorin	214
15	liver cancer	444	*Bcl-2*	229	paclitaxel	208

This use case illustrates how the BioC interoperability can be applied for fast development of prototypic text mining applications with PubMedPortable in terms of software modularity. More BioC-related software and text corpora can be found in the overview of the BioCreative IV interoperability track [36] and within the BioC track of the BioCreative V challenge [37]. Wei *et al*. provide an overview of NER applications published within the last ten years [24].

### Use Case: Querying PubMedPortable Data Sets Related to Pancreatic Cancer

**Fig 7:**
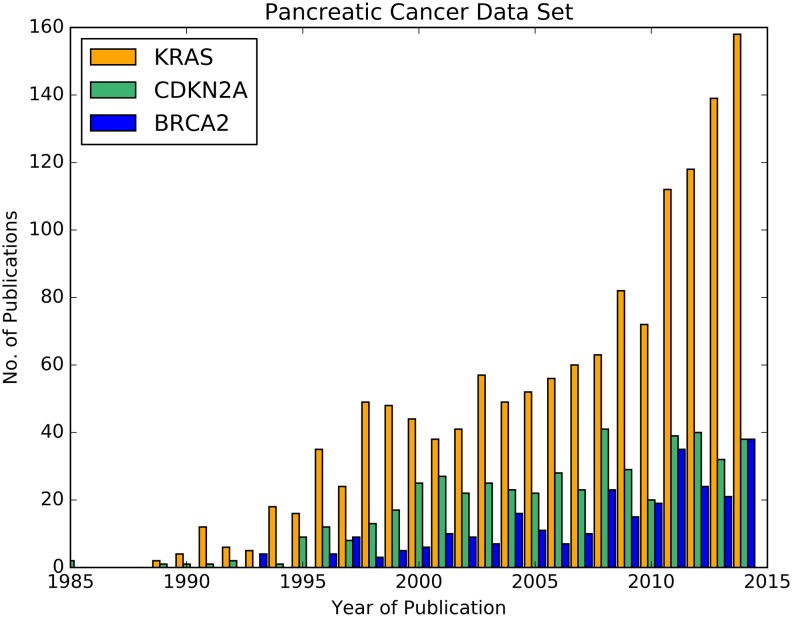
Timelines for the publications of the genes *KRAS*, *BRCA2*, and *CDKN2A* until 2014. The PubMed IDs for these three genes were extracted from the list of entities resulting from step 4 in Fig 5. The publication years were selected from the PubMedPortable database.

According to the OMIM review about pancreatic cancer mentioned in the last subsection, three substantially involved genes are *KRAS*, *CDKN2A*, and *BRCA2*. While Fig 6 shows their relative frequencies in comparison to other search terms, the timelines in Fig 7 illustrate the absolute number of publications per year. In contrast to Fig 6, different organism-specific Entrez GeneID numbers were summarized. Compared to the other two genes, the *KRAS* timeline grows strongest. There is actually no slope for the *CDKN2A* timeline, but the amounts are consistently on a higher level than the ones of *BRCA2*, although converging in 2010. Both genes show a rather low number of publications compared to *KRAS*. One reason for this outcome is the role of *KRAS* in the regulation of cell proliferation and its higher specificity to pancreatic cancer than in case of *BRCA2* and *CDKN2A* [38]. In combination with the word cloud, these examples present ideas to visually inspect the number of publications of specific entities.

**Fig 8:**
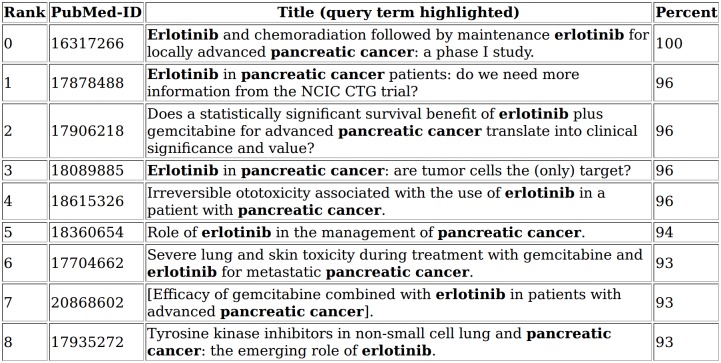
Boolean query result. The HTML page shows a rank in the first column with a relative match score, scaled to 100. The NEAR condition was used to allow up to four other words between the drug erlotinib and the disease term pancreatic cancer without fixed word order.

One way to investigate the context of selected search terms is to use the PubMedPortable function to generate an HTML page with highlighted entities. The full text index can be searched with keyword queries for this purpose. The PubMedPortable documentation describes examples, such as a query for the drug erlotinib (DrugBank ID DB00530) next to the term pancreatic cancer, having a maximum of four words between them (Fig 8). Although this example appears to be slightly artificial, it represents a number of use cases. Boolean queries can increase the precision of the search results, focusing on the word neighbourhood and excluding closely related findings. The usage of such HTML pages with highlighted search terms demonstrates a basic way to simplify text curation and annotation. Search results can be ranked with a Xapian-specific score. This illustrates how a full text index can be easily used to display search results in a user-friendly view.

Oliver *et al*. showed a rather complex SQL query, selecting the ten journals that published the most articles with the MeSH term “Leukemia” [20]. Analogously, the slightly modified query to the PubMedPortable database is shown in Table 2. The result is displayed in Table 3, with the outcome that the order of top range journals did not change a lot within the last ten years. In contrast to the other use cases described here, this selection was applied to the complete PubMed data set.

**Table 2:** PostgreSQL query to select the ten journals with the highest number of publications containing the MeSH term “Leukemia” [20] on the complete PubMed data set.

SQL command	
SELECT	mj.medline_ta, count(mj.fk_pmid) as num_of_publications
FROM	pubmed.tbl_medline_journal_info mj
JOIN	pubmed.tbl_mesh_heading msh
ON	mj.fk_pmid = msh.fk_pmid
WHERE	msh.descriptor_name = ‘Leukemia’
GROUP BY	mj.medline_ta
ORDER BY	count(mj.fk_pmid) desc
FETCH	first 10 rows only;

**Table 3:** Results for the query shown in Table 2.

Journal	Number of publications
Blood	1,469
Cancer	748
Leukemia	746
Leuk Res	723
Cancer Res	718
Bone Marrow Transplant	710
Br J Haematol	677
Rinsho Ketsueki	671
Lancet	582
Haematologica	486

The PubMedPortable documentation on GitHub contains other detailed ideas how to apply the software library, which are not shown here. One example is to process all abstracts and titles which were found with the drug name gemcitabine. The most frequently occurring terms are shown in an additional word cloud to reveal part of the vocabulary related to pancreatic cancer. A whitespace tokeniser was used that separates words as tokens and removes punctuation. Another example combines a Boolean search in Xapian for selected terms with PostgreSQL queries to identify the main authors by their number of publications. Subsequently, their investigated topics can be compared to the entities in the word clouds. Similar to the query in Table 2, it can be investigated in which countries the most journals in reference to the pancreatic cancer data set are located.

All examples shown here illustrate ideas how PubMedPortable can be applied to user-specific data sets. It depends on the user’s aim, what kind of data analysis will be included in a text mining application.

### Extending PubMedPortable

The selection of Python, PostgreSQL, and Xapian is based on the requirement to provide a framework that is easy to install, independent from a web service, and usable with only a few lines of code. Using another type of database management system such as Oracle or MySQL with the ORM approach is possible, as well as using the Xapian interfaces for Java or C++ instead of Python. PubMedPortable enables the user to directly create individual queries and to extend this framework with sophisticated NLP approaches. The PubMedPortable database can be used as a centralised repository, which is regularly updated. This also offers the possibility to monitor changes in a data set over time by executing a workflow repeatedly. Furthermore, PubMedPortable supports indexing of unformatted PubMed Central (PMC) full texts. The combination of a relational database and a full text index can also be integrated in a web server, as shown in previous publications [4, 39]. In these projects, the PubMedPortable framework was extended with named entity recognition for gene or protein names from UniProt and PubChem synonyms for small molecules.

Beside the illustrated use case of accessing the MeSH terms in the PubMedPortable relational database, the users might wish to further classify their texts with ontology concepts using the web service of the Open Biomedical Annotator (OBA) [40], e.g. with diseases.

Texts in a corpus can also be categorised using topic models with the Latent Dirichlet allocation as invented by Blei *et al*. [41] and implemented in Python on GitHub by V. Sandulescu.

Another approach is to use tools implemented in a workflow management system like Galaxy [12, 42], supporting drag-and-drop construction of NLP pipelines as applied in the already mentioned Alveo platform [13].

To conclude, the BioC interface provides manifold applications for NLP, but there is also a range of approaches to extract information using other sources as described for some detailed examples. This emphasises that PubMedPortable can be used in a modular way depending on the user’s needs.

## Conclusion

PubMedPortable offers a ready-to-start solution for developing large-scale text mining applications by generating an in-house database from PubMed articles. The resulting data environment supports complex relational database queries and fast full text search. The BioC interface and the possibility to use Docker provide interoperability to apply NLP approaches in different programming languages and to run queries on several operating systems without much programming effort. PubMedPortable combines and updates selected approaches from related work to result in a state-of-the-art software library as described by the presented use cases. All software and included methods are open-source and free to be modified for further refinements and improvements within the community.

## Supporting Information

S1 File This GitHub file contains the complete PubMedPortable documentation and the source code with all examples and results presented in this article. (ZIP)
