# Supplementary material for: PubMedPortable: A Framework for Supporting the Development of Text Mining Applications
Source: PLoS One. 2016 Oct 5;11(10):e0163794. doi: 10.1371/journal.pone.0163794 (PMC5051953; doi:10.1371/journal.pone.0163794)
Supplement: S1 File — (ZIP) [file pone.0163794.s001.zip › PubMedPortable-master/documentation/PostgreSQL_database_schema.html]

xml version='1.0' encoding='UTF-8' standalone='yes'?


pubmed


 
 

 
 


1


Project PostgreSQL @ DbSchema.com
Layout pubmed
Column comments as mouse-over tooltips.


Fk fk\_abstract
tbl\_abstract ref tbl\_medline\_citation ( fk\_pmid -> pmid )

fk\_pmid


Fk fk\_accession\_number\_list
tbl\_accession\_number ref tbl\_medline\_citation ( fk\_pmid -> pmid )

fk\_pmid


Fk fk\_author\_list
tbl\_author ref tbl\_medline\_citation ( fk\_pmid -> pmid )

fk\_pmid


Fk fk\_chemical\_list
tbl\_chemical ref tbl\_medline\_citation ( fk\_pmid -> pmid )

fk\_pmid


Fk fk\_citation\_subsets
tbl\_citation\_subset ref tbl\_medline\_citation ( fk\_pmid -> pmid )

fk\_pmid


Fk fk\_comments\_corrections
tbl\_comments\_correction ref tbl\_medline\_citation ( fk\_pmid -> pmid )

fk\_pmid


Fk fk\_data\_bank\_list
tbl\_data\_bank ref tbl\_medline\_citation ( fk\_pmid -> pmid )

fk\_pmid


Fk fk\_gene\_symbol\_list
tbl\_gene\_symbol ref tbl\_medline\_citation ( fk\_pmid -> pmid )

fk\_pmid


Fk fk\_general\_notes
tbl\_general\_note ref tbl\_medline\_citation ( fk\_pmid -> pmid )

fk\_pmid


Fk fk\_grant\_list
tbl\_grant ref tbl\_medline\_citation ( fk\_pmid -> pmid )

fk\_pmid


Fk fk\_investigator\_list
tbl\_investigator ref tbl\_medline\_citation ( fk\_pmid -> pmid )

fk\_pmid


Fk tbl\_journal\_fk\_pmid\_fkey
tbl\_journal ref tbl\_medline\_citation ( fk\_pmid -> pmid )

fk\_pmid


Fk fk\_keyword\_list
tbl\_keyword ref tbl\_medline\_citation ( fk\_pmid -> pmid )

fk\_pmid


Fk fk\_languages
tbl\_language ref tbl\_medline\_citation ( fk\_pmid -> pmid )

fk\_pmid


Fk fk\_medline\_journal\_info
tbl\_medline\_journal\_info ref tbl\_medline\_citation ( fk\_pmid -> pmid )

fk\_pmid


Fk fk\_mesh\_heading\_list
tbl\_mesh\_heading ref tbl\_medline\_citation ( fk\_pmid -> pmid )

fk\_pmid


Fk fk\_other\_ids
tbl\_other\_id ref tbl\_medline\_citation ( fk\_pmid -> pmid )

fk\_pmid


Fk fk\_personal\_name\_subject\_list
tbl\_personal\_name\_subject ref tbl\_medline\_citation ( fk\_pmid -> pmid )

fk\_pmid


Fk fk2\_pmids\_in\_file
tbl\_pmids\_in\_file ref tbl\_medline\_citation ( fk\_pmid -> pmid )

fk\_pmid


Fk fk3\_pmids\_in\_file
tbl\_pmids\_in\_file ref tbl\_xml\_file ( id\_file -> id, xml\_file\_name )

id\_file,xml\_file\_name


Fk fk\_publication\_type\_list
tbl\_publication\_type ref tbl\_medline\_citation ( fk\_pmid -> pmid )

fk\_pmid


Fk fk\_qualifier\_names
tbl\_qualifier\_name ref tbl\_medline\_citation ( fk\_pmid -> pmid )

fk\_pmid


Fk fk\_space\_flight\_missions
tbl\_space\_flight\_mission ref tbl\_medline\_citation ( fk\_pmid -> pmid )

fk\_pmid


tbl\_abstractTable pubmed.tbl\_abstract
Pk tbl\_abstract\_pkey ( fk\_pmid ) 
fk\_pmidfk\_pmid
\* integer
References tbl\_medline\_citation ( fk\_pmid -> pmid ) 
abstract\_textabstract\_text
text
copyright\_informationcopyright\_information
varchar(2000)


tbl\_accession\_numberTable pubmed.tbl\_accession\_number
Pk tbl\_accession\_number\_pkey ( fk\_pmid, data\_bank\_name, accession\_number ) 
fk\_pmidfk\_pmid
\* integer
References tbl\_medline\_citation ( fk\_pmid -> pmid ) 
Pk tbl\_accession\_number\_pkey ( fk\_pmid, data\_bank\_name, accession\_number ) ix\_pubmed\_tbl\_accession\_number\_data\_bank\_name ( data\_bank\_name ) 
data\_bank\_namedata\_bank\_name
\* varchar(300)
Pk tbl\_accession\_number\_pkey ( fk\_pmid, data\_bank\_name, accession\_number ) ix\_pubmed\_tbl\_accession\_number\_accession\_number ( accession\_number ) 
accession\_numberaccession\_number
\* varchar(100)


tbl\_authorTable pubmed.tbl\_author
Pk tbl\_author\_pkey ( id ) 
idid
\* serial
ix\_pubmed\_tbl\_author\_fk\_pmid ( fk\_pmid ) 
fk\_pmidfk\_pmid
\* integer
References tbl\_medline\_citation ( fk\_pmid -> pmid ) 
ix\_pubmed\_tbl\_author\_last\_name ( last\_name ) 
last\_namelast\_name
varchar(300)
fore\_namefore\_name
varchar(100)
initialsinitials
varchar(10)
suffixsuffix
varchar(10)
ix\_pubmed\_tbl\_author\_collective\_name ( collective\_name ) 
collective\_namecollective\_name
varchar(2000)


tbl\_chemicalTable pubmed.tbl\_chemical
Pk tbl\_chemical\_pkey ( fk\_pmid, registry\_number, name\_of\_substance ) 
fk\_pmidfk\_pmid
\* integer
References tbl\_medline\_citation ( fk\_pmid -> pmid ) 
Pk tbl\_chemical\_pkey ( fk\_pmid, registry\_number, name\_of\_substance ) 
registry\_numberregistry\_number
\* varchar(20)
Pk tbl\_chemical\_pkey ( fk\_pmid, registry\_number, name\_of\_substance ) ix\_pubmed\_tbl\_chemical\_name\_of\_substance ( name\_of\_substance ) 
name\_of\_substancename\_of\_substance
\* varchar(3000)


tbl\_citation\_subsetTable pubmed.tbl\_citation\_subset
Pk tbl\_citation\_subset\_pkey ( fk\_pmid, citation\_subset ) 
fk\_pmidfk\_pmid
\* integer
References tbl\_medline\_citation ( fk\_pmid -> pmid ) 
Pk tbl\_citation\_subset\_pkey ( fk\_pmid, citation\_subset ) 
citation\_subsetcitation\_subset
\* varchar(500)


tbl\_comments\_correctionTable pubmed.tbl\_comments\_correction
Pk tbl\_comments\_correction\_pkey ( id ) 
idid
\* serial
fk\_pmidfk\_pmid
\* integer
References tbl\_medline\_citation ( fk\_pmid -> pmid ) 
ref\_sourceref\_source
varchar(4000)
ref\_pmidref\_pmid
integer
notenote
varchar(4000)
typetype
\* varchar(30)


tbl\_data\_bankTable pubmed.tbl\_data\_bank
Pk tbl\_data\_bank\_pkey ( fk\_pmid, data\_bank\_name ) 
fk\_pmidfk\_pmid
\* integer
References tbl\_medline\_citation ( fk\_pmid -> pmid ) 
Pk tbl\_data\_bank\_pkey ( fk\_pmid, data\_bank\_name ) 
data\_bank\_namedata\_bank\_name
\* varchar(300)


tbl\_gene\_symbolTable pubmed.tbl\_gene\_symbol
Pk tbl\_gene\_symbol\_pkey ( fk\_pmid, gene\_symbol ) 
fk\_pmidfk\_pmid
\* integer
References tbl\_medline\_citation ( fk\_pmid -> pmid ) 
Pk tbl\_gene\_symbol\_pkey ( fk\_pmid, gene\_symbol ) ix\_pubmed\_tbl\_gene\_symbol\_gene\_symbol ( gene\_symbol ) 
gene\_symbolgene\_symbol
\* varchar(40)


tbl\_general\_noteTable pubmed.tbl\_general\_note
Pk tbl\_general\_note\_pkey ( fk\_pmid, general\_note ) 
fk\_pmidfk\_pmid
\* integer
References tbl\_medline\_citation ( fk\_pmid -> pmid ) 
Pk tbl\_general\_note\_pkey ( fk\_pmid, general\_note ) 
general\_notegeneral\_note
\* varchar(2000)
general\_note\_ownergeneral\_note\_owner
varchar(20)


tbl\_grantTable pubmed.tbl\_grant
Pk tbl\_grant\_pkey ( id ) 
idid
\* serial
ix\_pubmed\_tbl\_grant\_fk\_pmid ( fk\_pmid ) 
fk\_pmidfk\_pmid
\* integer
References tbl\_medline\_citation ( fk\_pmid -> pmid ) 
ix\_pubmed\_tbl\_grant\_grantid ( grantid ) 
grantidgrantid
varchar(200)
acronymacronym
varchar(20)
agencyagency
varchar(200)
countrycountry
varchar(200)


tbl\_investigatorTable pubmed.tbl\_investigator
Pk tbl\_investigator\_pkey ( id ) 
idid
\* serial
fk\_pmidfk\_pmid
\* integer
References tbl\_medline\_citation ( fk\_pmid -> pmid ) 
ix\_pubmed\_tbl\_investigator\_last\_name ( last\_name ) 
last\_namelast\_name
varchar(300)
fore\_namefore\_name
varchar(100)
initialsinitials
varchar(10)
suffixsuffix
varchar(10)
investigator\_affiliationinvestigator\_affiliation
varchar(200)


tbl\_journalTable pubmed.tbl\_journal
Pk tbl\_journal\_pkey ( fk\_pmid ) 
fk\_pmidfk\_pmid
\* integer
References tbl\_medline\_citation ( fk\_pmid -> pmid ) 
ix\_pubmed\_tbl\_journal\_issn ( issn ) 
issnissn
varchar(30)
issn\_typeissn\_type
varchar(30)
volumevolume
varchar(200)
issueissue
varchar(200)
ix\_pubmed\_tbl\_journal\_pub\_date\_year ( pub\_date\_year ) 
pub\_date\_yearpub\_date\_year
integer
pub\_date\_monthpub\_date\_month
varchar(20)
pub\_date\_daypub\_date\_day
varchar(2)
medline\_datemedline\_date
varchar(40)
titletitle
varchar(2000)
iso\_abbreviationiso\_abbreviation
varchar(100)


tbl\_keywordTable pubmed.tbl\_keyword
Pk tbl\_keyword\_pkey ( fk\_pmid, keyword ) 
fk\_pmidfk\_pmid
\* integer
References tbl\_medline\_citation ( fk\_pmid -> pmid ) 
Pk tbl\_keyword\_pkey ( fk\_pmid, keyword ) ix\_pubmed\_tbl\_keyword\_keyword ( keyword ) 
keywordkeyword
\* varchar(500)
keyword\_major\_ynkeyword\_major\_yn
char(1)


tbl\_languageTable pubmed.tbl\_language
Pk tbl\_language\_pkey ( fk\_pmid, language ) 
fk\_pmidfk\_pmid
\* integer
References tbl\_medline\_citation ( fk\_pmid -> pmid ) 
Pk tbl\_language\_pkey ( fk\_pmid, language ) 
languagelanguage
\* varchar(50)


tbl\_medline\_citationTable pubmed.tbl\_medline\_citation
Pk tbl\_medline\_citation\_pkey ( pmid ) 
pmidpmid
\* serial
Referred by tbl\_pmids\_in\_file ( fk\_pmid -> pmid )
Referred by tbl\_abstract ( fk\_pmid -> pmid )
Referred by tbl\_accession\_number ( fk\_pmid -> pmid )
Referred by tbl\_author ( fk\_pmid -> pmid )
Referred by tbl\_chemical ( fk\_pmid -> pmid )
Referred by tbl\_citation\_subset ( fk\_pmid -> pmid )
Referred by tbl\_comments\_correction ( fk\_pmid -> pmid )
Referred by tbl\_data\_bank ( fk\_pmid -> pmid )
Referred by tbl\_gene\_symbol ( fk\_pmid -> pmid )
Referred by tbl\_general\_note ( fk\_pmid -> pmid )
Referred by tbl\_grant ( fk\_pmid -> pmid )
Referred by tbl\_investigator ( fk\_pmid -> pmid )
Referred by tbl\_keyword ( fk\_pmid -> pmid )
Referred by tbl\_language ( fk\_pmid -> pmid )
Referred by tbl\_medline\_journal\_info ( fk\_pmid -> pmid )
Referred by tbl\_mesh\_heading ( fk\_pmid -> pmid )
Referred by tbl\_other\_id ( fk\_pmid -> pmid )
Referred by tbl\_personal\_name\_subject ( fk\_pmid -> pmid )
Referred by tbl\_publication\_type ( fk\_pmid -> pmid )
Referred by tbl\_qualifier\_name ( fk\_pmid -> pmid )
Referred by tbl\_space\_flight\_mission ( fk\_pmid -> pmid )
Referred by tbl\_journal ( fk\_pmid -> pmid ) 
date\_createddate\_created
date
ix\_pubmed\_tbl\_medline\_citation\_date\_completed ( date\_completed ) 
date\_completeddate\_completed
date
ix\_pubmed\_tbl\_medline\_citation\_date\_revised ( date\_revised ) 
date\_reviseddate\_revised
date
number\_of\_referencesnumber\_of\_references
integer
keyword\_list\_ownerkeyword\_list\_owner
varchar(30)
citation\_ownercitation\_owner
varchar(30)
citation\_statuscitation\_status
varchar(50)
article\_titlearticle\_title
\* varchar(4000)
start\_pagestart\_page
varchar(10)
end\_pageend\_page
varchar(10)
medline\_pgnmedline\_pgn
varchar(200)
article\_affiliationarticle\_affiliation
varchar(2000)
article\_author\_list\_comp\_ynarticle\_author\_list\_comp\_yn
char(1)
data\_bank\_list\_complete\_yndata\_bank\_list\_complete\_yn
char(1)
grant\_list\_complete\_yngrant\_list\_complete\_yn
char(1)
vernacular\_titlevernacular\_title
varchar(4000)


tbl\_medline\_journal\_infoTable pubmed.tbl\_medline\_journal\_info
Pk tbl\_medline\_journal\_info\_pkey ( fk\_pmid ) 
fk\_pmidfk\_pmid
\* integer
References tbl\_medline\_citation ( fk\_pmid -> pmid ) 
ix\_pubmed\_tbl\_medline\_journal\_info\_nlm\_unique\_id ( nlm\_unique\_id ) 
nlm\_unique\_idnlm\_unique\_id
varchar(20)
ix\_pubmed\_tbl\_medline\_journal\_info\_medline\_ta ( medline\_ta ) 
medline\_tamedline\_ta
\* varchar(200)
countrycountry
varchar(50)


tbl\_mesh\_headingTable pubmed.tbl\_mesh\_heading
Pk tbl\_mesh\_heading\_pkey ( fk\_pmid, descriptor\_name ) 
fk\_pmidfk\_pmid
\* integer
References tbl\_medline\_citation ( fk\_pmid -> pmid ) 
Pk tbl\_mesh\_heading\_pkey ( fk\_pmid, descriptor\_name ) 
descriptor\_namedescriptor\_name
\* varchar(500)
descriptor\_name\_major\_yndescriptor\_name\_major\_yn
char(1)


tbl\_other\_idTable pubmed.tbl\_other\_id
Pk tbl\_other\_id\_pkey ( fk\_pmid, other\_id, other\_id\_source ) 
fk\_pmidfk\_pmid
\* integer
References tbl\_medline\_citation ( fk\_pmid -> pmid ) 
Pk tbl\_other\_id\_pkey ( fk\_pmid, other\_id, other\_id\_source ) 
other\_idother\_id
\* varchar(30)
Pk tbl\_other\_id\_pkey ( fk\_pmid, other\_id, other\_id\_source ) 
other\_id\_sourceother\_id\_source
\* varchar(20)


tbl\_personal\_name\_subjectTable pubmed.tbl\_personal\_name\_subject
Pk tbl\_personal\_name\_subject\_pkey ( id ) 
idid
\* serial
fk\_pmidfk\_pmid
\* integer
References tbl\_medline\_citation ( fk\_pmid -> pmid ) 
ix\_pubmed\_tbl\_personal\_name\_subject\_last\_name ( last\_name ) 
last\_namelast\_name
\* varchar(300)
fore\_namefore\_name
varchar(100)
initialsinitials
varchar(10)
suffixsuffix
varchar(20)


tbl\_pmids\_in\_fileTable pubmed.tbl\_pmids\_in\_file
Pk tbl\_pmids\_in\_file\_pkey ( fk\_pmid ) 
fk\_pmidfk\_pmid
\* integer
References tbl\_medline\_citation ( fk\_pmid -> pmid ) 
id\_fileid\_file
integer
References tbl\_xml\_file ( id\_file -> id, xml\_file\_name ) 
xml\_file\_namexml\_file\_name
\* varchar(50)
References tbl\_xml\_file ( id\_file -> id, xml\_file\_name ) 


tbl\_publication\_typeTable pubmed.tbl\_publication\_type
Pk tbl\_publication\_type\_pkey ( fk\_pmid, publication\_type ) 
fk\_pmidfk\_pmid
\* integer
References tbl\_medline\_citation ( fk\_pmid -> pmid ) 
Pk tbl\_publication\_type\_pkey ( fk\_pmid, publication\_type ) 
publication\_typepublication\_type
\* varchar(200)


tbl\_qualifier\_nameTable pubmed.tbl\_qualifier\_name
Pk tbl\_qualifier\_name\_pkey ( fk\_pmid, descriptor\_name, qualifier\_name ) 
fk\_pmidfk\_pmid
\* integer
References tbl\_medline\_citation ( fk\_pmid -> pmid ) 
Pk tbl\_qualifier\_name\_pkey ( fk\_pmid, descriptor\_name, qualifier\_name ) ix\_pubmed\_tbl\_qualifier\_name\_descriptor\_name ( descriptor\_name ) 
descriptor\_namedescriptor\_name
\* varchar(500)
Pk tbl\_qualifier\_name\_pkey ( fk\_pmid, descriptor\_name, qualifier\_name ) ix\_pubmed\_tbl\_qualifier\_name\_qualifier\_name ( qualifier\_name ) 
qualifier\_namequalifier\_name
\* varchar(500)
qualifier\_name\_major\_ynqualifier\_name\_major\_yn
char(1)


tbl\_space\_flight\_missionTable pubmed.tbl\_space\_flight\_mission
Pk tbl\_space\_flight\_mission\_pkey ( fk\_pmid, space\_flight\_mission ) 
fk\_pmidfk\_pmid
\* integer
References tbl\_medline\_citation ( fk\_pmid -> pmid ) 
Pk tbl\_space\_flight\_mission\_pkey ( fk\_pmid, space\_flight\_mission ) 
space\_flight\_missionspace\_flight\_mission
\* varchar(500)


tbl\_xml\_fileTable pubmed.tbl\_xml\_file
Pk tbl\_xml\_file\_pkey ( id, xml\_file\_name ) 
idid
\* serial
Referred by tbl\_pmids\_in\_file ( id\_file -> id, xml\_file\_name ) 
Pk tbl\_xml\_file\_pkey ( id, xml\_file\_name ) 
xml\_file\_namexml\_file\_name
\* varchar(50)
Referred by tbl\_pmids\_in\_file ( id\_file -> id, xml\_file\_name ) 
doc\_type\_namedoc\_type\_name
varchar(100)
dtd\_public\_iddtd\_public\_id
varchar(200)
dtd\_system\_iddtd\_system\_id
varchar(200)
time\_processedtime\_processed
timestamp
  
  

| Table tbl\_abstract | | | |
| --- | --- | --- | --- |
| \* | fk\_pmid | integer |  |
|  | abstract\_text | text |  |
|  | copyright\_information | varchar( 2000 ) |  |
| **Indexes** | | | |
| Pk | tbl\_abstract\_pkey | ON fk\_pmid |  |
| **Foreign Keys** | | | |
|  | fk\_abstract | ( fk\_pmid ) ref tbl\_medline\_citation (pmid) |  |

  
  

| Table tbl\_accession\_number | | | |
| --- | --- | --- | --- |
| \* | fk\_pmid | integer |  |
| \* | data\_bank\_name | varchar( 300 ) |  |
| \* | accession\_number | varchar( 100 ) |  |
| **Indexes** | | | |
| Pk | tbl\_accession\_number\_pkey | ON fk\_pmid, data\_bank\_name, accession\_number |  |
|  | ix\_pubmed\_tbl\_accession\_number\_accession\_number | ON accession\_number |  |
|  | ix\_pubmed\_tbl\_accession\_number\_data\_bank\_name | ON data\_bank\_name |  |
| **Foreign Keys** | | | |
|  | fk\_accession\_number\_list | ( fk\_pmid ) ref tbl\_medline\_citation (pmid) |  |

  
  

| Table tbl\_author | | | |
| --- | --- | --- | --- |
| \* | id | serial |  |
| \* | fk\_pmid | integer |  |
|  | last\_name | varchar( 300 ) |  |
|  | fore\_name | varchar( 100 ) |  |
|  | initials | varchar( 10 ) |  |
|  | suffix | varchar( 10 ) |  |
|  | collective\_name | varchar( 2000 ) |  |
| **Indexes** | | | |
| Pk | tbl\_author\_pkey | ON id |  |
|  | ix\_pubmed\_tbl\_author\_collective\_name | ON collective\_name |  |
|  | ix\_pubmed\_tbl\_author\_fk\_pmid | ON fk\_pmid |  |
|  | ix\_pubmed\_tbl\_author\_last\_name | ON last\_name |  |
| **Foreign Keys** | | | |
|  | fk\_author\_list | ( fk\_pmid ) ref tbl\_medline\_citation (pmid) |  |

  
  

| Table tbl\_chemical | | | |
| --- | --- | --- | --- |
| \* | fk\_pmid | integer |  |
| \* | registry\_number | varchar( 20 ) |  |
| \* | name\_of\_substance | varchar( 3000 ) |  |
| **Indexes** | | | |
| Pk | tbl\_chemical\_pkey | ON fk\_pmid, registry\_number, name\_of\_substance |  |
|  | ix\_pubmed\_tbl\_chemical\_name\_of\_substance | ON name\_of\_substance |  |
| **Foreign Keys** | | | |
|  | fk\_chemical\_list | ( fk\_pmid ) ref tbl\_medline\_citation (pmid) |  |

  
  

| Table tbl\_citation\_subset | | | |
| --- | --- | --- | --- |
| \* | fk\_pmid | integer |  |
| \* | citation\_subset | varchar( 500 ) |  |
| **Indexes** | | | |
| Pk | tbl\_citation\_subset\_pkey | ON fk\_pmid, citation\_subset |  |
| **Foreign Keys** | | | |
|  | fk\_citation\_subsets | ( fk\_pmid ) ref tbl\_medline\_citation (pmid) |  |

  
  

| Table tbl\_comments\_correction | | | |
| --- | --- | --- | --- |
| \* | id | serial |  |
| \* | fk\_pmid | integer |  |
|  | ref\_source | varchar( 4000 ) |  |
|  | ref\_pmid | integer |  |
|  | note | varchar( 4000 ) |  |
| \* | type | varchar( 30 ) |  |
| **Indexes** | | | |
| Pk | tbl\_comments\_correction\_pkey | ON id |  |
| **Foreign Keys** | | | |
|  | fk\_comments\_corrections | ( fk\_pmid ) ref tbl\_medline\_citation (pmid) |  |
| **Constraints** | | | |
|  | ck1\_comments\_correction | (type)::text = ANY ((ARRAY['ErratumIn'::character varying, 'CommentOn'::character varying, 'CommentIn'::character varying, 'ErratumFor'::character varying, 'PartialRetractionIn'::character varying, 'PartialRetractionOf'::character varying, 'RepublishedFrom'::character varying, 'RepublishedIn'::character varying, 'RetractionOf'::character varying, 'RetractionIn'::character varying, 'UpdateIn'::character varying, 'UpdateOf'::character varying, 'SummaryForPatientsIn'::character varying, 'OriginalReportIn'::character varying, 'ReprintIn'::character varying, 'ReprintOf'::character varying])::text[]) |  |

  
  

| Table tbl\_data\_bank | | | |
| --- | --- | --- | --- |
| \* | fk\_pmid | integer |  |
| \* | data\_bank\_name | varchar( 300 ) |  |
| **Indexes** | | | |
| Pk | tbl\_data\_bank\_pkey | ON fk\_pmid, data\_bank\_name |  |
| **Foreign Keys** | | | |
|  | fk\_data\_bank\_list | ( fk\_pmid ) ref tbl\_medline\_citation (pmid) |  |

  
  

| Table tbl\_gene\_symbol | | | |
| --- | --- | --- | --- |
| \* | fk\_pmid | integer |  |
| \* | gene\_symbol | varchar( 40 ) |  |
| **Indexes** | | | |
| Pk | tbl\_gene\_symbol\_pkey | ON fk\_pmid, gene\_symbol |  |
|  | ix\_pubmed\_tbl\_gene\_symbol\_gene\_symbol | ON gene\_symbol |  |
| **Foreign Keys** | | | |
|  | fk\_gene\_symbol\_list | ( fk\_pmid ) ref tbl\_medline\_citation (pmid) |  |

  
  

| Table tbl\_general\_note | | | |
| --- | --- | --- | --- |
| \* | fk\_pmid | integer |  |
| \* | general\_note | varchar( 2000 ) |  |
|  | general\_note\_owner | varchar( 20 ) |  |
| **Indexes** | | | |
| Pk | tbl\_general\_note\_pkey | ON fk\_pmid, general\_note |  |
| **Foreign Keys** | | | |
|  | fk\_general\_notes | ( fk\_pmid ) ref tbl\_medline\_citation (pmid) |  |
| **Constraints** | | | |
|  | tbl\_general\_note\_general\_note\_owner\_check | (general\_note\_owner)::text = ANY ((ARRAY['NLM'::character varying, 'NASA'::character varying, 'PIP'::character varying, 'KIE'::character varying, 'HSR'::character varying, 'HMD'::character varying, 'SIS'::character varying, 'NOTNLM'::character varying])::text[]) |  |

  
  

| Table tbl\_grant | | | |
| --- | --- | --- | --- |
| \* | id | serial |  |
| \* | fk\_pmid | integer |  |
|  | grantid | varchar( 200 ) |  |
|  | acronym | varchar( 20 ) |  |
|  | agency | varchar( 200 ) |  |
|  | country | varchar( 200 ) |  |
| **Indexes** | | | |
| Pk | tbl\_grant\_pkey | ON id |  |
|  | ix\_pubmed\_tbl\_grant\_fk\_pmid | ON fk\_pmid |  |
|  | ix\_pubmed\_tbl\_grant\_grantid | ON grantid |  |
| **Foreign Keys** | | | |
|  | fk\_grant\_list | ( fk\_pmid ) ref tbl\_medline\_citation (pmid) |  |

  
  

| Table tbl\_investigator | | | |
| --- | --- | --- | --- |
| \* | id | serial |  |
| \* | fk\_pmid | integer |  |
|  | last\_name | varchar( 300 ) |  |
|  | fore\_name | varchar( 100 ) |  |
|  | initials | varchar( 10 ) |  |
|  | suffix | varchar( 10 ) |  |
|  | investigator\_affiliation | varchar( 200 ) |  |
| **Indexes** | | | |
| Pk | tbl\_investigator\_pkey | ON id |  |
|  | ix\_pubmed\_tbl\_investigator\_last\_name | ON last\_name |  |
| **Foreign Keys** | | | |
|  | fk\_investigator\_list | ( fk\_pmid ) ref tbl\_medline\_citation (pmid) |  |

  
  

| Table tbl\_journal | | | |
| --- | --- | --- | --- |
| \* | fk\_pmid | integer |  |
|  | issn | varchar( 30 ) |  |
|  | issn\_type | varchar( 30 ) |  |
|  | volume | varchar( 200 ) |  |
|  | issue | varchar( 200 ) |  |
|  | pub\_date\_year | integer |  |
|  | pub\_date\_month | varchar( 20 ) |  |
|  | pub\_date\_day | varchar( 2 ) |  |
|  | medline\_date | varchar( 40 ) |  |
|  | title | varchar( 2000 ) |  |
|  | iso\_abbreviation | varchar( 100 ) |  |
| **Indexes** | | | |
| Pk | tbl\_journal\_pkey | ON fk\_pmid |  |
|  | ix\_pubmed\_tbl\_journal\_issn | ON issn |  |
|  | ix\_pubmed\_tbl\_journal\_pub\_date\_year | ON pub\_date\_year |  |
| **Foreign Keys** | | | |
|  | tbl\_journal\_fk\_pmid\_fkey | ( fk\_pmid ) ref tbl\_medline\_citation (pmid) |  |

  
  

| Table tbl\_keyword | | | |
| --- | --- | --- | --- |
| \* | fk\_pmid | integer |  |
| \* | keyword | varchar( 500 ) |  |
|  | keyword\_major\_yn | char( 1 ) |  |
| **Indexes** | | | |
| Pk | tbl\_keyword\_pkey | ON fk\_pmid, keyword |  |
|  | ix\_pubmed\_tbl\_keyword\_keyword | ON keyword |  |
| **Foreign Keys** | | | |
|  | fk\_keyword\_list | ( fk\_pmid ) ref tbl\_medline\_citation (pmid) |  |
| **Constraints** | | | |
|  | ck1\_keyword\_list | keyword\_major\_yn = ANY (ARRAY['Y'::bpchar, 'N'::bpchar, 'y'::bpchar, 'n'::bpchar]) |  |

  
  

| Table tbl\_language | | | |
| --- | --- | --- | --- |
| \* | fk\_pmid | integer |  |
| \* | language | varchar( 50 ) |  |
| **Indexes** | | | |
| Pk | tbl\_language\_pkey | ON fk\_pmid, language |  |
| **Foreign Keys** | | | |
|  | fk\_languages | ( fk\_pmid ) ref tbl\_medline\_citation (pmid) |  |

  
  

| Table tbl\_medline\_citation | | | |
| --- | --- | --- | --- |
| \* | pmid | serial |  |
|  | date\_created | date |  |
|  | date\_completed | date |  |
|  | date\_revised | date |  |
|  | number\_of\_references | integer |  |
|  | keyword\_list\_owner | varchar( 30 ) |  |
|  | citation\_owner | varchar( 30 ) |  |
|  | citation\_status | varchar( 50 ) |  |
| \* | article\_title | varchar( 4000 ) |  |
|  | start\_page | varchar( 10 ) |  |
|  | end\_page | varchar( 10 ) |  |
|  | medline\_pgn | varchar( 200 ) |  |
|  | article\_affiliation | varchar( 2000 ) |  |
|  | article\_author\_list\_comp\_yn | char( 1 ) |  |
|  | data\_bank\_list\_complete\_yn | char( 1 ) |  |
|  | grant\_list\_complete\_yn | char( 1 ) |  |
|  | vernacular\_title | varchar( 4000 ) |  |
| **Indexes** | | | |
| Pk | tbl\_medline\_citation\_pkey | ON pmid |  |
|  | ix\_pubmed\_tbl\_medline\_citation\_date\_completed | ON date\_completed |  |
|  | ix\_pubmed\_tbl\_medline\_citation\_date\_revised | ON date\_revised |  |
| **Constraints** | | | |
|  | ck6\_medline\_citation | grant\_list\_complete\_yn = ANY (ARRAY['Y'::bpchar, 'N'::bpchar, 'y'::bpchar, 'n'::bpchar]) |  |
|  | ck5\_medline\_citation | data\_bank\_list\_complete\_yn = ANY (ARRAY['Y'::bpchar, 'N'::bpchar, 'y'::bpchar, 'n'::bpchar]) |  |
|  | ck4\_medline\_citation | article\_author\_list\_comp\_yn = ANY (ARRAY['Y'::bpchar, 'N'::bpchar, 'y'::bpchar, 'n'::bpchar]) |  |
|  | ck3\_medline\_citation | (citation\_status)::text = ANY ((ARRAY['In-Data-Review'::character varying, 'In-Process'::character varying, 'MEDLINE'::character varying, 'OLDMEDLINE'::character varying, 'PubMed-not-MEDLINE'::character varying, 'Publisher'::character varying, 'Completed'::character varying])::text[]) |  |
|  | ck2\_medline\_citation | (citation\_owner)::text = ANY ((ARRAY['NLM'::character varying, 'NASA'::character varying, 'PIP'::character varying, 'KIE'::character varying, 'HSR'::character varying, 'HMD'::character varying, 'SIS'::character varying, 'NOTNLM'::character varying])::text[]) |  |
|  | ck1\_medline\_citation | (keyword\_list\_owner)::text = ANY ((ARRAY['NLM'::character varying, 'NASA'::character varying, 'PIP'::character varying, 'KIE'::character varying, 'HSR'::character varying, 'HMD'::character varying, 'SIS'::character varying, 'NOTNLM'::character varying])::text[]) |  |

  
  

| Table tbl\_medline\_journal\_info | | | |
| --- | --- | --- | --- |
| \* | fk\_pmid | integer |  |
|  | nlm\_unique\_id | varchar( 20 ) |  |
| \* | medline\_ta | varchar( 200 ) |  |
|  | country | varchar( 50 ) |  |
| **Indexes** | | | |
| Pk | tbl\_medline\_journal\_info\_pkey | ON fk\_pmid |  |
|  | ix\_pubmed\_tbl\_medline\_journal\_info\_medline\_ta | ON medline\_ta |  |
|  | ix\_pubmed\_tbl\_medline\_journal\_info\_nlm\_unique\_id | ON nlm\_unique\_id |  |
| **Foreign Keys** | | | |
|  | fk\_medline\_journal\_info | ( fk\_pmid ) ref tbl\_medline\_citation (pmid) |  |

  
  

| Table tbl\_mesh\_heading | | | |
| --- | --- | --- | --- |
| \* | fk\_pmid | integer |  |
| \* | descriptor\_name | varchar( 500 ) |  |
|  | descriptor\_name\_major\_yn | char( 1 ) |  |
| **Indexes** | | | |
| Pk | tbl\_mesh\_heading\_pkey | ON fk\_pmid, descriptor\_name |  |
| **Foreign Keys** | | | |
|  | fk\_mesh\_heading\_list | ( fk\_pmid ) ref tbl\_medline\_citation (pmid) |  |
| **Constraints** | | | |
|  | ck1\_mesh\_heading\_list | descriptor\_name\_major\_yn = ANY (ARRAY['Y'::bpchar, 'N'::bpchar, 'y'::bpchar, 'n'::bpchar]) |  |

  
  

| Table tbl\_other\_id | | | |
| --- | --- | --- | --- |
| \* | fk\_pmid | integer |  |
| \* | other\_id | varchar( 30 ) |  |
| \* | other\_id\_source | varchar( 20 ) |  |
| **Indexes** | | | |
| Pk | tbl\_other\_id\_pkey | ON fk\_pmid, other\_id, other\_id\_source |  |
| **Foreign Keys** | | | |
|  | fk\_other\_ids | ( fk\_pmid ) ref tbl\_medline\_citation (pmid) |  |
| **Constraints** | | | |
|  | ck1\_other\_ids | (other\_id\_source)::text = ANY ((ARRAY['NASA'::character varying, 'KIE'::character varying, 'PIP'::character varying, 'POP'::character varying, 'ARPL'::character varying, 'CPC'::character varying, 'IND'::character varying, 'CPFH'::character varying, 'CLML'::character varying, 'IM'::character varying, 'SGC'::character varying, 'NLM'::character varying, 'NRCBL'::character varying, 'QCIM'::character varying, 'QCICL'::character varying])::text[]) |  |

  
  

| Table tbl\_personal\_name\_subject | | | |
| --- | --- | --- | --- |
| \* | id | serial |  |
| \* | fk\_pmid | integer |  |
| \* | last\_name | varchar( 300 ) |  |
|  | fore\_name | varchar( 100 ) |  |
|  | initials | varchar( 10 ) |  |
|  | suffix | varchar( 20 ) |  |
| **Indexes** | | | |
| Pk | tbl\_personal\_name\_subject\_pkey | ON id |  |
|  | ix\_pubmed\_tbl\_personal\_name\_subject\_last\_name | ON last\_name |  |
| **Foreign Keys** | | | |
|  | fk\_personal\_name\_subject\_list | ( fk\_pmid ) ref tbl\_medline\_citation (pmid) |  |

  
  

| Table tbl\_pmids\_in\_file | | | |
| --- | --- | --- | --- |
| \* | fk\_pmid | integer |  |
|  | id\_file | integer |  |
| \* | xml\_file\_name | varchar( 50 ) |  |
| **Indexes** | | | |
| Pk | tbl\_pmids\_in\_file\_pkey | ON fk\_pmid |  |
| **Foreign Keys** | | | |
|  | fk2\_pmids\_in\_file | ( fk\_pmid ) ref tbl\_medline\_citation (pmid) |  |
|  | fk3\_pmids\_in\_file | ( id\_file, xml\_file\_name ) ref tbl\_xml\_file (id, xml\_file\_name) |  |

  
  

| Table tbl\_publication\_type | | | |
| --- | --- | --- | --- |
| \* | fk\_pmid | integer |  |
| \* | publication\_type | varchar( 200 ) |  |
| **Indexes** | | | |
| Pk | tbl\_publication\_type\_pkey | ON fk\_pmid, publication\_type |  |
| **Foreign Keys** | | | |
|  | fk\_publication\_type\_list | ( fk\_pmid ) ref tbl\_medline\_citation (pmid) |  |

  
  

| Table tbl\_qualifier\_name | | | |
| --- | --- | --- | --- |
| \* | fk\_pmid | integer |  |
| \* | descriptor\_name | varchar( 500 ) |  |
| \* | qualifier\_name | varchar( 500 ) |  |
|  | qualifier\_name\_major\_yn | char( 1 ) |  |
| **Indexes** | | | |
| Pk | tbl\_qualifier\_name\_pkey | ON fk\_pmid, descriptor\_name, qualifier\_name |  |
|  | ix\_pubmed\_tbl\_qualifier\_name\_descriptor\_name | ON descriptor\_name |  |
|  | ix\_pubmed\_tbl\_qualifier\_name\_qualifier\_name | ON qualifier\_name |  |
| **Foreign Keys** | | | |
|  | fk\_qualifier\_names | ( fk\_pmid ) ref tbl\_medline\_citation (pmid) |  |
| **Constraints** | | | |
|  | ck2\_qualifier\_names | qualifier\_name\_major\_yn = ANY (ARRAY['Y'::bpchar, 'N'::bpchar, 'y'::bpchar, 'n'::bpchar]) |  |

  
  

| Table tbl\_space\_flight\_mission | | | |
| --- | --- | --- | --- |
| \* | fk\_pmid | integer |  |
| \* | space\_flight\_mission | varchar( 500 ) |  |
| **Indexes** | | | |
| Pk | tbl\_space\_flight\_mission\_pkey | ON fk\_pmid, space\_flight\_mission |  |
| **Foreign Keys** | | | |
|  | fk\_space\_flight\_missions | ( fk\_pmid ) ref tbl\_medline\_citation (pmid) |  |

  
  

| Table tbl\_xml\_file | | | |
| --- | --- | --- | --- |
| \* | id | serial |  |
| \* | xml\_file\_name | varchar( 50 ) |  |
|  | doc\_type\_name | varchar( 100 ) |  |
|  | dtd\_public\_id | varchar( 200 ) |  |
|  | dtd\_system\_id | varchar( 200 ) |  |
|  | time\_processed | timestamp |  |
| **Indexes** | | | |
| Pk | tbl\_xml\_file\_pkey | ON id, xml\_file\_name |  |
