# Supplementary material for: PubMedPortable: A Framework for Supporting the Development of Text Mining Applications
Source: PLoS One. 2016 Oct 5;11(10):e0163794. doi: 10.1371/journal.pone.0163794 (PMC5051953; doi:10.1371/journal.pone.0163794)
Supplement: S1 File — (ZIP) [file pone.0163794.s001.zip › PubMedPortable-master/documentation/Quick_Install.html]

PubMedPortable


# PubMedPortable

- 1 Introduction
- 2 Download a Data Set
  - 2.1 Download with a Web Browser
  - 2.2 Download with a Web Browser and EFetch
  - 2.3 Download PubMed
- 3 Installation
  - 3.1 Operating System
    - 3.1.1 Ubuntu
    - 3.1.2 Fedora
  - 3.2 Creation of PostgreSQL superuser
  - 3.3 Installation with Docker
- 4 Build up a Relational Database in PostgreSQL
- 5 Build up a Full Text Index with Xapian and Search It
- 6 Contact
  - 6.1 License

# 1 Introduction

- PubMedPortable processes XML files that can be downloaded from NCBI and builds up a PostgreSQL database containing all abstracts of the data set (no full texts).
- Furthermore, PubMedPortable generates a full text index with Xapian such that titles, abstracts, keywords, MeSH terms, and substances can be queried with search terms.
- It is scalable to your system requirements using multiprocessing and can be modified easily to your personal needs.
- This documentation refers to a text mining example using the search term “pancreatic cancer”. The wiki contains many basic examples how to use PostgreSQL and Xapian as well as connecting both with small Python programmes. The default values in the scripts refer to the parameters given here, but all of them can be changed by the user.
- If the examples from this documentations are used, there will be around 745 MB of disk space needed. There are no other hardware requirements.
- Start by copying the whole project folder from GitHub to your local disk.

# 2 Download a Data Set

- After downloading your data set, you can insert the XML files in an extra directory in your project folder “data” or somewhere else. The only important thing is to use only one type of topic or XML files in one folder, because the programme will insert all files of type XML in a directory into the database given (next step: “Build up a Relational Database in PostgreSQL”).

## 2.1 Download with a Web Browser

- Type in the following URL into your browser and search for “pancreatic cancer”:

  > - http://www.ncbi.nlm.nih.gov/pubmed/
  > - The quote signs are important for the exact search.
  > - Click on “Send to:” and “File” and select “XML”.
  > - 16th April 2015: 23258 PubMed-IDs
  > - The download of around 272 MB can take up to one hour depending on the time of day and internet connection.

## 2.2 Download with a Web Browser and EFetch

> - You can also download only the PubMed-ID list from your browser by selecting “PMID List” instead of “XML” and then use EFetch (tested only on Ubuntu):
>
>   > - Change into the directory “data” in your command-line and type in “python generate\_efetch.py”. It will build the script “efetch.sh” which generates XML files with 100 PubMed-IDs in each document.
>   > - The standard input filename is “pubmed\_result.txt” and the block size of PubMed-IDs for one XML file is 100, but this can be changed. Type in “python generate\_efetch.py -h” to show adjustable parameters.
>   > - If you want to specify the name of the directory where the XML files should be saved, use parameter “-d” and create this folder manually before.
>   > - The file “data/pubmed\_result.txt” contains 23258 PubMed-IDs and the first 100 PubMed-IDs are saved in “data/pancreatic\_cancer\_example/medline\_00000000.xml” as an example.
>   > - Make “efetch.sh” executable with “chmod +x efetch.sh” in the command-line and run it in a folder of your choice by typing in “./efetch.sh”.
>   > - This will be faster than using the browser to download a single XML file and it has the advantage of using multiprocessing in the next step as the script generates XML files with 100 titles in each document. The command also works with blocks of 500 PubMed-IDs, but not 1000.

## 2.3 Download PubMed

- It is also possible to download the whole PubMed via license:

  > - http://www.nlm.nih.gov/databases/journal.html
  > - http://www.nlm.nih.gov/bsd/licensee/2015\_stats/baseline\_doc.html

# 3 Installation

## 3.1 Operating System

- PubMedPortable was tested on Ubuntu and Fedora.

### 3.1.1 Ubuntu

- These are the packages that need to be installed to use PubMedPortable:

  > - python>=2.7.3
  > - postgresql>=8.4
  > - python-xappy>=0.5
  > - python-xapian>=1.2.8
  > - python-sqlalchemy>=0.9.7
  > - python-psycopg2>=2.4.5 (dependency from SQLAlchemy)
  > - To install, the following command can be used in the Ubuntu terminal:
  >
  >   > - “sudo apt-get install”
- If you use an older Ubuntu version, you can use “pip” to upgrade your package versions specifically for your user name, e.g.:

  > - “sudo pip install sqlalchemy –upgrade”

### 3.1.2 Fedora

- This section describes how to install required packages and how to adjust PostgreSQL settings in Fedora.
- To install the Fedora packages use the following command. It will install all required packages:

  > - “sudo -E dnf install python python-xappy python-sqlalchemy python-psycopg2 postgresql postgresql-server postgresql-contrib”
- To enable PostgreSQL in Fedora, use the following steps:

  > - “sudo systemctl enable postgresql”
  > - To start postgresql use the following command
  >
  >   > - “sudo systemctl start postgresql”
  > - To populate initial data, the following command is required:
  >
  >   > - “journalctl -xn”
  > - To initialise database, use the following command:
  >
  >   > - “sudo postgresql-setup initdb”
  > - To allow read access to postgres, SELinux should be modified. The SELinux is required upto Fedora 23. This can be done with the following command:
  >
  >   > - “grep postgres /var/log/audit/audit.log | audit2allow -M mypol”
  > - Then you can do this (this is also required upto Fedora 23):
  >
  >   > - “sudo semodule -i mypol.pp”
  > - Append this line in the file “pg\_hba.conf” (default location: “/var/lib/pgsql/data/pg\_hba.conf”):
  >
  >   > - “host all all 0.0.0.0 0.0.0.0 trust”
  >   > - If “trust” is used instead of “ident”, you are allowed to use a password. “0.0.0.0” means that all machines are allowed to login. That means, if you want to customise which server has to reach the database, you can control it here.

## 3.2 Creation of PostgreSQL superuser

- If there is not yet a superuser for the PostgreSQL database, create one with the name of your local account

  > - “sudo -u postgres createuser --superuser <user\_name>”
  > - “sudo -u <user\_name> psql template1”
  >
  >   > - \password <press enter, type in password, and press enter, again>
  >   > - \q
- Now, you can connect to the standard PostgreSQL database “postgres” with PGAdmin3 or via command-line:

  > - “psql -h localhost -d postgres -U <user\_name>”

## 3.3 Installation with Docker

- Docker is similar to a virtual machine, but it is easier to deploy and more efficient. It was tested in Ubuntu and Windows.
- You can use the PubMedPortable image to create a PostgreSQL relational database and a Xapian full text index without installing the packages mentioned above in basically two steps.
- Install Docker - it was tested on Ubuntu (64-bit required):

  > - https://docs.docker.com/installation/ubuntulinux/
  > - There are many different operating systems supported:
  >
  >   > - https://docs.docker.com/installation/#installation
- Run Docker with the PubMedPortable image:

  > - Create a folder on your local disk with a name of your choice.
  > - Go into that folder and create a directory “import\_data”.
  > - Copy you XML files downloaded from PubMed into the directory “import\_data”.
  > - Open a terminal and type in this command:
  >
  >   > - “sudo docker run -d -v /home/<user\_name>/<folder\_of\_your\_choice>/:/export/ -p 9999:5432 bgruening/pubmed2go”
  >   > - This will create the PostgreSQL folder as well as the full text index database folder within the <folder\_of\_your\_choice>.
  >   > - You can see that Docker is running by typing in “sudo docker ps”. This will show a randomly generated name for your process.
  >   > - Stopping Docker is possible by doing “sudo docker stop <name>”.
  >   > - Docker maps your PosgreSQL port “5432” to the port “9999”. Now, you can connect to your database with PGAdmin via “localhost”, port “9999” and user “parser” with password “parser”. If you want to connect via command-line, use this command:
  >   >
  >   >   > - “psql -h localhost -U parser -p 9999 -d pubmed”
  >   > - If you have created another folder with a name <folder\_of\_your\_choice> and the directory “import\_data”, you can create another database on port “9998” and another full text index with different data there:
  >   >
  >   >   > - “sudo docker run -d -v /home/<user\_name>/<folder\_of\_your\_choice>/:/export/ -p 9998:5432 bgruening/pubmed2go”
  >   > - In case of replacing or creating a database on a port that is already used, delete the complete directory <folder\_of\_your\_choice> and repeat the configuration steps.
- You can connect to PostgreSQL and Xapian with the programming language of your choice or follow the Python examples given in this documentation. If you want to develop your own text mining pipelines based on your data set of choice, you will have to install the required libraries on your operating system.
- This also means that you need a default PostgreSQL installation on your operating system. Restart a closed Docker session on port “9999” with the command:

  > - “sudo docker run -d -v /home/<user\_name>/<folder\_of\_your\_choice>/:/export/ -p 9999:5432 bgruening/pubmed2go”
- It is not recommended to run the PubMedPortable examples or to develop new scripts within the Docker container. If you want to modify the image, use the Docker documentation and this repository:

  > - https://github.com/bgruening/docker-recipes/tree/master/pubmed2go
- If you want to try the examples given in the wiki, copy the Xapian directory from the <folder\_of\_your\_choice> into the folder “PubMedPortable/full\_text\_index/xapian/” from “https://github.com/KerstenDoering/PubMedPortable” and run the Docker container in background.
- After successful installation, the rest of this documentation can be skipped.

# 4 Build up a Relational Database in PostgreSQL

- Open a Terminal and type in:

  > - “psql template1”
- Enter the following commands into psql prompt to create a database, the schema “pubmed”, and a standard user “parser”. It is important to write the user “parser” in single quotes in the creation step:

  > - CREATE USER parser WITH PASSWORD 'parser';
  > - CREATE DATABASE pancreatic\_cancer\_db;
  > - GRANT ALL PRIVILEGES ON DATABASE pancreatic\_cancer\_db to parser;
  > - \q
- Now you can create a schema “pubmed” as user “parser”. You will be asked to enter your password “parser” here:

  > - “psql -h localhost -d pancreatic\_cancer\_db -U parser -f create\_schema.sql”
- If you want to use another database name, just change “pancreatic\_cancer\_db” in these commands and provide this name in all other scripts by choosing the right parameter.
- It is recommended to use the name “parser” with password “parser” and the schema “pubmed”, because this is hard coded in “PubMedDB.py” and “PubMedParser.py”
- Create the tables in your database schema “pubmed” like this:

  > - Use the command “python PubMedDB.py -d pancreatic\_cancer\_db” in your terminal. There are no other parameters that can be set.
- Load the data from PubMed into your PostgreSQL database:

  > - You can check “python PubMedParser.py -h” to get a help screen with all adjustable parameters. If you want to use the defaults, you can simply type in “python PubMedParser.py”.
  >
  >   > - By default, previously in PostgreSQL inserted data will be deleted before loading the new XML files into the database. That means you just have to call “python PubMedParser.py”, again in case you want to load new data into your already created database.
  >   > - If you do not want to delete, but only add XML files to the data that is already inside your PostgreSQL database, use parameter “-c”.
  >   > - The default database name is “pancreatic\_cancer\_db” and the default number of processors is 2. For changing, use parameters “-d” and “-p”.
  >   > - If you want to process only part of your files, use the parameters “-s” and “-e” with numbers referring to your alphabetically sorted files, e.g. “-s 0 -e 20” for the first 20 XML files in the directory.
  > - It is important that you only type in the name of the folder containing all XML files with parameter “-i”, but not the name of the file(s). You do not need to type in the absolute path. Suppose, you have saved your XML file(s) in the directory “data/pancreatic\_cancer”, use this command to run it with 3 processors and the database “pancreatic\_cancer\_db”:
  >
  >   > - “python PubMedParser.py -i data/pancreatic\_cancer/ -d pancreatic\_cancer\_db -p 3”
  > - For one file with around 272 MB this takes around 10 min (only one processor can be used). For the same amount of data split into files with only 100 PubMed-IDs (use “generate\_efetch.py”) it takes around 4 min with 3 processors (2,83 GHz and 8 GB RAM).
- Now, a schema “pubmed” exists in your database “pancreatic\_cancer\_db” that contains all abstracts, titles, authors, etc. More information will be given in the wiki, containing SQL queries and small programming examples.

# 5 Build up a Full Text Index with Xapian and Search It

- The results from this section can be found in “full\_text\_index/results/results\_from\_documentation/”.
- Change into the directory “full\_text\_index” in your terminal.
- Create two directories, “xapian” and “results”, if they do not yet exist.
- Type in “python RunXapian.py -h” to get a help screen with all adjustable parameters.
- If you use all default values from this documentation, you will receive results in “results/results.csv” with “python RunXapian.py -x”.

  > - This command indexes all titles, abstracts, keywords, MeSH terms and substances from year 1809 to 2015, downloaded as XML files from PubMed (as described in section “Download a Data Set”).
  > - After completing the step of generating the full text index, the programme searches it with the synonyms given in “synonyms/pancreatic\_cancer.txt”.
  >
  >   > - This file contains manually chosen names of drugs, genes, proteins, and diseases related to pancreatic cancer, manually extracted from DrugBank (exact search with quotes for “pancreatic cancer”) and OMIM:
  >   >
  >   >   > - http://www.drugbank.ca/
  >   >   > - http://omim.org/entry/260350?search=%22pancreatic%20cancer%22
  > - If you just want to index your XML files, type in “python RunXapian.py -x -f”. (Parameter “-f” turns off the search function of the programme, default is “True”.)
  > - If you just want to search your synonyms, type in “python RunXapian.py” (Parameter “-x” turns on the indexing step, default is “False”.)
  > - The default location for your full text index database folder is “PubMedPortable/full\_text\_index/xapian/<xapian2015>”. You can change this location by using the parameter “-p”.
- For the given example, 10392 lines were generated in “results.csv”.

# 6 Contact

- Please, write an e-mail, if you have questions, feedback, improvements, or new ideas:

  > - kersten.doering at gmail dot com
- If you are interested in related projects, visit our working group’s homepage:

  > - http://www.pharmaceutical-bioinformatics.de

## 6.1 License

- PubMedPortable is published with an ISC license given in “license.txt”.
