# Supplementary material for: PubMedPortable: A Framework for Supporting the Development of Text Mining Applications
Source: PLoS One. 2016 Oct 5;11(10):e0163794. doi: 10.1371/journal.pone.0163794 (PMC5051953; doi:10.1371/journal.pone.0163794)
Supplement: S1 File — (ZIP) [file pone.0163794.s001.zip › PubMedPortable-master/documentation/Wiki.html]

PubMedPortable


# PubMedPortable

- 1 Introduction
- 2 Download a Data Set
- 3 Installation
  - 3.1 Operating System
    - 3.1.1 Ubuntu
    - 3.1.2 Fedora
  - 3.2 Creation of PostgreSQL superuser
  - 3.3 Installation with Docker
- 4 Build up a Relational Database in PostgreSQL
- 5 Build up a Full Text Index with Xapian and Search It
- 6 Examples for Using Full Text Search and Selecting Data from PostgreSQL
  - 6.1 Xapian
  - 6.2 PostgreSQL
  - 6.3 PostgreSQL and Xapian
- 7 Examples for Generating Plots
  - 7.1 Word Cloud
  - 7.2 Pie Chart
  - 7.3 Bar Chart
- 8 Examples for Using BioC and PubTator
- 9 Indexing of PMC Articles
  - 9.1 Introduction
  - 9.2 Get PMC Files
  - 9.3 Create Tables and Xapian Index
- 10 Named Entity Recognition Tools
- 11 Lucene as an Alternative to Xapian
  - 11.1 Installation
  - 11.2 Usage
- 12 Contact
  - 12.1 License

# 1 Introduction

- PubMedPortable processes XML files that can be downloaded from NCBI and builds up a PostgreSQL database containing all abstracts of the data set (no full texts).
- Furthermore, PubMedPortable generates a full text index with Xapian such that titles, abstracts, keywords, MeSH terms and substances can be queried with search terms.
- It is scalable to your system requirements using multiprocessing and can be modified easily to your personal needs.
- This documentation refers to a text mining example referring to the disease pancreatic cancer. It contains many examples how to use PostgreSQL and Xapian as well as connecting both with small Python programmes. The default values in the scripts refer to the parameters given here, but all of them can be changed by the user.
- Pancreatic cancer is one of the most dangerous cancer types, because the only way to cure a patient is a surgery beside several therapeutic strategies that could not significantly increase survival rates [Pancreatic cancer: from state-of-the-art treatments to promising novel therapies. Garrido-Laguna I, Hidalgo M. Nat Rev Clin Oncol. 2015 Mar 31. doi: 10.1038/nrclinonc.2015.53.].
- Considering text mining as an approach to have a closer look at related genes/proteins, chemical compounds, and diseases in documents that are relevant for pancreatic cancer shows a need for an easy-to-use software bridging the gap between processing data sets from PubMed effectively on a local machine and using the range of tools offered for natural language processing, e.g. by the BioCreative community. PubMedPortable offers such a solution.
- If the examples from this documentations are used, there will be around 745 MB of disk space needed. There are no other hardware requirements.
- Start by copying the whole project folder from GitHub to your local disk.

# 2 Download a Data Set

- Type in the following URL into your browser and search for “pancreatic cancer”:

  > - http://www.ncbi.nlm.nih.gov/pubmed/
  > - The quote signs are important for the exact search.
  > - Click on “Send to:” and “File” and select “XML”.
  > - 16th April 2015: 23258 PubMed-IDs
  >
  >   > - Some PubMed-IDs might change over time. Even for the given example list of PubMed-IDs for this documentation in “data/pubmed\_result.txt” it is possible, that you receive another number of downloaded publications in your XML files as well as different outcomes in the ongoing analyses.
  >   > - You can also use PubMedPortable for daily updates, but the parser will not include PubMed IDs which are already contained in the database. Therefore, we recommend to generate a completely new database and a new full text index once a year.
  > - The download of around 272 MB can take up to one hour depending on the time of day and internet connection.
- Another possibility is to download PubMed articles with EFetch:

  > - How to build a query:
  >
  >   > - http://www.ncbi.nlm.nih.gov/books/NBK25499/#chapter4.EFetch
  >   > - http://www.ncbi.nlm.nih.gov/books/NBK25501/
  > - You can also download only the PubMed-ID list from your browser by selecting “PMID List” instead of “XML” and then use EFetch (tested only on Ubuntu):
  >
  >   > - Change into the directory “data” in your command-line and type in “python generate\_efetch.py”. It will build the script “efetch.sh” which generates XML files with 100 PubMed-IDs in each document.
  >   > - The standard input filename is “pubmed\_result.txt” and the block size of PubMed-IDs for one XML file is 100, but this can be changed. Type in “python generate\_efetch.py -h” to show adjustable parameters.
  >   > - If you want to specify the name of the directory where the XML files should be saved, use parameter “-d” and create this folder manually before.
  >   > - The file “data/pubmed\_result.txt” contains 23258 PubMed-IDs and the first 100 PubMed-IDs are saved in “data/pancreatic\_cancer\_example/medline\_00000000.xml” as an example.
  >   > - Make “efetch.sh” executable with “chmod +x efetch.sh” in the command-line and run it in a folder of your choice by typing in “./efetch.sh”.
  >   > - This will be faster than using the browser to download a single XML file and it has the advantage of using multiprocessing in the next step as the script generates XML files with 100 titles in each document. The command also works with blocks of 500 PubMed-IDs, but not 1000.
- It is also possible to download the whole PubMed via license:

  > - http://www.nlm.nih.gov/databases/journal.html
  > - http://www.nlm.nih.gov/bsd/licensee/2015\_stats/baseline\_doc.html
- You can insert the XML files in an extra directory in your project folder “data” or somewhere else. The only important thing is to use only one type of topic or XML files in one folder, because the programme will insert all files of type XML in a directory into the database given (next step).

# 3 Installation

## 3.1 Operating System

- PubMedPortable was tested on Ubuntu and Fedora.

### 3.1.1 Ubuntu

- These are the packages that need to be installed to use PubMedPortable:

  > - python>=2.7.3
  > - postgresql>=8.4
  > - python-xappy>=0.5
  > - python-xapian>=1.2.8
  > - python-sqlalchemy>=0.9.7
  > - python-psycopg2>=2.4.5 (dependency from SQLAlchemy)
  > - To install, the following command can be used in the Ubuntu terminal:
  >
  >   > - “sudo apt-get install”
- If you use an older Ubuntu version, you can use “pip” to upgrade your package versions specifically for your user name, e.g.:

  > - “sudo pip install sqlalchemy –upgrade”

### 3.1.2 Fedora

- This section describes how to install required packages and how to adjust PostgreSQL settings in Fedora.
- To install the Fedora packages use the following command. It will install all required packages:

  > - “sudo -E dnf install python python-xappy python-sqlalchemy python-psycopg2 postgresql postgresql-server postgresql-contrib”
- To enable PostgreSQL in Fedora, use the following steps:

  > - “sudo systemctl enable postgresql”
  > - To start postgresql use the following command
  >
  >   > - “sudo systemctl start postgresql”
  > - To populate initial data, the following command is required:
  >
  >   > - “journalctl -xn”
  > - To initialise database, use the following command:
  >
  >   > - “sudo postgresql-setup initdb”
  > - To allow read access to postgres, SELinux should be modified. This can be done with the following command:
  >
  >   > - “grep postgres /var/log/audit/audit.log | audit2allow -M mypol”
  > - Then you can do this (this is also required upto Fedora 23):
  >
  >   > - “sudo semodule -i mypol.pp”
  > - Append this line in the file “pg\_hba.conf” (default location: “/var/lib/pgsql/data/pg\_hba.conf”):
  >
  >   > - “host all all 0.0.0.0 0.0.0.0 trust”
  >   > - If “trust” is used instead of “ident”, you are allowed to use a password. “0.0.0.0” means that all machines are allowed to login. That means, if you want to customise which server has to reach the database, you can control it here.

## 3.2 Creation of PostgreSQL superuser

- If there is not yet a superuser for the PostgreSQL database, create one with the name of your local account

  > - “sudo -u postgres createuser --superuser <user\_name>”
  > - “sudo -u <user\_name> psql template1”
  >
  >   > - \password <press enter, type in password, and press enter, again>
  >   > - \q
- Now, you can connect to the standard PostgreSQL database “postgres” with PGAdmin3 or via command-line:

  > - “psql -h localhost -d postgres -U <user\_name>”

## 3.3 Installation with Docker

- Docker is similar to a virtual machine, but it is easier to deploy and more efficient. It was tested in Ubuntu and Windows.
- You can use the PubMedPortable image to create a PostgreSQL relational database and a Xapian full text index without installing the packages mentioned above in basically two steps.
- Install Docker - it was tested on Ubuntu (64-bit required):

  > - https://docs.docker.com/installation/ubuntulinux/
  > - There are many different operating systems supported:
  >
  >   > - https://docs.docker.com/installation/#installation
- Run Docker with the PubMedPortable image:

  > - Create a folder on your local disk with a name of your choice.
  > - Go into that folder and create a directory “import\_data”.
  > - Copy you XML files downloaded from PubMed into the directory “import\_data”.
  > - Open a terminal and type in this command:
  >
  >   > - “sudo docker run -d -v /home/<user\_name>/<folder\_of\_your\_choice>/:/export/ -p 9999:5432 bgruening/pubmed2go”
  >   > - This will create the PostgreSQL folder as well as the full text index database folder within the <folder\_of\_your\_choice>.
  >   > - You can see that Docker is running by typing in “sudo docker ps”. This will show a randomly generated name for your process.
  >   > - Stopping Docker is possible by doing “sudo docker stop <name>”.
  >   > - Docker maps your PosgreSQL port “5432” to the port “9999”. Now, you can connect to your database with PGAdmin via “localhost”, port “9999” and user “parser” with password “parser”. If you want to connect via command-line, use this command:
  >   >
  >   >   > - “psql -h localhost -U parser -p 9999 -d pubmed”
  >   > - If you have created another folder with a name <folder\_of\_your\_choice> and the directory “import\_data”, you can create another database on port “9998” and another full text index with different data there:
  >   >
  >   >   > - “sudo docker run -d -v /home/<user\_name>/<folder\_of\_your\_choice>/:/export/ -p 9998:5432 bgruening/pubmed2go”
  >   > - In case of replacing or creating a database on a port that is already used, delete the complete directory <folder\_of\_your\_choice> and repeat the configuration steps.
- You can connect to PostgreSQL and Xapian with the programming language of your choice or follow the Python examples given in this documentation. If you want to develop your own text mining pipelines based on your data set of choice, you will have to install the required libraries on your operating system.
- This also means that you need a default PostgreSQL installation on your operating system. Restart a closed Docker session on port “9999” with the command:

  > - “sudo docker run -d -v /home/<user\_name>/<folder\_of\_your\_choice>/:/export/ -p 9999:5432 bgruening/pubmed2go”
- It is not recommended to run the PubMedPortable examples or to develop new scripts within the Docker container. If you want to modify the image, use the Docker documentation and this repository:

  > - https://github.com/bgruening/docker-recipes/tree/master/pubmed2go
- If you want to try the examples given in the sections 5 to 8, copy the Xapian directory from the <folder\_of\_your\_choice> into the folder “PubMedPortable/full\_text\_index/xapian/” from “https://github.com/KerstenDoering/PubMedPortable” and run the Docker container in background. In case of using Docker, you can completely skip section 4.

# 4 Build up a Relational Database in PostgreSQL

- Open a Terminal and type in:

  > - “psql template1”
- Enter the following commands into psql prompt to create a database, the schema “pubmed”, and a standard user “parser”. It is important to write the user “parser” in single quotes in the creation step:

  > - CREATE USER parser WITH PASSWORD 'parser';
  > - CREATE DATABASE pancreatic\_cancer\_db;
  > - GRANT ALL PRIVILEGES ON DATABASE pancreatic\_cancer\_db to parser;
  > - \q
- Now you can create a schema “pubmed” as user “parser”. You will be asked to enter your password “parser” here:

  > - “psql -h localhost -d pancreatic\_cancer\_db -U parser -f create\_schema.sql”
- If you want to use another database name, just change “pancreatic\_cancer\_db” in these commands and provide this name in all other scripts by choosing the right parameter.
- It is recommended to use the name “parser” with password “parser” and the schema “pubmed”, because this is hard coded in “PubMedDB.py” and “PubMedParser.py”
- Create the tables in your database schema “pubmed” like this:

  > - Use the command “python PubMedDB.py -d pancreatic\_cancer\_db” in your terminal. There are no other parameters that can be set.
- Load the data from PubMed into your PostgreSQL database:

  > - You can check “python PubMedParser.py -h” to get a help screen with all adjustable parameters. If you want to use the defaults, you can simply type in “python PubMedParser.py”.
  >
  >   > - By default, previously in PostgreSQL inserted data will be deleted before loading the new XML files into the database. That means you just have to call “python PubMedParser.py”, again in case you want to load new data into your already created database.
  >   > - If you do not want to delete, but only add XML files to the data that is already inside your PostgreSQL database, use parameter “-c”.
  >   > - The default database name is “pancreatic\_cancer\_db” and the default number of processors is 2. For changing, use parameters “-d” and “-p”.
  >   > - If you want to process only part of your files, use the parameters “-s” and “-e” with numbers referring to your alphabetically sorted files, e.g. “-s 0 -e 20” for the first 20 XML files in the directory.
  > - It is important that you only type in the name of the folder containing all XML files with parameter “-i”, but not the name of the file(s). You do not need to type in the absolute path. Suppose, you have saved your XML file(s) in the directory “data/pancreatic\_cancer”, use this command to run it with 3 processors and the database “pancreatic\_cancer\_db”:
  >
  >   > - “python PubMedParser.py -i data/pancreatic\_cancer/ -d pancreatic\_cancer\_db -p 3”
  > - If you receive an error concerning too many database connections, make sure that you use the latest version of SQLAlchemy. In earlier versions, sometimes the database connections were closed by the programme, but still remained open for some seconds, preventing the new programme to open a new connection. You can also increase the number of possible connections to your PostgreSQL server that can be opened (Ubuntu: “max\_connections = <type in number>” in “/etc/postgresql/<version number>/main/postgresql.conf”).
  > - For one file with around 272 MB this takes around 10 min (only one processor can be used). For the same amount of data split into files with only 100 PubMed-IDs (use “generate\_efetch.py”) it takes around 4 min with 3 processors (2,83 GHz and 8 GB RAM).
- Now, a schema “pubmed” exists in your database “pancreatic\_cancer\_db” that contains all abstracts, titles, authors, etc. More information will be given in section 5, containing SQL queries and small programming examples.
- The schema is described in the file “documentation/PostgreSQL\_database\_schema.html” which was generated with DbSchema (http://www.dbschema.com/download.html).
- If you want to extend the database schema in terms of additional columns or tables, you can have a look at this diff in the GitHub repository:

  > - https://github.com/KerstenDoering/PubMedPortable/commit/99f39f385c83d121422d1c48694c7fb2e6e421b3
  > - Consider the example of UI fields (MeSH IDs) for chemical substances.
  > - The column needs to be initialised (line 220 and 225 in PubMedDB.py) and the parser needs to get this XML field (line 309 in PubMedParser.py).
  > - The steps how to create a new table with adapted columns can be seen in the example of creating a class OtherID and OtherAbstract from the earlier existing class Other.

# 5 Build up a Full Text Index with Xapian and Search It

- The results from this section can be found in “full\_text\_index/results/results\_from\_documentation/”.
- Change into the directory “full\_text\_index” in your terminal.
- Create two directories, “xapian” and “results”, if they do not yet exist.
- Type in “python RunXapian.py -h” to get a help screen with all adjustable parameters.
- If you use all default values from this documentation, you will receive results in “results/results.csv” with “python RunXapian.py -x”.

  > - If you want to use the PostgreSQL database generated with Docker, change the port in the script “Article.py” in line 21 from “5432” to “9999” (the port you selected in Docker) and use the parameter “-d” with the database “pubmed” for the script “RunXapian.py”.
  > - This command indexes all titles, abstracts, keywords, MeSH terms and substances from year 1809 to 2015, downloaded as XML files from PubMed (as described in section “Download a Data Set”).
  >
  >   > - There are no abstracts with a publication date before 1809:
  >   > - http://www.nlm.nih.gov/bsd/licensee/2015\_stats/baseline\_med\_filecount.html
  > - After completing the step of generating the full text index, the programme searches it with the synonyms given in “synonyms/pancreatic\_cancer.txt”.
  >
  >   > - This file contains manually chosen names of drugs, genes, proteins, and diseases related to pancreatic cancer.
  >   > - User-provided synonyms can be directly stored in this file or saved in a new text document in the folder “synonyms”. Subsequently, the parameter “-s” can be used to process this file.
  > - The output in the command-line shows how many PubMed-IDs are indexed (23258) and how many synonyms are searched (86).
  > - This takes around 2-3 min on a 2,83 GHz machine with 8 GB RAM.
  > - You can also select single years for indexing and searching.
  > - If you just want to index your XML files, type in “python RunXapian.py -x -f”. (Parameter “-f” turns off the search function of the programme, default is “True”.)
  > - If you just want to search your synonyms, type in “python RunXapian.py” (Parameter “-x” turns on the indexing step, default is “False”.)
  > - The default location for your full text index database folder is “PubMedPortable/full\_text\_index/xapian/<xapian2015>”. You can change this location by using the parameter “-p”.
- For the given example, 10392 lines were generated in “results.csv”. Run “python summary.py” to get two CSV files in directory “results”. If you have chosen another filename as output from “RunXapian.py”, you can do “python summary.py -f <name\_of\_input\_file.csv>”:

  > - Drug synonyms were taken from DrugBank using the exact search query “pancreatic cancer”:
  >
  >   > - http://www.drugbank.ca/
  > - Protein and gene synonyms have been extracted manually from OMIM also performing an exact search:
  >
  >   > - http://omim.org/entry/260350?search=%22pancreatic%20cancer%22
  > - Diseases related to pancreatic cancer have been taken the text given on OMIM, too.
  > - “counts\_results.csv” shows how many synonyms were found (descending - 64 lines, meaning 64 from a total of 86 search terms). The alternative input filename will be “counts\_<input\_file.csv>”.
  >
  >   > - Taking into account the drugs, gemcitabine shows the most hits (2907). Erlotinib was found in 311 publications. Other approved drugs like WF10 and hydroxocobalamin were not found. Many investigational drugs were found 1-10 times: R115777, G17DT, hedgehog pathway inhibitor, imexon, GV1001, RP101, MGI-114, and PX-12. No other substances given on DrugBank were identified in this data set.
  >   > - Pancreatic ductal adenocarcinoma is the most common type of pancreatic cancer ( http://www.cancer.gov/aboutnci/budget\_planning\_leg/plan-2013/profiles/pancreatic ), which is shown by the 1598 hits. The tumor suppressor protein p53 was found 660 times, but also associated genes like KRAS, SMAD4, BRCA2, mTOR and CDKN2A were found (138-424 times). Many other genes were identified with a number below 10 hits and can be further analysed in “pmids\_results.csv”.
  >   > - Associated diseases like breast cancer, colon cancer, ovarian cancer and diabetes were found 255-919 times.
  > - “pmids\_results.csv” shows which synonyms co-occur in the same abstract or title, sorted by PubMed-IDs (7500 lines). In case of an alternative input filename, there will be the resulting file “pmids\_<input\_file.csv>”.
- In case, you want to index the whole PubMed, it can be useful to index blocks of years or every year as a single directory. Like this, it is possible to use multiprocessing and decrease RAM usage. Just run the programme in different shells or on different machines and copy all resulting index folders to the same main directory. The tool “xapian-compact” summarises all generated directories to one full text index:

  > - http://xapian.org/docs/admin\_notes.html#merging-databases
  > - xapian-compact -m <all input directories to be compressed, separated by space> <name of outcoming folder with complete database>
  > - Using Ubuntu, this tool might have to be installed additionally with “sudo apt install xapian-tools”.

# 6 Examples for Using Full Text Search and Selecting Data from PostgreSQL

## 6.1 Xapian

- Use the following scripts to work with the functions OR, AND, NEAR, ADJ, NOT, and phrase search in Xapian and have a look at the HTML output files. As the number of PubMed-IDs increases continuously, the resulting numbers in this documentation can be seen as a reference point for the given query “pancreatic cancer”. Having a look at these scripts as well as “RunXapian.py” can be useful to build your own modified queries. There is also a small note in “full\_text\_index/xapian/readme.txt”.

  > - “python search\_title.py” shows that only a few lines of code are required to search only publication titles. This can be important as searching especially in publication titles puts more emphasis on the queried synonyms.
  >
  >   > - While “RunXapian.py” searches only the exact phrase “pancreatic cancer”, “search\_title.py” searches for the stem “pancreat” and also finds the word “pancreatitis”.
  > - The search terms in the scripts described in this subsection are hard-coded and have to be changed manually by the user.
  >
  >   > - It generates “Xapian\_query\_results.html” which shows the first 1000 of 18085 titles. Like this, many associated words are shown, e.g. “pancreatic ductal adenocarcinoma”, “pancreatic juice”, or “pancreatic diseases”.
  > - To further specify your search, you can query titles containing “pancreatic cancer” and the drug “erlotinib” with “python search\_near\_title.py”.
  >
  >   > - This generates 38 results in “Xapian\_query\_results\_NEAR.html”.
  >   > - In this case “NEAR/5” is used as a Xapian function. In this case, a maximum of 4 words is allowed to be between the two search terms.
  >   > - An alternative would be the query with “ADJ/5”, which reduces the number of 38 hits to 4 hits, because with this function, the order of search terms is fixed.
  >   > - Here, the exact search is performed, again.
  > - As it was done in “RunXapian.py” different index fields can be searched.
  >
  >   > - “python search\_title\_or\_text.py” searches documents in which the drug “R115777” occurs in the title or the text.
  >   > - As shown in “counts\_results.csv”, only 10 hits can be found. The matching titles and abstracts can be seen in “Xapian\_query\_results\_OR.html”.
  > - The script “python search\_not\_title\_or\_text.py” specifies the query to documents not containing the terms “colon”, “lung”, or “ovarian”, but the word “pancreatic”.
  >
  >   > - This reduces the number of results to 9 hits, as no publications are considered that contain these other types of cancer.
  >   > - The result is shown in “Xapian\_query\_results\_NOT.html”.
  > - In this way, different search queries can be combined with a few lines of code.

## 6.2 PostgreSQL

- Type in these SQL queries in PGAdmin3 or in the PostgreSQL shell to get familiar with the schema “pubmed”:

  > - Find all substances related to pancreatic cancer, pancreatitis, etc.
  >
  >   > - select \* from pubmed.tbl\_chemical where lower(name\_of\_substance) LIKE 'pancreati%'; -- 180 lines
  > - Find all MeSH terms with the substring “ancreat” and prefixes as well as suffixes.
  >
  >   > - select distinct on (descriptor\_name) \* from pubmed.tbl\_mesh\_heading where lower(descriptor\_name) LIKE '%ancreat%'; -- 29 lines
  > - What is the number of published titles in our database?
  >
  >   > - select count(\*) from pubmed.tbl\_medline\_citation; -- 23258
  > - How many publications contain an abstract?
  >
  >   > - select count(\*) from pubmed.tbl\_abstract; -- 21387
  > - Show me all different journals and abbreviations referring to our topic.
  >
  >   > - select distinct on (title, iso\_abbreviation) title, iso\_abbreviation from pubmed.tbl\_journal; -- 2209 lines
  > - What is the number of publications since 1990?
  >
  >   > - select count(\*) from pubmed.tbl\_journal where pub\_date\_year <=2000 and pub\_date\_year >=1990; -- between 1990 and 2000: 3736 publications
  >   > - select count(\*) from pubmed.tbl\_journal where pub\_date\_year <=2010 and pub\_date\_year >2000; -- after 2000 until 2010: 9497 publications
  >   > - select count(\*) from pubmed.tbl\_journal where pub\_date\_year >2010; -- after 2010: 8461 publications
  > - What is the number of publications in USA referring to our topic?
  >
  >   > - select count(\*) from pubmed.tbl\_medline\_journal\_info where lower(country) = 'united states'; -- 10010 publications
  > - Take one of the first publications for the query “pancreatic cancer” in the browser on NCBI and check whether this author has other publications, e.g. Bobustuc et al., 2015.
  >
  >   > - select count (\*) from pubmed.tbl\_author where last\_name = 'Bobustuc'; -- 2

## 6.3 PostgreSQL and Xapian

- The results of this subsection can be found in “full\_text\_index/results/results\_from\_documentation/”.
- Try “python find\_authors.py” to see an example for processing a PostgreSQL query in Python. Use “python find\_authors.py -f <output\_filename> -d <name\_of\_database>” to specify the name of the output file and the database to connect to. “python find\_authors.py -h” shows all adjustable parameters.

  > - Considering the output file “results/authors.csv”, Ralph H. Hruban has published the most articles with a number of 258 PubMed-IDs.
  >
  >   > - The other authors and their number of publications can be found in descending order.
  > - You can check the amount of publications from similiarly written author names in PGAdmin3 and then Helmut Friess is shown as the one with the most publications:
  >
  >   > - select distinct on(fk\_pmid) \* from pubmed.tbl\_author where last\_name = 'Friess' and (fore\_name = 'H' or fore\_name = 'Helmut') order by fk\_pmid; -- 375
  >   > - select distinct on(fk\_pmid) \* from pubmed.tbl\_author where last\_name = 'Büchler' and (fore\_name = 'Markus W' or fore\_name = 'M W') order by fk\_pmid; -- 310
  >   > - select distinct on(fk\_pmid) \* from pubmed.tbl\_author where last\_name = 'Hruban' and fore\_name = 'Ralph H'; -- 258
  > - It is possible that an author name exists twice although different persons are meant. This is not considered here.
  > - There are examples in which you can only find a collective name:
  >
  >   > - select \* from pubmed.tbl\_author where last\_name is NULL and fore\_name is NULL; -- 273
- Based on this, it is possible to consider whether the author Helmut Friess has published something containing the query terms from the list in “synonyms/pancreatic\_cancer.txt”:

  > - Type in “python find\_topics.py”. You can try “python find\_topics.py -h”, to see which parameters can be varied, e.g. if your input filename is not “pmids\_results.csv” or if you want to specify your output filename, which default is “pmids\_results\_from\_author.csv”.
  >
  >   > - 127 publications were found for the given list of synonyms and this author.
  >   > - The main research topic seems to be pancreatic ductal adenocarcinoma. This result can be compared with the outputs using other author names (hard coded in “find\_topics.py”) and running “find\_topics.py” with another filename, again.
- Next steps can be to select the abstracts that were identified with Xapian from PostgreSQL and to apply software for named entity recognition (section “Examples for Using BioC and PubTator”) or to visualise data (next section). There are many possibilities to develop customised pipelines, e.g. selecting sentences, applying part-of-speech tagging, and train machine learning models to extract semantic relationships.

# 7 Examples for Generating Plots

- This section covers the generation of word clouds and pie charts, for which special software packages are needed.

## 7.1 Word Cloud

> - The word clouds generated here are based on the modified Xapian full text version searching only PubMed titles and abstract texts. Therefore, the files “RunXapian.py” and “SynonymParser.py” as well as the folder “synonyms” need to be copied from the folder “full\_text\_index” to the folder “full\_text\_index\_title\_text”. The directories “xapian” and “results” have to be created, too. Afterwards, the command “python RunXapian.py -x” can be used, again. The numbers described in the last sections can differ slightly from the results generated here. The command “python summary.py” also has to executed.
> - At first, the list of the 50 most frequently occurring words that were generated with “python summary.py” needs to be extracted in logarithmic scale to visualise the search terms appropriately. In the directory “PubMedPortable/plots/word\_cloud”, run the script “get\_search\_terms\_log.py” to get the output file “counts\_search\_terms\_log.csv”. The highest frequency is shown by the small molecule gemcitabine. The parameter “-h” shows available parameters.
> - The second step in this example is to find the 50 most frequently co-occurring words in texts that contain the search term gemcitabine. This can be done by running the command “python generate\_surrounding\_words\_log.py”. The stop word list that is used by this script was referenced by Hettne et al. [A dictionary to identify small molecules and drugs in free text. Bioinformatics. 2009 Nov 15;25(22):2983-91. doi: 10.1093/bioinformatics/btp535.]. It is provided in the folder “blacklist”. Have a look at the links given in “stop\_words.txt”. These stop words were used to filter out terms with a very high frequency that have no substantial meaning for the content analysed. The numbers in the ouput file “counts\_surrounding\_words\_log.csv” are given in logarithmic scale, too.
> - The search term “Gemcitabine” is hard-coded and needs to be changed directly in the script.
> - The plot can be genrated with the package “PyTagCloud”. Please, follow the installation instructions on this GitHub page:
>
>   > - https://github.com/atizo/PyTagCloud
> - For the first plot given here, use the command “python create\_word\_cloud.py -i counts\_search\_terms\_log.csv -o cloud\_search\_terms.png”.
>
> - For the next figure, run “python create\_word\_cloud.py -i counts\_surrounding\_words\_log.csv -o cloud\_surrounding\_words.png”.
> - The word clouds will look different every time the script is used.
> - The figure area can be enlarged by changing the value of the parameter “size” in the function “create\_tag\_image()”.

## 7.2 Pie Chart

> - In this subsection, the library “matplotlib” is needed to generate a pie chart.
>
>   > - In Ubuntu, this library can be installed with the command “sudo apt-get install python-matplotlib”.
>   > - In Fedora 22, the command “dnf install python-matplotlib” can be used and in case of older Fedora versions, the command “yum install python-matplotlib”.
> - By running “python pie\_chart\_countries.py”, the picture “pie\_chart\_countries\_publications.png” is produced from the input file “countries\_pancreatic\_cancer.csv”.
> - To get the CSV file, you need to connect to your database, e.g. with “psql -h localhost -d pancreatic\_cancer\_db -U parser” and type in “\COPY (SELECT fk\_pmid, LOWER(country) FROM pubmed.tbl\_medline\_journal\_info WHERE country IS NOT NULL ORDER BY country ASC) TO 'countries\_pancreatic\_cancer.csv' DELIMITER ','”.
> - The script calculates the percentages of country names in which the journals given in the PostgreSQL database are published. Fractions below 2 % are summarised to “Others”.
> - The plot was inspired by an example given in the Matplotlib documentation (http://matplotlib.org/examples/pie\_and\_polar\_charts/pie\_demo\_features.html).

## 7.3 Bar Chart

> - In this subsection, the library “matplotlib” is needed, too.
> - Three timelines for the publications of the genes KRAS, BRCA2, and CDKN2A are shown in one bar chart.
> - Running “create\_bar\_chart.py -p” generates the figure “KRAS\_BRCA2\_CDKN2A\_pubmed.png”. The year 2015 cannot be considered as a complete year. Therefore, it is removed by this script before plotting.
> - The CSV files processed by this script can be downloaded from PubMed by clicking on the bar chart appearing on http://www.ncbi.nlm.nih.gov/pubmed after entering the query (15th June 2015). The title lines in these CSV files were removed manually.
> - All CSV files used in this subsection are written in comma-separated format.
>
> - Running “python get\_years.py” generates the same kind of CSV files as provided by the browser search, but it uses the pancreatic cancer data set from this documentation by sending a query to the PubMedPortable PostgreSQL database.
>
>   > - Running this script with default parameters selects the user-based Xapian folder “full\_text\_index\_title\_text”, but it can also be used with the results file in this documentation to reproduce the plot shown here:
>   > - python get\_years.py -x ../../full\_text\_index/results/results\_from\_documentation/ -p results.csv
> - Based on this, “create\_bar\_chart.py” without the parameter “-p” generates the bar chart “KRAS\_BRCA2\_CDKN2A.png”.
>
> - The slopes of the BRCA2 and CDKN2A timelines are rather low compared to KRAS, but start earlier in both plots. The timeline of the gene KRAS shows an exponential growth. One reason for this is its role in the regulation of cell proliferation [Small molecule inhibition of the KRAS-PDEδ interaction impairs oncogenic KRAS signalling. Zimmermann et al. Nature. 2013 May 30;497(7451):638-42. doi: 10.1038/nature12205. Epub 2013 May 22.].
> - The review on OMIM mentioned in section 5 (http://omim.org/entry/260350?search=%22pancreatic%20cancer%22) provides more information with references showing why and how specific these genes are related to pancreatic cancer.

# 8 Examples for Using BioC and PubTator

- Follow the installation instructions on this GitHub page:

  > - https://github.com/2mh/PyBioC.
- Copy the project folder “PyBioC/src/bioc” into your folder “PubMedPortable/BioC\_export”. This PyBioC directory is needed for the script “add\_BioC\_annotation.py”, because it contains the Python source code for the BioC interface.
- The idea of BioC is to use a standardised XML format that can be shared by the community to add annotations to scientific texts. Therefore, these documents can be exchanged and modified by anybody who uses the BioC interface.
- The BioC XML format was introduced at BioCreative (http://www.biocreative.org/events/BCBioCuration2014/biocreative-text-mining-worksh) and also used at BioNLP (http://2013.bionlp-st.org/supporting-resources). The BioC project homepage contains several related software packages (http://bioc.sourceforge.net).
- The file “pmid\_list.txt” contains 21 PubMed-IDs that were taken from “PubMedPortable/data/pubmed\_result.txt”. It is used as default by the script “write\_BioC\_XML.py”.

  > - The user can store his own list of PubMed-IDs in “pmid\_list.txt” or create a new file. This user-provided list of PubMed-IDs can be loaded with the parameter “-i”.
  > - New PubMed-IDs can be selected from the PubMedPortable PostgreSQL tables, e.g. pubmed.tbl\_abstract, pubmed.tbl\_medline\_citation, or pubmed.tbl\_mesh\_heading.
- This script also uses the file “BioC.dtd”, which defines the structure of the XML file (taken from https://github.com/2mh/PyBioC). Additionally, the file “Explanation.key” describes the semantics used for the annotations. In this example, MeSH terms are added as annotation XML elements to the basic BioC XML structure.
- For the list of PubMed-IDs, the command “python write\_BioC\_XML.py” generates the BioC XML file “text\_BioC.xml”.
- If you run “python add\_BioC\_annotation.py” with default values, it adds the MeSH terms provided by the PostgreSQL database to the file “text\_BioC.xml”. It does not change the basic structure, but it writes annotation XML elements for the text elements given. The output file is called “annotated\_text\_BioC.xml”. This is an example for an XML file that did not contain annotation elements before.
- You can use the same script for adding the MeSH annotation elements to an XML document that already contains other annotation elements as shown in the next example.
- PubTator can be used as a webservice in several ways. There is a website highlighting entities like genes/proteins, chemical compounds, diseases, mutations, and species. This website can be used for text curation:

  > - http://www.ncbi.nlm.nih.gov/CBBresearch/Lu/Demo/PubTator
- There is also a RESTful API:

  > - http://www.ncbi.nlm.nih.gov/CBBresearch/Lu/Demo/tmTools/curl.html
- How to use this API is shown by the following command executed via command-line:

  > curl -H “content-type:application/json” http://www.ncbi.nlm.nih.gov/CBBresearch/Lu/Demo/RESTful/tmTool.cgi/Disease/1000475,1006519,1010707/BioC/ > text\_PubTator.xml
  >
  > - All output files in this section refer to these three PubMed-IDs. The maximum number of PubMed-IDs to send to PubTator in this case was 21. This is the reason why “pmid\_list.txt” contains exactly 21 PubMed-IDs.
- The script “call\_PubTator.py” wraps this command with the Python module “subprocess” and downloads PubMed BioC XML annotated abstracts from PubTator.

  > - It contains the parameter “-t” (trigger) that selects the type of entity to be tagged (default: Disease) for a list of PubMed-IDs (default: pmid\_list.txt).
  > - All parameters of this script can be shown with “python call\_PubTator.py -h”.
  > - The default output file “text\_PubTator.xml” also shows the MeSH IDs for the extracted diseases, e.g. “pancreatic carcinoma”, the first one in the example file:
  >
  >   > - http://www.nlm.nih.gov/cgi/mesh/2011/MB\_cgi?field=uid&term=D010190
  > - PubTator returns two types of infon elements. Therefore, the line “<!ELEMENT annotation ( infon\*, location\*, text ) >” had to be changed to “<!ELEMENT annotation ( infon\*, location\*, text, infon\* ) >”.
- If you want to add MeSH term annotations from the PostgreSQL to the file “text\_PubTator.xml”, you can run the command “python add\_BioC\_annotation.py -i text\_PubTator.xml -o annotated\_text\_PubTator.xml”.

  > - MeSH terms refer to several types of entities. In this case, some of the MeSH terms will show duplicate disease annotation elements.
- All these entities can also be tagged in BioC XML format from plain text input via the single software packages referenced here below “Quick Links”:

  > - http://www.ncbi.nlm.nih.gov/CBBresearch/Lu/Demo/tmTools
- They are described in the following PDF file as well as other software packages in chapter “TRACK 1 (BioC: Interoperability)”:

  > - http://www.biocreative.org/media/store/files/2013/ProceedingsBioCreativeIV\\_vol1\\_.pdf
  > - There are also other webservices included as well as BioC natural language preprocessing pipelines in C++ and Java (http://bioc.sourceforge.net).
- PubTator can be used to completly extract genes, diseases, and chemicals from the pancreatic cancer data set. In the case of diseases and chemicals, there are not always identifiers provided for the recognised synonyms. The following commands lead to a new word cloud based on the 150 most frequently occurring entities:

  > - Gene and protein NER: python call\_PubTator.py -i pubmed\_result\_complete.txt -o gene\_complete.csv -t Gene -f PubTator
  > - Disease NER: python call\_PubTator.py -i pubmed\_result\_complete.txt -o disease\_complete.csv -t Disease -f PubTator
  > - Chemical NER: python call\_PubTator.py -i pubmed\_result\_complete.txt -o chemical\_complete.csv -t Chemical -f PubTator
  > - File concatenation: cat gene\_complete.csv disease\_complete.csv chemical\_complete.csv > entities\_complete.csv
  > - Get PubMed IDs, synonyms, and identifieres: python results\_PubTator\_format.py -i entities\_complete.csv -o entities\_formatted\_identifiers.csv
  > - Count entities, summarised by their identifiers: python unify.py -i entities\_formatted\_identifiers.csv -o entities\_formatted\_identifiers\_unified.csv
  > - Generate logarithmic values (first 150 entities): python get\_search\_terms\_log.py -x ../../BioC\_export/results\_from\_documentation -i entities\_formatted\_identifiers\_unified.csv -o counts\_entities\_identifiers\_log.csv
  > - Create word cloud: python create\_word\_cloud.py -i counts\_entities\_identifiers\_log.csv -o cloud\_entities\_identifiers.png
  > - This example is based on selecting one synonym per identifier. The script “results\_PubTator\_format.py” can be used with the parameter “-s” to extend the selection to all synonyms without using the identifiers. In this case, the step of using the script “unify.py” needs to be replaced with the script “summary.py” in the Xapian folder “full\_text\_index”.
- The bar chart shown with manually selected search terms can also be produced with the automatically identified entities from PubTator introduced in this section:

  > - python get\_years.py -x ../../BioC\_export/results\_from\_documentation/ -p entities\_formatted\_identifiers.csv -t Entrez\_GeneID/search\_terms\_KRAS.txt -o KRAS
  > - python get\_years.py -x ../../BioC\_export/results\_from\_documentation/ -p entities\_formatted\_identifiers.csv -t Entrez\_GeneID/search\_terms\_BRCA2.txt -o BRCA2
  > - python get\_years.py -x ../../BioC\_export/results\_from\_documentation/ -p entities\_formatted\_identifiers.csv -t Entrez\_GeneID/search\_terms\_CDKN2A.txt -o CDKN2A
  > - python merge.py
  > - python create\_bar\_chart.py
  > - Based on the search for a larger vocabulary from PubTator using Entrez GeneID numbers, CDKN2A shows more hits than BRCA2 and the identified numbers of abstracts are generally higher.
- The same steps as performed with PubTator can be performed with other tools, too.

  > - The disease annotation step can be replaced by the stand-alone application DNorm from the tmBioC package (http://www.ncbi.nlm.nih.gov/CBBresearch/Lu/Demo/tmTools/ - link to DNorm):
  >
  >   > - Write BioC document from pancreatic cancer data set (BioC directory): “python write\_BioC\_XML.py -i pubmed\_result.txt -o pancreatic\_cancer\_BioC.xml”
  >   > - Add DNorm annotatons (in download directory): ./RunDNorm\_BioC.sh config/banner\_NCBIDisease\_TEST.xml data/CTD\_diseases.tsv output/simmatrix\_NCBIDisease\_e4.bin pancreatic\_cancer\_BioC.xml pancreatic\_cancer\_BioC\_DNorm.xml
  >   > - This command can be used to create the “file pancreatic\_cancer\_BioC\_DNorm.xml” (not uploaded).
  >   > - The script “read\_BioC\_annotations.py” shows the basic commands how to iterate over MeSH term annotations in BioC format from the example mentioned earlier, using PubMedPortable and PubTator.
  >   > - The script “BioC\_to\_CSV.py” is based on the code in “read\_BioC\_annotations.py” and extracts the DNorm annotations in “file pancreatic\_cancer\_BioC\_DNorm.xml” to a CSV file “DNorm\_formatted.csv (not uploaded). The script needs the DNorm DTD file (in the DNorm download directory). Copy it to you execution folder and rename it to”BioC\_DNorm.dtd". If this file causes an error in the PyBioC API, replace the raise command in bioc/bioc\_reader.py by a print command.
  > - Genes and proteins can be annotated with GeneTUKit, a software for gene normalisation which was ranked among the best-performing tools in the BioCreative III challenge in 2010.
  > - Unfortunately, the source code is not available, but there is a GitHub repository wrapping PubMedPortable articles into a pseudo XML format used by the software (https://github.com/ElhamAbbasian/GeneTUKit-Pipeline).
  > - Using the list of PubMed IDs from the PubMedPortable documentation and following the first three steps in the GeneTUKit pipeline generates a file pmid\_geneid\_syn.csv.
  > - For the output format used to generate the word cloud, the orginal line to write the output in the script filter\_out\_genetukit\_output.py can be changed to ‘outfile.write(pmid + “t” + temp[1].split(“|”)[0] + “t” + temp[0] + “n”)’. Multiple synonyms with the same Entrez Gene-ID number are separeted with a pipe (“|”) and only the first synonym is needed for the task here. Furthermore, the order from the file name “PubMed-ID-GeneID-Synonym” is changed to “PubMed-ID-Synonym-GeneID” by exchanging the elements temp[0] and temp[1].
  > - After file concatenation (single files not uploaded: cat GeneTUKit\_formatted.csv DNorm\_formatted.csv chemical\_formatted.csv > entities\_complete\_3tools.csv), the steps to generate the word cloud can be executed as already described in the PubTator example.
  >
  >   > - python unify.py -i entities\_complete\_3tools.csv -o entities\_complete\_3tools\_unified.csv
  >   > - get\_search\_terms\_log.py -x ../../BioC\_export/results\_from\_documentation -i entities\_complete\_3tools\_unified.csv -o counts\_entities\_identifiers\_log\_3tools.csv
  >   > - create\_word\_cloud.py -i counts\_entities\_identifiers\_log\_3tools.csv -o cloud\_3tools.png
  > - The same is possible for the bar chart example.
  >
  >   > - The Entrez GeneID numbers were extracted from the file GeneTUKit\_formatted.csv with the script get\_search\_term\_identifiers.py (KRAS gene example hard-coded).
  >   > - This has to be done with the other two genes CDKN2A and BRCA2, too. The steps “python merge.py” and “python create\_bar\_chart.py” lead to the new bar chart “KRAS\_CDKN2A\_BRCA2.png”, manually renamed to “KRAS\_CDKN2A\_BRCA2\_3tools.png” to be distinguishable from the PubTator example.
  >   > - This approach leads to a higher number of publications for each gene, but shows basically the same tendencies as in the PubTator example.
  > - The example of using PubTator, DNorm, and GeneTUKit illustrates, that the infrastructure of PubMedPortable can be easily extended to combine different data formats (PubTator, BioC, and pseudo XML format), being independent from a Web service, but making use of it, if desired.

# 9 Indexing of PMC Articles

## 9.1 Introduction

- The page ftp://ftp.ncbi.nlm.nih.gov/pub/pmc/ contains all downloadable files used in this section.
- Explanations for the PMC FTP service can be found here:

  > http://www.ncbi.nlm.nih.gov/pmc/tools/ftp/
- The four files articles.txt.0-9A-B.tar.gz, articles.txt.C-H.tar.gz, articles.txt.I-N.tar.gz, and articles.txt.O-Z.tar.gz contain all available PMC full text articles in plain text format.
- In more than half of all PMC articles, the authors did not allow a download of their article in text format. This is also true for the available XML downloads and the ID Converter API (http://www.ncbi.nlm.nih.gov/pmc/tools/id-converter-api/).
- This section considers the insertion of a PMC-PubMed-ID mapping of all available IDs in PostgreSQL, the indexing of all downloaded PMC articles in Xapian, and the insertion of the articles without further formatting in PostgreSQL.
- The approach can be extended with a PMC XML parser, e.g. this one:

  > - https://sourceforge.net/projects/pmcparser
- If the user does not need the texts in PostgreSQL, they can also be stored directly in a text field in the Xapian index.
- The file file\_list.txt contains a mapping of the downloaded article names to PMC IDs (http://www.ncbi.nlm.nih.gov/pmc/tools/ftp/), which is also stored in PostgreSQL.

## 9.2 Get PMC Files

- Create a directory files in your PMC folder in the GitHub project and download the 4 PMC article archives, if you want to index all of them as shown here for the first gzip file:

  > - wget ftp://ftp.ncbi.nlm.nih.gov/pub/pmc/articles.txt.0-9A-B.tar.gz
- Unzip the files and remove the source files:

  > - gunzip articles.txt.0-9A-B.tar.gz
  > - tar -xf articles.txt.0-9A-B.tar
  > - rm articles.txt.0-9A-B.tar
- Each gzip file will have an approximate size of 4.2 GB [2015-04-22]
- Download the mapping of PMC IDs to PubMed IDs in your PMC folder:

  > - wget ftp://ftp.ncbi.nlm.nih.gov/pub/pmc/PMC-ids.csv.gz
  > - gunzip PMC-ids.csv.gz

## 9.3 Create Tables and Xapian Index

- Create a table tbl\_pmcid\_name\_pmid in your PostgreSQL schema public:

  > - psql -h localhost -d pancreatic\_cancer\_db -U parser -f create\_pmcid\_pmid\_table.sql
- Insert PMC ID mapping in your PostgreSQL database with PubMed IDs, if contained (not all PMC IDs will contain a PubMed ID mapping - nevertheless, it can be checked whether the PubMed ID is referenced to a PMC ID with this table):

  > - python insert\_PMC\_ID\_PubMed\_ID\_mapping.py
- If you want to count the number of uploaded PMC IDs, use the following command, e.g. in PGAdmin:

  > - select count(\*) from tbl\_pmcid\_pmid;
- Insert PMC ID mapping from file\_list.txt, which also contains the file names from the downloaded archives:

  > - psql -h localhost -d pancreatic\_cancer\_db -U parser -f create\_pmcid\_name\_pmid\_table.sql
  > - python insert\_PMC\_ID\_Name\_PubMed\_ID\_mapping.py
- Check the total number of downloaded text files from the archives. This number will be much smaller than the number of PMC IDs in tbl\_pmicid\_pmid:

  > - select count(\*) from tbl\_pmcid\_name\_pmid;
- Build the Xapian index - this might take a few hours in total, depending on the following options. Create a folder xapian first:

  > - mkdir xapian
  > - Set the boolean flag of the variable use\_psql to True in line 35 in index.py (default is True) if you want to store your PMC texts in the PostgreSQL table tbl\_pmcid\_text, otherwise an extra Xapian data field will be used to save the file content, e.g. to read it after receiving search results.
  > - If you want to use the PostgreSQL database, create the table tbl\_pmcid\_text first:
  >
  >   > - psql -h localhost -d pancreatic\_cancer\_db -U parser -f create\_pmcid\_text\_table.sql
  >   > - The indexing process for the first of four files took over an hour with one CPU core (2,83 GHz and 8 GB RAM). Setting use\_psql to True or False resulted in a similar runtime.
  > - python index.py
- Before the index can be used completely, it has to be merged with the compact-tool already mentioned earlier in this documentation. The following command will generate a folder xapian\_PMC\_complete in your PMC directory:

  > - python generate\_xapian\_compact\_command.py
  > - It is also possible to include only some selected journals in the search by using the IDs generated during the indexing process (ids.txt).
- To search in the index with showing identified texts, run the following script with the parameter use\_psql set to True (default case, line 24) and verbose set to True (default False, line 22). Create a result directory first. Similar to the already described search procedure in the Xapian chapter of this documentation, a list of synonyms can be used (synonyms/synonyms.txt):

  > - search.py
  > - This script rather serves as a guidline how to get the search results and should be adapted to the methods already described.
- The scripts which generated the results described in the other chapters can be adapted to be used with the data processed in this section.

# 10 Named Entity Recognition Tools

- The following table shows named entity recognition tools. Many stand-alone and web service applications are available. Therefore, the overview cannot be considered as complete.
- There will be new publications from every BioCreative challenge (http://www.biocreative.org).
- The first 5 applications can be used easily as a web service with the PubMedPortable script call\_PubTator.py (Wei et al.).
- DNORM was used as a stand-alone tool in the wiki section “Examples for Using BioC and PubTator”.
- TaggerOne can be trained to be used highlight any entity type. The publication shows benchmarked results for chemicals and diseases.

| Tool Name | Entities | Availability | Authors |
| --- | --- | --- | --- |
| GNormPlus | genes/proteins | stand-alone tool and web service | Wei et al., 2015 |
| DNorm | diseases/species/taxonomy | stand-alone tool and web service | Leaman et al., 2013 |
| tmChem | drugs/chemicals | stand-alone tool and web service | Leaman et al., 2014 |
| tmVar | mutations/diseases | stand-alone tool and web service | Wei et al., 2013 |
| SR4GN | species/genes | stand-alone tool and web service | Wei et al., 2012 |
| GNAT | genes/proteins | stand-alone tool and web service | Hakenberg, J. et al. 2010 |
| LINNAEUS | species/taxonomy | stand-alone tool and web service | Gerner et al., 2010 |
| TaxonGrab | species/taxonomy | stand-alone tool | Moritz et al., 2005\_ |
| Whatizit | species/taxonomy | web service | Rebholz-Schuhmann et al., 2008 |
| MutationFinder(BioNLP) | mutations | standalone tool | Caporaso et al., 2007 |
| CTD | genes/proteins/chemicals/protein interactions | web service | Wiegers et al., 2014 |
| Reflect | proteins/chemicals | stand-alone tool and web service | Pafilis et al., 2009 |
| TaggerOne | chemicals/diseases | web service | Leanman et al., 2016 |
| CD-REST | chemicals/diseases | web service | Xu et al., 2016 |

# 11 Lucene as an Alternative to Xapian

- There is no contradiction in using Xapian or Lucene. We wanted to build a full text index with only a few lines of code in Python, based on an easy installation. Therefore, we chose Xapian.
- Our workflows and use cases were generated with Python code. Lucene can also be called in Python via PyLucene.
- To illustrate this modularity, we created a minimalistic indexing and searching example. The examples were inspired by the following sources:

  > - IndexFiles.py, SearchFiles.py, and FacetExample.py in http://svn.apache.org/viewvc/lucene/pylucene/trunk/samples
  > - http://graus.co/blog/pylucene-4-0-in-60-seconds-tutorial
  > - http://blog.intelligencecomputing.io/tags/pylucene
- Of course, you are free to use Lucene completely in Java - this is just a basic tutorial to simplify the first steps in indexing and searching with PyLucene.

## 11.1 Installation

- The installation steps on the official PyLucene page (http://lucene.apache.org/pylucene/install.html) did not work straigt forward in Ubuntu 16 LTS.
- This section is considered as an alternative to Xapian. Therefore, the installation steps are not part of the general introduction to installation requirements of PubMedPortable.
- Install the following packages using the official Ubuntu sources (“apt-get install” or “sudo synaptic”).

  > - jcc
  > - python-all-dev
- The official PyLucene instruction state that you should use the JCC SVN version (http://lucene.apache.org/pylucene/jcc/install.html).
- If the Ubuntu default package “jcc” does not work, try the following steps (as described in the official documentation).

  > - svn jcc
  > - pushd jcc (changes into your jcc directory)
  > - edit your java path if your receive an error (e.g. ‘linux2’: ‘/usr/lib/jvm/java-8-openjdk-amd64’,)
  > - python setup.py build
  > - sudo python setup.py install
  > - popd
- Download PyLucene, e.g. from http://apache.lauf-forum.at/lucene/pylucene (version 4.9.0.0 used in this section).
- Change into unzipped directory and edit Makefile by deleting the comment characters in lines 93-97:

  > PREFIX\_PYTHON=/usr
  >
  > ANT=JAVA\_HOME=/usr/lib/jvm/java-8-openjdk-amd64 /usr/bin/ant
  >
  > PYTHON=$(PREFIX\_PYTHON)/bin/python
  >
  > JCC=$(PYTHON) -m jcc –shared
  >
  > NUM\_FILES=8
- Execute the following commands in your terminal (in the PyLucene folder):

  > - make
  > - make test (any errors?)
  > - sudo make install
- You should be able to do “import lucene” now, e.g. in IPython.

## 11.2 Usage

- The PubMedPortable examples index.py and search.py can be modified to be included in the script “PubMedXapian.py” using Xapian with the functions “buildIndexWithArticles(articles)” and “findPMIDsWithSynonyms(synonyms)”.
- The script index.py creates a Lucene folder “lucene\_index.Index” and adds two documents with the fields “Title” and “Abstract”.
- If your installation worked fine, you will see the output “Indexed 2 documents.”.
- The option “Field.Store.YES” can be considered analogously to the Xapian option “xappy.FieldActions.STORE\_CONTENT” in PubMedXapian.py.

  > - Enabling this option means, that you can inspect the source of your matching document directly.
  > - This increases your index size. Alternatively, you can query the sources from your PostgreSQL database.
- The script search.py shows two queries. The first query matches both documents, because it searches with “OR” in the fields “Title” and “Abstract”.
- The second query in search.py matches only the first document because of the condition “AND”.
- You should see the following output:

  > Searching for ‘text’ with OR two match both documents:
  >
  > 2 total matching documents.
  >
  > title: text of title1 , abstract: abstract1 has many words, e.g. hellow world can be the text
  >
  > title: title2 , abstract: text of abstract2
  >
  > Searching for ‘text’ with OR two match only the first document:
  >
  > 1 total matching documents.
  >
  > title: text of title1 , abstract: abstract1 has many words, e.g. hellow world can be the text
- The usage of PyLucene seems to be simple as well, but the installation can be more difficult. Considering advanced tasks in PyLucene, Java knowledge is clearly an advantage to find and make use of the range of packages and functions.

# 12 Contact

- Please, write an e-mail, if you have questions, feedback, improvements, or new ideas:

  > - kersten.doering at gmail dot com
- If you are interested in related projects, visit our working group’s homepage:

  > - http://www.pharmaceutical-bioinformatics.de

## 12.1 License

- PubMedPortable is published with an ISC license given in “license.txt”.
