# Supplementary material for: PubMedPortable: A Framework for Supporting the Development of Text Mining Applications
Source: PLoS One. 2016 Oct 5;11(10):e0163794. doi: 10.1371/journal.pone.0163794 (PMC5051953; doi:10.1371/journal.pone.0163794)
Supplement: S1 File — (ZIP) [file pone.0163794.s001.zip › PubMedPortable-master/full_text_index/Xapian_query_results.html]

Xapian\_query\_results


| Rank | PubMed-ID | Title (query term highlighted) |
| --- | --- | --- |
| 0 | 7272919 | **Pancreatic** arteriography, transhepatic **pancreatic** venography, and **pancreatic** venous sampling in diagnosis of **pancreatic** cancer. |
| 1 | 22413456 | [**Pancreatic** tumor: progress in diagnosis and treatment. Topics: I. **Pancreatic** carcinoma: 2. Pathogenesis and pathobiology in **pancreatic** cancer.--The molecular mechanisms of carcinogenesis, and invasion and metastasis in **pancreatic** cancer]. |
| 2 | 22413457 | [**Pancreatic** tumor: progress in diagnosis and treatment. Topics: I. **Pancreatic** carcinoma; 3. Differential diagnosis of **pancreatic** cancer]. |
| 3 | 22413458 | [**Pancreatic** tumor: progress in diagnosis and treatment. Topics: I. **Pancreatic** carcinoma; 4. Progress of non-surgical treatment for **pancreatic** cancer]. |
| 4 | 22413460 | [**Pancreatic** tumor: progress in diagnosis and treatment. Topics: I. **Pancreatic** carcinoma; 6. Evidence based clinical practice guidelines for **pancreatic** cancer 2009]. |
| 5 | 4797413 | [Cytodiagnosis of **pancreatic** cancer using the **pancreatic** juice selectively aspirated from the **pancreatic** duct under duodenoscopy]. |
| 6 | 707739 | Endoscopic measurement of **pancreatic** juice secretory flow rates and **pancreatic** secretory pressures after secretin administration in human controls and in patients with acute relapsing **pancreatitis**, chronic **pancreatitis**, and **pancreatic** cancer. |
| 7 | 3531954 | Elevated **pancreatic** oncofetal antigen levels measured by enzyme immunoassay in pure **pancreatic** juice of patients with **pancreatic** cancer. |
| 8 | 2322926 | Induction of multifocal **pancreatic** cancer after inoculation of hamster **pancreatic** cancer cell line (PC-1) into a defined area of homologous **pancreatic** tissue. |
| 9 | 332575 | Detection of bacterial infection of the **pancreatic** ducts in patients with **pancreatitis** and **pancreatic** cancer during endoscopic cannulation of the **pancreatic** duct. |
| 10 | 9613127 | [Specific activation of telomerase in **pancreatic** cancer tissue and preoperative diagnosis of **pancreatic** cancer by telomerase in **pancreatic** juice]. |
| 11 | 7216142 | Diagnosis of **pancreatic** cancer by **pancreatic** oncofetal antigen (poa) in pure **pancreatic** juice. |
| 12 | 25438071 | Incidence of post-ERCP **pancreatitis** from direct **pancreatic** juice collection in hereditary **pancreatitis** and familial **pancreatic** cancer before and after the introduction of prophylactic **pancreatic** stents and rectal diclofenac. |
| 13 | 21467773 | [Endoscopic **pancreatic** stenting was effective in a case of **pancreatic** duct disruption and leakage due to **pancreatic** cancer]. |
| 14 | 7336130 | **Pancreatic** oncofetal antigen in **pancreatic** juices. Partial chemical characterization and diagnostic application of a **pancreatic** cancer-associated antigen. |
| 15 | 1193344 | Combined endoscopic **pancreatic** fluid collection and retrograde pancreatography in the diagnosis of **pancreatic** cancer and chronic **pancreatitis**. |
| 16 | 3290345 | Laboratory tests in the diagnosis of the chronic **pancreatic** diseases. Part 6. Differentiation between chronic **pancreatitis** and **pancreatic** cancer. |
| 17 | 3895424 | [Value of sonographic imaging of the **pancreatic** duct for the diagnosis of chronic **pancreatitis** and **pancreatic** cancer compared to ERCP]. |
| 18 | 1698685 | Immunochemical characterization and quantitative distribution of **pancreatic** stone protein in sera and **pancreatic** secretions in **pancreatic** disorders. |
| 19 | 18362835 | In situ telomerase activity in **pancreatic** juice may discriminate **pancreatic** cancer from other **pancreatic** diseases. |
| 20 | 19568227 | **Pancreatic** neuropathy results in "neural remodeling" and altered **pancreatic** innervation in chronic **pancreatitis** and **pancreatic** cancer. |
| 21 | 21720164 | Neuroendocrine **pancreatic** tumors are risk factors for **pancreatic** fistula after **pancreatic** surgery. |
| 22 | 1744434 | Acute **pancreatitis** and **pancreatic** cancer. Biology, diagnosis, and therapy. Proceedings of the fourth meeting of the International Association of Pancreatology and the third meeting of the International **Pancreatic** Cancer Study Group. August 20-23, 1990, Nagasaki City, Japan. |
| 23 | 19077459 | Proteomic analysis of **pancreatic** ductal adenocarcinoma compared with normal adjacent **pancreatic** tissue and **pancreatic** benign cystadenoma. |
| 24 | 23271395 | Ratio of **pancreatic** duct caliber to width of **pancreatic** gland by endosonography is predictive of **pancreatic** cancer. |
| 25 | 6367596 | Differential distribution of the **pancreatic** cancer-associated antigen (PCAA) and **pancreatic** tissue antigen (PaA) in **pancreatic** and gastrointestinal cancer tissues. |
| 26 | 23086246 | Usefulness of brush cytology combined with **pancreatic** juice cytology in the diagnosis of **pancreatic** cancer: significance of **pancreatic** juice cytology after brushing. |
| 27 | 25705533 | A suspicious **pancreatic** mass in chronic **pancreatitis**: **pancreatic** actinomycosis. |
| 28 | 25392838 | **Pancreatic** fistula and postoperative **pancreatitis** after pancreatoduodenectomy for **pancreatic** cancer. |
| 29 | 2487070 | CT-guided **pancreatic** percutaneous fine-needle biopsy in differential diagnosis between **pancreatic** cancer and chronic **pancreatitis**. |
| 30 | 22696140 | Presence of **pancreatic** intraepithelial neoplasia in the **pancreatic** transection margin does not influence outcome in patients with R0 resected **pancreatic** cancer. |
| 31 | 12540031 | Overview of the 2002 annual scientific conference of the Lustgarten Foundation for **Pancreatic** Cancer Research: "**Pancreatic** cancer: translating discovery to patient care". |
| 32 | 15899236 | [Physiopathology, diagnosis, and treatment of exocrine **pancreatic** insufficiency in patients with **pancreatic** cancer]. |
| 33 | 12693387 | Dilemma in classifications of possible precursors of **pancreatic** cancer involving the main **pancreatic** duct: PanIN or IPMN? |
| 34 | 10615938 | **Pancreatic** resection combined with intraoperative radiation therapy for **pancreatic** cancer. |
| 35 | 12754397 | The Lustgarten Foundation for **Pancreatic** Cancer Research 3rd Annual Scientific Conference: "**Pancreatic** Cancer: From Genes to Treatment" |
| 36 | 10778162 | [**Pancreatic** oncofetal antigen (POA), **pancreatic** cancer-associated antigen (PCAA)]. |
| 37 | 16291404 | Long-term survival of **pancreatic** cancer patient diagnosed by positive telomerase activity of **pancreatic** juice. |
| 38 | 13220862 | [An autopsy case of the **pancreatic** cancer with atypical hyperplasia of epithelium of small **pancreatic** duct]. |
| 39 | 1401832 | Levels of carcinoembryonic antigen and carbohydrate antigen (CA19-9) in pure **pancreatic** juice and sera in a patient with occult **pancreatic** cancer. |
| 40 | 11012299 | Early diagnosis and treatment of **pancreatic** dysplasia in patients with a family history of **pancreatic** cancer. |
| 41 | 14560996 | Vascular **pancreatic** gastric fistula: a complication of colloidal 32P injection for nonresectable **pancreatic** cancer. |
| 42 | 16457226 | [Standard operation for **pancreatic** cancer according to Classification of **Pancreatic** Cancer (5th ed) by Japan Pancreas Society]. |
| 43 | 1654467 | [The usefulness of ERCP and the study of **pancreatic** duct biopsy and brush cytology on the diagnosis of **pancreatic** cancer]. |
| 44 | 11579496 | [A case of small **pancreatic** cancer that was difficult to identify as a **pancreatic** mass]. |
| 45 | 20466283 | Exocrine insufficiency and **pancreatic** enzyme replacement therapy in **pancreatic** cancer. |
| 46 | 19149324 | **Pancreatic** enzyme supplementation in **pancreatic** cancer. |
| 47 | 17698873 | Familial association of benign **pancreatic** hyperenzymaemia and **pancreatic** cancer. |
| 48 | 19260740 | **Pancreatic** cancer. Special issue--insights and controversies in **pancreatic** pathology. |
| 49 | 18024887 | Predicting the risk of **pancreatic** cancer: on CDKN2A mutations in the melanoma-**pancreatic** cancer syndrome in Italy. |
| 50 | 18176038 | Risk factors for **pancreatic** cancer and early diagnosis of **pancreatic** cancer. |
| 51 | 19581759 | Increase in annual number of **pancreatic** head resections does not affect mortality of **pancreatic** cancer in the United Kingdom. |
| 52 | 19639663 | **Pancreatic** cancer: the stealth cancer. **Pancreatic** cancer is hard to detect and highly resistant to chemotherapy; new research reveals possible resolutions. |
| 53 | 18333087 | **Pancreatic** resection for **pancreatic** cancer. |
| 54 | 21160382 | **Pancreatic** cancer as the fourth cancer in a patient with Peutz-Jeghers syndrome and a history of familial **pancreatic** cancer. |
| 55 | 19782297 | **Pancreatic** gout masquerading as **pancreatic** cancer in a heart transplant candidate. |
| 56 | 21265057 | **Pancreatic** cancer: Gemcitabine confirmed as the first-line therapy for **pancreatic** cancer. |
| 57 | 21294478 | Recurrent **pancreatic** adenocarcinoma after **pancreatic** resection. |
| 58 | 18698160 | Genome-wide profiling of methylated promoters in **pancreatic** adenocarcinoma: defining the **pancreatic** cancer [corrected] epigenome. |
| 59 | 21513624 | A novel approach in surgical palliation for unresectable **pancreatic** cancer with untreatable chronic pain: radiofrequency ablation of **pancreatic** mass and celiac plexus. |
| 60 | 18829522 | **Pancreatic** stellate cells and **pancreatic** cancer cells: an unholy alliance. |
| 61 | 2192174 | [Clinical significance of **pancreatic** oncofetal antigen, **pancreatic** cancer associated antigen assay as tumor markers]. |
| 62 | 23964353 | Isolated **pancreatic** tuberculosis mimicking inoperable **pancreatic** cancer: a diagnostic challenge resolved using endoscopic ultrasound-guided fine-needle aspiration. |
| 63 | 21971138 | [New strategy for treatment of patients with **pancreatic** cancer--clinical significance of peptide vaccine for **pancreatic** cancer]. |
| 64 | 2297493 | Identification of the peanut-agglutinin binding **pancreatic** cancer serum marker in **pancreatic** tissue extracts. |
| 65 | 23147663 | **Pancreatic** cancer: New genetic insights into **pancreatic** cancer. |
| 66 | 24189469 | **Pancreatic** cancer: standing on the shoulders of mice, making an iMPACT on **pancreatic** cancer. |
| 67 | 24322903 | **Pancreatic** cancer: Sorafenib: no effect on efficacy of chemotherapy in **pancreatic** cancer. |
| 68 | 2434691 | [Serum **pancreatic** enzymes and enzyme inhibitor as a tumor marker for **pancreatic** cancer]. |
| 69 | 24445614 | **Pancreatic** cancer: **pancreatic** tumour formation and recurrence after radiotherapy are blocked by targeting CD44. |
| 70 | 24514580 | **Pancreatic** cancer: FASCINating insights into the metastatic nature of **pancreatic** cancer. |
| 71 | 22505394 | **Pancreatic** tuberculosis mimicking inoperable **pancreatic** cancer. |
| 72 | 23478386 | **Pancreatic** cancer: FDG-PET is not useful in early **pancreatic** cancer diagnosis. |
| 73 | 22540066 | Non-**pancreatic** cancer tumors in the **pancreatic** region. |
| 74 | 22561444 | Selecting families eligible for **pancreatic** cancer screening: another brick in the wall for the early detection of **pancreatic** ductal adenocarcinoma and its precursors. |
| 75 | 22585132 | **Pancreatic** cancer: USP9X can be used to predict **pancreatic** cancer outcomes. |
| 76 | 23648934 | **Pancreatic** cancer: Radioactive Listeria delivers killer hit to metastatic **pancreatic** cancer. |
| 77 | 22733349 | **Pancreatic** cancer: The role of GM-CSF in **pancreatic** cancer unveiled. |
| 78 | 22733350 | **Pancreatic** cancer: A novel method of imaging **pancreatic** cancer cells and precursors in mice could lead to early diagnosis. |
| 79 | 2658164 | **Pancreatic** resection for **pancreatic** cancer. |
| 80 | 2810909 | [Fundamental and clinical evaluation of IRMA for serum **pancreatic** oncofetal antigen (POA) in **pancreatic** cancer]. |
| 81 | 3130406 | **Pancreatic** sarcoidosis mimicking **pancreatic** cancer. |
| 82 | 3325665 | [Mucin-producing **pancreatic** cancer with unfused main **pancreatic** duct--a case report]. |
| 83 | 3479516 | Levels of carcinoembryonic antigen (CEA) and carbohydrate antigen (CA 19-9) in sera and **pancreatic** juice in the diagnosis of **pancreatic** cancer. |
| 84 | 4482055 | [**Pancreatic** scintigram. 2. **Pancreatic** cancer]. |
| 85 | 4672780 | [Diagnosis of **pancreatic** cancer by selective **pancreatic** angiography]. |
| 86 | 4798450 | [Interpretation of the radiographic findings of the **pancreatic** duct in **pancreatic** cancer]. |
| 87 | 5183254 | [X-ray diagnosis of **pancreatic** diseases by angiography--with special reference to **pancreatic** cancer]. |
| 88 | 5206650 | [X-ray findings of the duodenal bulb **pancreatic** diseases with special reference to clinical experiences in our 2 cases of **pancreatic** cancer]. |
| 89 | 9251127 | **Pancreatic** cancer surgical practice guidelines. **Pancreatic** Cancer Practice Guideline Committee. |
| 90 | 5529260 | [Diagnostic clue to early **pancreatic** cancer-reflux of contrast meal to the **pancreatic** duct. A case report]. |
| 91 | 5622200 | [**Pancreatic** lithiasis and **pancreatic** cancer: 2 cases]. |
| 92 | 5744749 | [**Pancreatic** arteriography; its value in **pancreatic** cancer]. |
| 93 | 588281 | Bile, **pancreatic** cancer, and the activation of **pancreatic** juice. |
| 94 | 6101719 | Enzyme immunoassay of **pancreatic** oncofetal antigen as test for **pancreatic** cancer. |
| 95 | 6115711 | International Meeting on **Pancreatic** Cancer of the National **Pancreatic** Cancer Project. Introduction. |
| 96 | 6115712 | International Meeting on **Pancreatic** Cancer of the National **Pancreatic** Cancer Project. New Orleans, Louisiana, March 10-11, 1980. |
| 97 | 25311477 | **Pancreatic** cancer: Early events in **pancreatic** cancer. |
| 98 | 9436871 | The effect of lithium gamma-linolenate therapy of **pancreatic** cancer on perfusion in liver and **pancreatic** tissues. |
| 99 | 25404108 | Hepatobiliary and **pancreatic**: sigmoidal mesenterial lymph node metastasis from **pancreatic** cancer. |
| 100 | 6843350 | [**Pancreatic** cancer and **pancreatic** calcinosis]. |
| 101 | 6981497 | Ribonuclease C and **pancreatic** secretory proteins in the peripheral circulation before and after pancreatectomy for **pancreatic** cancer. |
| 102 | 7067572 | National **Pancreatic** Cancer Project. Workshop on **pancreatic** tumor markers. |
| 103 | 7220429 | **Pancreatic** oncofoetal antigen in **pancreatic** cancer. |
| 104 | 7272908 | Human **pancreatic** cancer: analysis of proteins contained in **pancreatic** juice by two-dimensional isoelectric focusingsodium dodecyl sulfate gel electrophoresis. |
| 105 | 7393282 | Increased cathepsin B activity in **pancreatic** juice from a patient with **pancreatic** cancer. |
| 106 | 25601668 | **Pancreatic** cancer: From normal to metastases--a whole gamut of **pancreatic** organoids. |
| 107 | 7691373 | Thoracoscopic **pancreatic** denervation for pain control in irresectable **pancreatic** cancer. |
| 108 | 7932811 | Ki-ras mutations in **pancreatic** secretions and aspirates from two patients without **pancreatic** cancer. |
| 109 | 8313100 | Thoracoscopic **pancreatic** denervation for pain control in irresectable **pancreatic** cancer. |
| 110 | 8313101 | Thoracoscopic **pancreatic** denervation for pain control in irresectable **pancreatic** cancer. |
| 111 | 24782785 | Role of **pancreatic** stellate cells in chemoresistance in **pancreatic** cancer. |
| 112 | 24759351 | Isolated **pancreatic** tuberculosis masquerading as **pancreatic** cancer. |
| 113 | 20949480 | Transthyretin, identified by proteomics, is overabundant in **pancreatic** juice from **pancreatic** carcinoma and originates from **pancreatic** islets. |
| 114 | 10872424 | Inheritance of **pancreatic** cancer in **pancreatic** cancer-prone families. |
| 115 | 7349807 | **Pancreatic** cancer: diagnostic value of **pancreatic** function tests. |
| 116 | 1000475 | Carcinoembryonic antigen (CEA) activity in **pancreatic** juice of patients with **pancreatic** carcinoma and **pancreatitis**. |
| 117 | 23458046 | A diagnostic pitfall: **pancreatic** tuberculosis, not **pancreatic** cancer. |
| 118 | 24079797 | Palliation of **pancreatic** ductal obstruction in **pancreatic** cancer. |
| 119 | 21747316 | Galectin-1 secreted by activated stellate cells in **pancreatic** ductal adenocarcinoma stroma promotes proliferation and invasion of **pancreatic** cancer cells: an in vitro study on the microenvironment of **pancreatic** ductal adenocarcinoma. |
| 120 | 3209865 | CA 19-9 in serum and **pancreatic** juice: its role in the differential diagnosis of resectable **pancreatic** cancer from chronic **pancreatitis**. |
| 121 | 705245 | Endoscopic retrograde cholangio-pancreatography in **pancreatic** cancer and chronic **pancreatitis**. Differences in morphologic changes in the **pancreatic** duct and the bile duct. |
| 122 | 11751474 | SMAD4DPC4 and **pancreatic** cancer survival. Commentary re: M. Tascilar et al., The SMAD4 protein and prognosis of **pancreatic** ductal adenocarcinoma. Clin. Cancer Res., 7: 4115-4121, 2001. |
| 123 | 23053424 | Clinical usefulness of repeated **pancreatic** juice cytology via endoscopic naso-**pancreatic** drainage tube in patients with **pancreatic** cancer. |
| 124 | 10907780 | **Pancreatic** adenocarcinoma presenting as sinistral portal hypertension: an unusual presentation of **pancreatic** cancer. |
| 125 | 3062098 | **Pancreatic** function tests in the diagnosis of **pancreatic** cancer. |
| 126 | 21584824 | A focal mass-forming autoimmune **pancreatitis** mimicking **pancreatic** cancer with obstruction of the main **pancreatic** duct. |
| 127 | 20617020 | Beer and its non-alcoholic compounds: role in **pancreatic** exocrine secretion, alcoholic **pancreatitis** and **pancreatic** carcinoma. |
| 128 | 21537863 | Presence of **pancreatic** intraepithelial neoplasia in the **pancreatic** transection margin does not influence outcome in patients with R0 resected **pancreatic** cancer. |
| 129 | 19844153 | [Prevalence of **pancreatic** cancer in diabetics and clinical characteristics of diabetes-associated with **pancreatic** cancer--comparison between diabetes with and without **pancreatic** cancer]. |
| 130 | 2539986 | **Pancreatic** biopsy under visual control in conjunction with laparoscopy for diagnosis of **pancreatic** cancer. |
| 131 | 23846937 | **Pancreatic** cancer stem cells: their role in **pancreatic** cancer patient outcomes and what is future? |
| 132 | 24865539 | Autoimmune **pancreatitis** presenting a short narrowing of main **pancreatic** duct with subsequent progression to diffuse **pancreatic** enlargement over 24 months; natural history of autoimmune **pancreatitis**. |
| 133 | 24555980 | Resection or cryosurgery relates with **pancreatic** tumor type: primary **pancreatic** cancer with previous non-**pancreatic** cancer or secondary metastatic cancer within the pancreas. |
| 134 | 6945001 | The diagnosis of **pancreatic** cancer by **pancreatic** juice cytology. |
| 135 | 21311315 | How fast can **pancreatic** cancer grow? A case of **pancreatic** carcinoma developed within 5 months after a negative examination to the advanced stage with multiple liver and bone metastases. |
| 136 | 3888788 | Enzyme immunoassay of **pancreatic** oncofetal antigen (POA) as a marker of **pancreatic** cancer. |
| 137 | 22109288 | **Pancreatic** surgery for the radiologist, 2011: an illustrated review of classic and newer surgical techniques for **pancreatic** tumor resection. |
| 138 | 20177107 | Slight dilatation of the main **pancreatic** duct and presence of **pancreatic** cysts as predictive signs of **pancreatic** cancer: a prospective study. |
| 139 | 17200706 | The **pancreatic** stellate cell: a star on the rise in **pancreatic** diseases. |
| 140 | 2391873 | [Comparative analysis of **pancreatic** scintigraphy and results of surgical treatment of patients with **pancreatic** cancer]. |
| 141 | 1942900 | [**Pancreatic** cancer. VII. The incidence and characteristics of the metastasis of **pancreatic** adenocarcinoma]. |
| 142 | 23660962 | Role of adjuvant surgery for patients with initially unresectable **pancreatic** cancer with a long-term favorable response to non-surgical anti-cancer treatments: results of a project study for **pancreatic** surgery by the Japanese Society of Hepato-Biliary-**Pancreatic** Surgery. |
| 143 | 17418056 | **Pancreatic** enzyme therapy for **pancreatic** exocrine insufficiency. |
| 144 | 21640917 | **Pancreatic** resection in a large tertiary care community-based hospital: building a successful **pancreatic** surgery program. |
| 145 | 15350946 | **Pancreatic** cancer: future outlook, promising trials, newer systemic agents, and strategies from the Gastrointestinal Intergroup **Pancreatic** Cancer Task Force. |
| 146 | 9361591 | Low mortality following resection for **pancreatic** and periampullary tumours in 1026 patients: UK survey of specialist **pancreatic** units. UK **Pancreatic** Cancer Group. |
| 147 | 7531334 | The significance of CD44 in human **pancreatic** cancer: I. High expression of CD44 in human **pancreatic** adenocarcinoma. |
| 148 | 8412180 | **Pancreatic** ductal cell carcinoma producing **pancreatic** elastase 1. |
| 149 | 9088948 | Metastatic cancer involving **pancreatic** duct epithelium and its mimicry of primary **pancreatic** cancer. |
| 150 | 8672751 | **Pancreatic** calculi superimposed upon slow growing **pancreatic** cancer. |
| 151 | 7660249 | Familial **pancreatic** cancer and the genetics of **pancreatic** cancer. |
| 152 | 25056586 | **Pancreatic** metastasectomy: experience of the Irish National Surgical Centre for **Pancreatic** Cancer. |
| 153 | 23846938 | A proteomic comparison of formalin-fixed paraffin-embedded **pancreatic** tissue from autoimmune **pancreatitis**, chronic **pancreatitis**, and **pancreatic** cancer. |
| 154 | 19654471 | [A case of a small **pancreatic** cancer with an intact main **pancreatic** duct]. |
| 155 | 3528667 | **Pancreatic** insulin secretion in exocrine **pancreatic** cancer. |
| 156 | 21664851 | [New perspectives for radiosensitization in **pancreatic** carcinoma: a review of mechanisms involved in **pancreatic** tumorigenesis]. |
| 157 | 2376901 | [Clinical study of human **pancreatic** cancer-associated antigen (SPan-1 antigen) in hepatobiliary and **pancreatic** diseases]. |
| 158 | 12120267 | **Pancreatitis**-associated protein levels in **pancreatic** juice from patients with **pancreatic** diseases. |
| 159 | 24924775 | The early detection of **pancreatic** cancer: what will it take to diagnose and treat curable **pancreatic** neoplasia? |
| 160 | 18694521 | **Pancreatic** tuberculosis with splenic tuberculosis mimicking advanced **pancreatic** cancer with splenic metastasizes: a case report. |
| 161 | 9613128 | [Telomerase activity in **pancreatic** juice for the preoperative diagnosis of **pancreatic** cancer]. |
| 162 | 22793268 | Relationships of hepatic and **pancreatic** biomarkers with the cholestatic syndrome and tumor stage in **pancreatic** cancer. |
| 163 | 25252821 | TRAF6 is over-expressed in **pancreatic** cancer and promotes the tumorigenicity of **pancreatic** cancer cells. |
| 164 | 17152489 | [Significance of the mitochondrial D-loop alterations in hyperplastic **pancreatic** ductal cells in the vicinity of **pancreatic** cancer coexisting with chronic **pancreatitis**]. |
| 165 | 21558392 | **Pancreatic** stellate cells radioprotect **pancreatic** cancer cells through β1-integrin signaling. |
| 166 | 7272914 | **Pancreatic** secretions as a clue to the presence of **pancreatic** cancer. |
| 167 | 18650622 | Cisplatin, fluorouracil, interferon-alpha, and radiation as adjuvant therapy for resected **pancreatic** cancer: is there a future for this regimen andor should we change our approach to research and treatment of patients with **pancreatic** cancer? |
| 168 | 12696069 | Chromosomal instability in **pancreatic** ductal cells from patients with chronic **pancreatitis** and **pancreatic** adenocarcinoma. |
| 169 | 10659590 | Differential diagnosis between tumor-forming **pancreatitis** and **pancreatic** cancer by percutaneous transhepatic portography and selective direct **pancreatic** venography. |
| 170 | 7611198 | **Pancreatic** juice 90K and serum CA 19-9 combined determination can discriminate between **pancreatic** cancer and chronic **pancreatitis**. |
| 171 | 6084495 | **Pancreatic** amylase as a tumour marker for **pancreatic** cancer. |
| 172 | 16804974 | Overexpression of c-met in the early stage of **pancreatic** carcinogenesis; altered expression is not sufficient for progression from chronic **pancreatitis** to **pancreatic** cancer. |
| 173 | 21749982 | Genetic evolution of **pancreatic** cancer: lessons learnt from the **pancreatic** cancer genome sequencing project. |
| 174 | 3422536 | Estimation of carbohydrate antigen (CA) 19-9 levels in pure **pancreatic** juice of patients with **pancreatic** cancer. |
| 175 | 527874 | Trypsin and lactoferrin levels in pure **pancreatic** juice in patients with **pancreatic** disease. |
| 176 | 6102899 | **Pancreatic** juice gamma-glutamyltransferase, alanine transaminase, and alkaline phosphatase in **pancreatic** disease. |
| 177 | 2478412 | Enzyme immunoassay for serum **pancreatic** lipase in the diagnosis of **pancreatic** diseases. |
| 178 | 25169532 | Clinical efficacy of endoscopic **pancreatic** drainage for pain relief with malignant **pancreatic** duct obstruction. |
| 179 | 15953675 | Suppression of **pancreatic** tumor progression by systemic delivery of a **pancreatic**-cancer-specific promoter driven Bik mutant. |
| 180 | 22767586 | **Pancreatitis** and **pancreatic** cancer risk: a pooled analysis in the International **Pancreatic** Cancer Case-Control Consortium (PanC4). |
| 181 | 7589440 | Human **pancreatic** phospholipase A2 stimulates the growth of human **pancreatic** cancer cell line. |
| 182 | 7531335 | The significance of CD44 in human **pancreatic** cancer: II. The role of CD44 in human **pancreatic** adenocarcinoma invasion. |
| 183 | 19161187 | **Pancreatic** diffusion-weighted imaging (DWI): comparison between mass-forming focal **pancreatitis** (FP), **pancreatic** cancer (PC), and normal pancreas. |
| 184 | 17610286 | Focal **pancreatic** mass: distinction of **pancreatic** cancer from chronic **pancreatitis** using gadolinium-enhanced 3D-gradient-echo MRI. |
| 185 | 19896094 | Germ-line mutations, **pancreatic** inflammation, and **pancreatic** cancer. |
| 186 | 7974428 | [Analysis of Ki-ras in **pancreatic** fluid aspirate. A new diagnostic possibility in investigation of **pancreatic** cancer]. |
| 187 | 7203382 | Immunological diagnosis of **pancreatic** cancer by assaying carcinoembryonic antigen (CEA) in pure **pancreatic** juice. |
| 188 | 19620430 | Utility of 18F-FDG PETCT for differentiation of autoimmune **pancreatitis** with atypical **pancreatic** imaging findings from **pancreatic** cancer. |
| 189 | 21688462 | [Laparoscopic **pancreatic** resection of **pancreatic** cancer]. |
| 190 | 6201996 | Correlation between serum concentrations of three specific exocrine **pancreatic** proteins and **pancreatic** duct morphology at ERCP examinations. |
| 191 | 15753605 | Molecular analysis of **pancreatic** juice: a helpful tool to differentiate benign and malignant **pancreatic** tumors? |
| 192 | 19899942 | Second-generation endoscopic ultrasound elastography in the differential diagnosis of solid **pancreatic** masses. **Pancreatic** cancer vs. inflammatory mass in chronic **pancreatitis**. |
| 193 | 8501356 | Serum **pancreatic** stone protein in **pancreatic** diseases. |
| 194 | 3861883 | [Diagnostic usefulness of serum levels of **pancreatic** oncofetal antigen (POA) and CA 19-9 in **pancreatic** cancers: monitoring of postoperative recurrences]. |
| 195 | 25236592 | Duodenal ischemia and upper GI bleeding are dose-limiting toxicities of 24-h continuous intra-arterial **pancreatic** perfusion of gemcitabine following vascular isolation of the **pancreatic** head: early results from the Regional Chemotherapy in Locally Advanced **Pancreatic** Cancer (RECLAP) study. |
| 196 | 14991579 | Expression of cancer testis antigens in **pancreatic** carcinoma cell lines, **pancreatic** adenocarcinoma and chronic **pancreatitis**. |
| 197 | 25336120 | Increased N-glycosylation of Asn⁸⁸ in serum **pancreatic** ribonuclease 1 is a novel diagnostic marker for **pancreatic** cancer. |
| 198 | 2431845 | Serum immunoreactive elastase in diagnosis of **pancreatic** diseases. A sensitive marker for **pancreatic** cancer. |
| 199 | 19147554 | **Pancreatic** cancer and precursor **pancreatic** intraepithelial neoplasia lesions are devoid of primary cilia. |
| 200 | 3145303 | Tissue polypeptide antigen, galactosyltransferase isoenzyme II and **pancreatic** oncofetal antigen serum determination: role in **pancreatic** cancer diagnosis. |
| 201 | 25164613 | **Pancreatic** enzyme replacement therapy (PERT) for malabsorption in patients with metastatic **pancreatic** cancer. |
| 202 | 18794070 | **Pancreatic** cancer stem cells: implications for the treatment of **pancreatic** cancer. |
| 203 | 3465666 | Determination of CA 19-9 antigen in serum and **pancreatic** juice for differential diagnosis of **pancreatic** adenocarcinoma from chronic **pancreatitis**. |
| 204 | 11385248 | Experimental animal models in **pancreatic** carcinogenesis: lessons for human **pancreatic** cancer. |
| 205 | 10408907 | Higher frequency of DPC4Smad4 alterations in **pancreatic** cancer cell lines than in primary **pancreatic** adenocarcinomas. |
| 206 | 1356420 | Partial pancreaticoduodenectomy (Whipple procedure) for **pancreatic** malignancy: occlusion of a non-anastomosed **pancreatic** stump with fibrin sealant. |
| 207 | 24155642 | Eastern Canadian Colorectal Cancer Consensus Conference: standards of care for the treatment of patients with rectal, **pancreatic**, and gastrointestinal stromal tumours and **pancreatic** neuroendocrine tumours. |
| 208 | 21628592 | In vivo diagnosis of murine **pancreatic** intraepithelial neoplasia and early-stage **pancreatic** cancer by molecular imaging. |
| 209 | 24839966 | **Pancreatic** Cancer Database: an integrative resource for **pancreatic** cancer. |
| 210 | 6744282 | Simultaneous evaluation of a pancreas-specific antigen and a **pancreatic** cancer-associated antigen in **pancreatic** carcinoma. |
| 211 | 9444549 | Clinicopathologic study on **pancreatic** cancer associated with **pancreatic** stones. |
| 212 | 6713412 | Radioimmunoassay for human **pancreatic** ribonuclease and measurement of serum immunoreactive **pancreatic** ribonuclease in patients with malignant tumors. |
| 213 | 3785134 | [Studies on a **pancreatic** oncofetal antigen (POA) in patients with **pancreatic** cancer]. |
| 214 | 431121 | The relationships between **pancreatic** ductal obstruction and **pancreatic** secretion in man. |
| 215 | 21547133 | Study on chronic **pancreatitis** and **pancreatic** cancer using MRS and **pancreatic** juice samples. |
| 216 | 9438605 | International documentation system for **pancreatic** cancer (IDS). The future in **pancreatic** cancer evaluation. |
| 217 | 11677475 | **Pancreatic** intraductal sampling during ERCP in patients with chronic **pancreatitis** and **pancreatic** cancer: cytologic studies and k-ras-2 codon 12 molecular analysis in 47 cases. |
| 218 | 22119988 | IRF-2 is over-expressed in **pancreatic** cancer and promotes the growth of **pancreatic** cancer cells. |
| 219 | 4089524 | **Pancreatic** cancer in the Faroe Islands. An epidemiologic study of patients with **pancreatic** cancer in the Faroe Islands 1972-82. |
| 220 | 9177511 | **Pancreatic** duct stricture length at ERCP predicts tumor size and pathological stage of **pancreatic** cancer. |
| 221 | 2230667 | Diagnostic utility of a new monoclonal antibody **pancreatic** isoamylase assay in chronic **pancreatic** diseases. |
| 222 | 12750293 | Molecular profiling of **pancreatic** adenocarcinoma and chronic **pancreatitis** identifies multiple genes differentially regulated in **pancreatic** cancer. |
| 223 | 12170018 | Evidence-based **pancreatic** head resection for **pancreatic** cancer and chronic **pancreatitis**. |
| 224 | 21721456 | **Pancreatic** panniculitis: a cutaneous presentation as an initial clue to the diagnosis of **pancreatic** cancer. |
| 225 | 21573647 | New radioimmunoassay for **pancreatic** cancer-associated antigen span-1 with reference to differential-diagnosis and monitoring in **pancreatic**-cancer. |
| 226 | 21481788 | Stat3Socs3 activation by IL-6 transsignaling promotes progression of **pancreatic** intraepithelial neoplasia and development of **pancreatic** cancer. |
| 227 | 21646760 | [A study of the usefulness of **pancreatic** juice cytology obtained via an endoscopic nasal **pancreatic** drainage (ENPD) tube]. |
| 228 | 17923760 | Clinicopathological aspects of 542 cases of **pancreatic** cancer: a special emphasis on small **pancreatic** cancer. |
| 229 | 25078025 | The role of **pancreatic** and duodenal homeobox 1 as a therapeutic target in **pancreatic** cancer. |
| 230 | 8803695 | Laparoscopic treatment of **pancreatic** disorders: diagnosis and staging, palliation of cancer and treatment of **pancreatic** pseudocysts. |
| 231 | 15502640 | The molecular basis of **pancreatic** fibrosis: common stromal gene expression in chronic **pancreatitis** and **pancreatic** adenocarcinoma. |
| 232 | 17294442 | Expression of neutral endopeptidase (NEPCD10) on **pancreatic** tumor cell lines, **pancreatitis** and **pancreatic** tumor tissues. |
| 233 | 8195643 | Overexpression of **pancreatic** secretory trypsin inhibitor in **pancreatic** cancer. Evaluation of its biological function as a growth factor. |
| 234 | 102467 | A study of **pancreatic** secretory and intracellular enzymes in **pancreatic** cancer tissue, other gastrointestinal cancers, normal pancreas and serum. |
| 235 | 25840689 | Role of **pancreatic** stellate cells and periostin in **pancreatic** cancer progression. |
| 236 | 6349542 | [Evaluation of **pancreatic** oncofetal antigen (POA) in the diagnosis of **pancreatic** cancer]. |
| 237 | 14722685 | p-[123I]iodo-L-phenylalanine for detection of **pancreatic** cancer: basic investigations of the uptake characteristics in primary human **pancreatic** tumour cells and evaluation in in vivo models of human **pancreatic** adenocarcinoma. |
| 238 | 24987871 | Absence of **pancreatic** intraepithelial neoplasia predicts poor survival after resection of **pancreatic** cancer. |
| 239 | 15350935 | Conclusions from the European Study Group for **Pancreatic** Cancer adjuvant trial of chemoradiotherapy and chemotherapy for **pancreatic** cancer. |
| 240 | 18283486 | Morphogenesis of **pancreatic** cancer: role of **pancreatic** intraepithelial neoplasia (PanINs). |
| 241 | 877854 | **Pancreatic** secretion in hamsters with **pancreatic** cancer. |
| 242 | 18607507 | Human **pancreatic** cancer stem cells: implications for how we treat **pancreatic** cancer. |
| 243 | 2086001 | [Diagnosis of **pancreatic** cancer with simultaneous detection of a **pancreatic**-cancer-associated antigen and pancreas-specific antigen]. |
| 244 | 9175881 | Activation of MAP kinase cascade induced by human **pancreatic** phospholipase A2 in a human **pancreatic** cancer cell line. |
| 245 | 25863127 | miR-29c suppresses **pancreatic** cancer liver metastasis in an orthotopic implantation model in nude mice and affects survival in **pancreatic** cancer patients. |
| 246 | 7338341 | [Evaluation of **pancreatic** scintigram in the diagnosis of **pancreatic** diseases (author's transl)]. |
| 247 | 10049697 | Smad6 suppresses TGF-beta-induced growth inhibition in COLO-357 **pancreatic** cancer cells and is overexpressed in **pancreatic** cancer. |
| 248 | 11961486 | Matrix metalloproteinase-2 in **pancreatic** juice for diagnosis of **pancreatic** cancer. |
| 249 | 21450558 | **Pancreatic** enzyme replacement therapy for **pancreatic** exocrine insufficiency: when is it indicated, what is the goal and how to do it? |
| 250 | 6704974 | Immunological assay of **pancreatic** ribonuclease in serum as an indicator of **pancreatic** cancer. |
| 251 | 24622469 | Association of **pancreatic** Fatty infiltration with **pancreatic** ductal adenocarcinoma. |
| 252 | 25176058 | BMP8B mediates the survival of **pancreatic** cancer cells and regulates the progression of **pancreatic** cancer. |
| 253 | 21932188 | A case of small **pancreatic** cancer with intra-**pancreatic** metastasis diagnosed by endoscopic ultrasound. |
| 254 | 21242706 | **Pancreatic** cancer: the role of **pancreatic** stellate cells in tumor progression. |
| 255 | 25731473 | [A case of **pancreatic** pseudocyst associated with **pancreatic** cancer successfully treated with endoscopic pseudocyst drainage, which allowed continuation of chemotherapy]. |
| 256 | 17907457 | [**Pancreatic** cancer--characteristics of the Japanese Guidelines for the Diagnosis and Treatment of **Pancreatic** Cancer and my opinion on this subject]. |
| 257 | 21963224 | An RNA aptamer that specifically binds **pancreatic** adenocarcinoma up-regulated factor inhibits migration and growth of **pancreatic** cancer cells. |
| 258 | 20624967 | Notch2 is required for progression of **pancreatic** intraepithelial neoplasia and development of **pancreatic** ductal adenocarcinoma. |
| 259 | 22510406 | **Pancreatic** stellate cells enhance stem cell-like phenotypes in **pancreatic** cancer cells. |
| 260 | 2503584 | Cytokines and **pancreatic** cancer. Sensitivity of xenotransplants of predominantly **pancreatic** carcinomas to rIFN-gamma and rTFN-alpha in nude mice. |
| 261 | 23206934 | Genetic susceptibility to **pancreatic** cancer and its functional characterisation: the **PANcreatic** Disease ReseArch (PANDoRA) consortium. |
| 262 | 25258651 | Circulating microRNAs in **Pancreatic** Juice as Candidate Biomarkers of **Pancreatic** Cancer. |
| 263 | 19755770 | Small **pancreatic** cancer with pancreas divisum preoperatively diagnosed by **pancreatic** juice cytology. |
| 264 | 8444083 | Simultaneous determinations of **pancreatic** phospholipase A2 and prophospholipase A2 in various **pancreatic** diseases. |
| 265 | 19403515 | Proteomic characterisation of **pancreatic** islet beta-cells stimulated with **pancreatic** carcinoma cell conditioned medium. |
| 266 | 25356582 | **Pancreatic** cell tracing, lineage tagging and targeted genetic manipulations in multiple cell types using **pancreatic** ductal infusion of adeno-associated viral vectors andor cell-tagging dyes. |
| 267 | 1706672 | The diagnostic value of serum **pancreatic** phospholipase A2 (PLA2) in **pancreatic** diseases. |
| 268 | 19260743 | **Pancreatic** intraepithelial neoplasia and **pancreatic** tumorigenesis: of mice and men. |
| 269 | 2509154 | The diagnostic significance of carbohydrate antigen CA 19-9 in serum and **pancreatic** juice in **pancreatic** carcinoma. |
| 270 | 8661216 | Insulin promotes **pancreatic** cancer: evidence for endocrine influence on exocrine **pancreatic** tumors. |
| 271 | 9772067 | Telomerase activity detected in **pancreatic** juice 19 months before a tumor is detected in a patient with **pancreatic** cancer. |
| 272 | 25731618 | Wnt2 protein plays a role in the progression of **pancreatic** cancer promoted by **pancreatic** stellate cells. |
| 273 | 8230832 | [Usefulness of serum **pancreatic** phospholipase A2 determination in patients with various **pancreatic** diseases]. |
| 274 | 6197338 | Serum **pancreatic** oncofetal antigen: its clinical usefulness for screening **pancreatic** cancer in combination with tests for other tumor markers. |
| 275 | 20875068 | **Pancreatic** intraepithelial neoplasia-can we detect early **pancreatic** cancer? |
| 276 | 25537257 | Automated **pancreatic** cyst screening using natural language processing: a new tool in the early detection of **pancreatic** cancer. |
| 277 | 1385247 | [Transfection of **pancreatic** acinar cells (AR4-2J) by bFGF modifies cell morphology and biosynthesis of **pancreatic** secretory enzymes]. |
| 278 | 15451430 | **Pancreatic** cancer stimulates **pancreatic** stellate cell proliferation and TIMP-1 production through the MAP kinase pathway. |
| 279 | 23011021 | [New insights into the origin of **pancreatic** cancer. Role of atypical flat lesions in **pancreatic** carcinogenesis]. |
| 280 | 7025732 | Partial characterisation of an oncofetal **pancreatic** antigen. Its role in the differential diagnosis and therapy of patients with **pancreatic** cancer. |
| 281 | 11280537 | Early detection of **pancreatic** cancer in patients with chronic **pancreatitis**: diagnostic utility of a K-ras point mutation in the **pancreatic** juice. |
| 282 | 12373299 | **Pancreatic** elastase IIIA and its variants are expressed in **pancreatic** carcinoma cells. |
| 283 | 24687867 | **Pancreatic** enzyme replacement therapy during **pancreatic** insufficiency. |
| 284 | 21499212 | **Pancreatic** ductal adenocarcinoma derived from IPMN and **pancreatic** ductal adenocarcinoma concomitant with IPMN. |
| 285 | 23791528 | An iPSC line from human **pancreatic** ductal adenocarcinoma undergoes early to invasive stages of **pancreatic** cancer progression. |
| 286 | 19110611 | Role of epidermal growth factor gene in the development of **pancreatic** cancer and efficiency of inhibitors of this gene in the treatment of **pancreatic** carcinoma. |
| 287 | 8495407 | Detection of ras gene mutations in **pancreatic** juice and peripheral blood of patients with **pancreatic** adenocarcinoma. |
| 288 | 3458359 | CA 19-9 assay in differential diagnosis of **pancreatic** carcinoma from inflammatory **pancreatic** diseases. |
| 289 | 25662149 | [**Pancreatic** cancer. Evidence based management guidelines of the Hungarian **Pancreatic** Study Group]. |
| 290 | 15000259 | Changes of lymphatic flow in case of **pancreatic** duct obstruction in the pig--as a model of **pancreatic** cancer. |
| 291 | 24780634 | Contextual regulation of **pancreatic** cancer stem cell phenotype and radioresistance by **pancreatic** stellate cells. |
| 292 | 18516541 | Complete remission of **pancreatic** cancer after multiple resections of locally **pancreatic** recurrent sites and liver metastasis: report of a case. |
| 293 | 18075308 | Sensitive and quantitative detection of KRAS2 gene mutations in **pancreatic** duct juice differentiates patients with **pancreatic** cancer from chronic **pancreatitis**, potential for early detection. |
| 294 | 6191979 | Radioimmunoassay of human **pancreatic** elastase 1. In vitro interaction of human **pancreatic** elastase 1 with serum protease inhibitors. |
| 295 | 14599992 | Detection of K-ras gene mutation at codon 12 by **pancreatic** duct brushing for **pancreatic** cancer. |
| 296 | 23404405 | Lactate dehydrogenase A is overexpressed in **pancreatic** cancer and promotes the growth of **pancreatic** cancer cells. |
| 297 | 24503018 | Nuclear translocation of FGFR1 and FGF2 in **pancreatic** stellate cells facilitates **pancreatic** cancer cell invasion. |
| 298 | 6205469 | Establishment of a human **pancreatic** cancer cell line and detection of **pancreatic** cancer associated antigen. |
| 299 | 25727013 | **Pancreatic** adenocarcinoma upregulated factor (PAUF) confers resistance to **pancreatic** cancer cells against oncolytic parvovirus H-1 infection through IFNA receptor-mediated signaling. |
| 300 | 23975591 | **Pancreatic** cancer in the remnant pancreas following primary **pancreatic** resection. |
| 301 | 8269629 | Enhanced expression of annexin II in human **pancreatic** carcinoma cells and primary **pancreatic** cancers. |
| 302 | 14520282 | Severe localized stenosis and marked dilatation of the main **pancreatic** duct are indicators of **pancreatic** cancer instead of chronic **pancreatitis** on endoscopic retrograde balloon pancreatography. |
| 303 | 15604267 | Protein expression profiles in **pancreatic** adenocarcinoma compared with normal **pancreatic** tissue and tissue affected by **pancreatitis** as detected by two-dimensional gel electrophoresis and mass spectrometry. |
| 304 | 19896099 | Roles of **pancreatic** stellate cells in **pancreatic** inflammation and fibrosis. |
| 305 | 23682805 | Translating discovery in zebrafish **pancreatic** development to human **pancreatic** cancer: biomarkers, targets, pathogenesis, and therapeutics. |
| 306 | 11854560 | Chronic **pancreatitis**: relationship to acute **pancreatitis** and **pancreatic** cancer. |
| 307 | 12739550 | Placement of self-expanding metallic stents in the **pancreatic** duct for treatment of obstructive complications of **pancreatic** cancer. |
| 308 | 22699201 | Incidental **pancreatic** cysts found by magnetic resonance imaging and their relationship with **pancreatic** cancer. |
| 309 | 22320920 | Dangerous liaisons: **pancreatic** stellate cells and **pancreatic** cancer cells. |
| 310 | 23403949 | ABO blood groups and **pancreatic** cancer risk and survival: results from the **PANcreatic** Disease ReseArch (PANDoRA) consortium. |
| 311 | 17548257 | Diagnosis of **pancreatic** cancer by cytology and telomerase activity in exfoliated cells obtained by **pancreatic** duct brushing during endoscopy. |
| 312 | 7962312 | Clinical review 63: Diabetes and **pancreatic** cancer: clues to the early diagnosis of **pancreatic** malignancy. |
| 313 | 15362786 | **Pancreatic** liver metastases after curative resection combined with intraoperative radiation for **pancreatic** cancer. |
| 314 | 22749856 | Targeted destruction of the orchestration of the **pancreatic** stroma and tumor cells in **pancreatic** cancer cases: molecular basis for therapeutic implications. |
| 315 | 11561004 | Expression of nerve growth factors in **pancreatic** neural tissue and **pancreatic** cancer. |
| 316 | 18395901 | **Pancreatic** stellate cells promote proliferation and invasiveness of human **pancreatic** cancer cells via galectin-3. |
| 317 | 23565475 | **Pancreatic** carcinoma in fibrocalcific **pancreatic** diabetes: An eastern India perspective. |
| 318 | 19756884 | CC chemokine receptor 9 enhances proliferation in **pancreatic** intraepithelial neoplasia and **pancreatic** cancer cells. |
| 319 | 19722229 | Survival analysis after **pancreatic** resection for ampullary and **pancreatic** head carcinoma: an analysis of clinicopathological factors. |
| 320 | 12636100 | Correlation between peri-operative serum lactate levels and outcome in **pancreatic** resection for **pancreatic** cancer, preliminary report. |
| 321 | 17357096 | Histological complexities of **pancreatic** lesions from transgenic mouse models are consistent with biological and morphological heterogeneity of human **pancreatic** cancer. |
| 322 | 9735394 | Alteration of the CDKN2A gene in **pancreatic** cancers: Is it a late event in the progression of **pancreatic** cancer? |
| 323 | 23236243 | Small serotonin-positive **pancreatic** endocrine tumors caused obstruction of the main **pancreatic** duct. |
| 324 | 22944373 | The effect of octreotide treatment on patients with **pancreatic** cancer who undergo endoscopic retrograde cholangiopancreatography (ERCP) with **pancreatic** duct stent placement. |
| 325 | 2315288 | Polyamine concentrations in **pancreatic** tissue, serum, and urine of patients with **pancreatic** cancer. |
| 326 | 6197334 | Comparative study of serum **pancreatic** isoamylase, lipase, and trypsin-like immunoreactivity in **pancreatic** disease. |
| 327 | 21114434 | A prediction rule for estimating **pancreatic** cancer risk in chronic **pancreatitis** patients with focal **pancreatic** mass lesions with prior negative EUS-FNA cytology. |
| 328 | 8208970 | F-18 fluorodeoxyglucose PET in vivo evaluation of **pancreatic** glucose metabolism for detection of **pancreatic** cancer. |
| 329 | 20373013 | Alcohol intake and **pancreatic** cancer: a pooled analysis from the **pancreatic** cancer cohort consortium (PanScan). |
| 330 | 20934972 | Role of **pancreatic** stellate cells in **pancreatic** cancer metastasis. |
| 331 | 10982633 | Resectable carcinoma of the **pancreatic** head developing 7 years and 4 months after distal pancreatectomy for carcinoma of the **pancreatic** tail. |
| 332 | 21359597 | Patterns of **pancreatic** resection differ between patients with familial and sporadic **pancreatic** cancer. |
| 333 | 22104574 | Cigarette smoking and **pancreatic** cancer: an analysis from the International **Pancreatic** Cancer Case-Control Consortium (Panc4). |
| 334 | 25865695 | Mutations in the p16 gene in DMBA-induced **pancreatic** intraepithelial neoplasia and **pancreatic** cancer in rats. |
| 335 | 10484017 | Diagnosis of **pancreatic** cancer by detecting telomerase activity in **pancreatic** juice: comparison with K-ras mutations. |
| 336 | 20442678 | Inhibition of **pancreatic** stellate cell activation by halofuginone prevents **pancreatic** xenograft tumor development. |
| 337 | 19689742 | Identification of a novel murine **pancreatic** tumour antigen, which elicits antibody responses in patients with **pancreatic** carcinoma. |
| 338 | 23112111 | Diabetes and risk of **pancreatic** cancer: a pooled analysis from the **pancreatic** cancer cohort consortium. |
| 339 | 19034981 | Value of ultrasound examination in differential diagnosis of **pancreatic** lymphoma and **pancreatic** cancer. |
| 340 | 19592030 | Tumor-derived **pancreatic** stellate cells promote **pancreatic** cancer cell invasion through release of thrombospondin-2. |
| 341 | 12235871 | Surgical treatment for chronic **pancreatitis**: results of **pancreatic** duct drainage operation and **pancreatic** resection. |
| 342 | 8460089 | Diagnostic significance of cancer-associated carbohydrate antigen (CA19-9) concentrations in **pancreatic** juice: analysis in pure **pancreatic** juice collected by endoscopic aspiration and immunohistochemical study in chronic **pancreatitis**. |
| 343 | 18336654 | **Pancreatic** stellate cells: molecular mechanism of **pancreatic** fibrosis. |
| 344 | 11501843 | Diagnostic application of CD44 variant expression in **pancreatic** juice for detection of **pancreatic** neoplasm. |
| 345 | 19342980 | Usefulness of human telomerase reverse transcriptase in **pancreatic** juice as a biomarker of **pancreatic** malignancy. |
| 346 | 1875897 | [The growth rates of liver metastases in **pancreatic** cancer--comparison on growth rates between clinical cases and established human **pancreatic** cancer cell lines]. |
| 347 | 21541631 | Growth of human **pancreatic** cancer cells, induced by human **pancreatic** phospholipase A(2), is mediated via its specific receptor but not via its catalytic property. |
| 348 | 2498436 | Cytokines and **pancreatic** cancer. The effect of rIFN-gamma, HuLeIFN, rTNF-alpha, and LAK-cells on **pancreatic** and other gastrointestinal tumors in vitro. |
| 349 | 12518126 | Cost-effectiveness of **pancreatic** cancer screening in familial **pancreatic** cancer kindreds. |
| 350 | 8407563 | Identification of K-ras oncogene mutations in the pure **pancreatic** juice of patients with ductal **pancreatic** cancers. |
| 351 | 22677939 | Overexpression of CIAPIN1 inhibited **pancreatic** cancer cell proliferation and was associated with good prognosis in **pancreatic** cancer. |
| 352 | 16083499 | **Pancreatic** stellate cells (PSCs) express cyclooxygenase-2 (COX-2) and **pancreatic** cancer stimulates COX-2 in PSCs. |
| 353 | 19561064 | Cigarette smoking and **pancreatic** cancer: a pooled analysis from the **pancreatic** cancer cohort consortium. |
| 354 | 12406190 | Minute **pancreatic** adenocarcinoma presenting with stenosis of the main **pancreatic** duct. |
| 355 | 2410346 | Multiparametric tumor marker (CA 19-9, CEA, AFP, POA) analyses of **pancreatic** juices and sera in **pancreatic** diseases. |
| 356 | 10891365 | Epiregulin is Up-regulated in **pancreatic** cancer and stimulates **pancreatic** cancer cell growth. |
| 357 | 21536662 | Alcohol consumption and **pancreatic** cancer: a pooled analysis in the International **Pancreatic** Cancer Case-Control Consortium (PanC4). |
| 358 | 316012 | **Pancreatic** elastase and serum alpha 1-antitrypsin levels in beagle dogs smoking high- and low-nicotine cigarettes: possible mechanism of **pancreatic** cancer in cigarette smokers. |
| 359 | 23555989 | Perineural mast cells are specifically enriched in **pancreatic** neuritis and neuropathic pain in **pancreatic** cancer and chronic **pancreatitis**. |
| 360 | 19476877 | **Pancreatic** steatosis promotes dissemination and lethality of **pancreatic** cancer. |
| 361 | 19690177 | Polymorphic variants in hereditary **pancreatic** cancer genes are not associated with **pancreatic** cancer risk. |
| 362 | 15983444 | Gene expression analysis of **pancreatic** cell lines reveals genes overexpressed in **pancreatic** cancer. |
| 363 | 1632977 | Crude isolation of DNA from unselected human **pancreatic** tissue and amplification by the polymerase chain reaction of Ki-ras oncogene to detect point mutations in **pancreatic** cancer. |
| 364 | 8515582 | [Effect of cholecystokinin and secretin on insulin binding to rat **pancreatic** acini and **pancreatic** cancer cell line AR42J cells]. |
| 365 | 3499996 | Growth of **pancreatic** foci and development of **pancreatic** cancer with a single dose of azaserine in the rat. |
| 366 | 12609076 | Detecting K-ras and p53 gene mutation from stool and **pancreatic** juice for diagnosis of early **pancreatic** cancer. |
| 367 | 18669622 | Hypoxia stimulates **pancreatic** stellate cells to induce fibrosis and angiogenesis in **pancreatic** cancer. |
| 368 | 22644446 | Selective reoperation for locally recurrent or metastatic **pancreatic** ductal adenocarcinoma following primary **pancreatic** resection. |
| 369 | 10732293 | An immunohistochemical study of the expression of bcl-2 and p53 oncoproteins in **pancreatic** intraepithelial neoplasia and **pancreatic** cancer. |
| 370 | 20484957 | **Pancreatic** stellate cells increase the invasion of human **pancreatic** cancer cells through the stromal cell-derived factor-1CXCR4 axis. |
| 371 | 20350215 | Differential cell cycle and proliferation marker expression in ductal **pancreatic** adenocarcinoma and **pancreatic** intraepithelial neoplasia (PanIN). |
| 372 | 18182742 | A case of **pancreatic** cancer with formation of a mass mimicking alcoholic or autoimmune **pancreatitis** in a young man. Possibility of diagnosis by hypermethylation of pure **pancreatic** juice. |
| 373 | 2436967 | Analysis of pure **pancreatic** juice proteins by two-dimensional gel electrophoresis in cases of **pancreatic** cancer. |
| 374 | 17876545 | Fecal calprotectin and elastase 1 determinations in patients with **pancreatic** diseases: a possible link between **pancreatic** insufficiency and intestinal inflammation. |
| 375 | 10517912 | High proportion of mutant K-ras gene in **pancreatic** juice of patients with **pancreatic** cystic lesions. |
| 376 | 9242339 | **Pancreatic** resection combined with intraoperative radiation therapy for **pancreatic** cancer. |
| 377 | 23820785 | Allergies and risk of **pancreatic** cancer: a pooled analysis from the **Pancreatic** Cancer Case-Control Consortium. |
| 378 | 9824353 | Stromelysin 3 is overexpressed in human **pancreatic** carcinoma and regulated by retinoic acid in **pancreatic** carcinoma cell lines. |
| 379 | 16284732 | Distribution of somatostatin in **pancreatic** ductal adenocarcinoma remodels the normal pattern of the protein during foetal **pancreatic** development: an immunohistochemical analysis. |
| 380 | 20550709 | Elevated level of anterior gradient-2 in **pancreatic** juice from patients with pre-malignant **pancreatic** neoplasia. |
| 381 | 18272475 | Randomized phase III trial of adjuvant chemotherapy with gemcitabine versus S-1 in patients with resected **pancreatic** cancer: Japan Adjuvant Study Group of **Pancreatic** Cancer (JASPAC-01). |
| 382 | 892342 | Prospective evaluation of the **pancreatic** secretion of immunoreactive carcinoembryonic antigen, enzyme, and bicarbonate in patients suspected of having **pancreatic** cancer. |
| 383 | 23438477 | Risk factors for **pancreatic** ductal adenocarcinoma specifically stimulate **pancreatic** duct glands in mice. |
| 384 | 7745821 | Evaluation of the effect of **pancreatic** resection in advanced **pancreatic** cancer with special reference using hospital-free survival as a measure of quality of life. |
| 385 | 23561966 | The role of **pancreatic** stellate cells in **pancreatic** cancer. |
| 386 | 11474289 | Familial fibrocystic **pancreatic** atrophy with endocrine cell hyperplasia and **pancreatic** carcinoma. |
| 387 | 23970016 | Ulcer, gastric surgery and **pancreatic** cancer risk: an analysis from the International **Pancreatic** Cancer Case-Control Consortium (PanC4). |
| 388 | 11249034 | Treatment of **pancreatic** cancer with a combination of docetaxel, gemcitabine and granulocyte colony-stimulating factor: a phase II study of the Greek Cooperative Group for **Pancreatic** Cancer. |
| 389 | 8139120 | [Progress of preoperative diagnosis of **pancreatic** tumor--with special reference to extension of **pancreatic** cancer]. |
| 390 | 10840305 | Telomerase activity in pure **pancreatic** juice for the diagnosis of **pancreatic** cancer may be complementary to K-ras mutation. |
| 391 | 21717311 | Accuracy of differential diagnosis for **pancreatic** cancer is improved in the combination of RCAS1 and CEA measurements and cytology in **pancreatic** juice. |
| 392 | 22166947 | Routine testing for PALB2 mutations in familial **pancreatic** cancer families and breast cancer families with **pancreatic** cancer is not indicated. |
| 393 | 23360791 | NPTX2 hypermethylation in pure **pancreatic** juice predicts **pancreatic** neoplasms. |
| 394 | 22513235 | **Pancreatic** stellate cells promotes the perineural invasion in **pancreatic** cancer. |
| 395 | 10231847 | Dissociated insulin and islet amyloid polypeptide secretion from isolated rat **pancreatic** islets cocultured with human **pancreatic** adenocarcinoma cells. |
| 396 | 17469085 | The expression of S100A8 in **pancreatic** cancer-associated monocytes is associated with the Smad4 status of **pancreatic** cancer cells. |
| 397 | 17703087 | **Pancreatic** stellate cells potentiate proinvasive effects of SERPINE2 expression in **pancreatic** cancer xenograft tumors. |
| 398 | 7846018 | Evaluation of cytology and tumor markers of pure **pancreatic** juice for the diagnosis of **pancreatic** cancer at early stages. |
| 399 | 15059921 | Prospective risk of **pancreatic** cancer in familial **pancreatic** cancer kindreds. |
| 400 | 22484812 | Comparing human **pancreatic** cell secretomes by in vitro aptamer selection identifies cyclophilin B as a candidate **pancreatic** cancer biomarker. |
| 401 | 18381413 | **Pancreatic** stellate cells: partners in crime with **pancreatic** cancer cells. |
| 402 | 7774757 | Effects of **pancreatic** digestive enzymes, sodium bicarbonate, and a proton pump inhibitor on steatorrhoea caused by **pancreatic** diseases. |
| 403 | 12120000 | Frequent deletions of tumor suppressor genes in pure **pancreatic** juice from patients with tumoral or nontumoral **pancreatic** diseases. |
| 404 | 24308064 | Migratory activity of CD105+ **pancreatic** cancer cells is strongly enhanced by **pancreatic** stellate cells. |
| 405 | 23494611 | Clinical features and treatment outcome of borderline resectable **pancreatic** headbody cancer: a multi-institutional survey by the Japanese Society of **Pancreatic** Surgery. |
| 406 | 6160076 | Aging changes of **pancreatic** isoamylases and the appearance of "old amylase" in the serum of patients with **pancreatic** pseudocysts. |
| 407 | 24338808 | Prospective assessment of the influence of **pancreatic** cancer resection on exocrine **pancreatic** function. |
| 408 | 21245160 | Cigar and pipe smoking, smokeless tobacco use and **pancreatic** cancer: an analysis from the International **Pancreatic** Cancer Case-Control Consortium (PanC4). |
| 409 | 21472214 | Analysis of invasion-metastasis in **pancreatic** cancer: Correlation between the expression and arrangement of tight junction protein-2 and cell dissociation in **pancreatic** cancer cells. |
| 410 | 9707068 | A case of small **pancreatic** cancer diagnosed by serial follow-up studies promptly by a positive K-ras point mutation in pure **pancreatic** juice. |
| 411 | 7910839 | Measurement of sialylated stage-specific embryonic antigen-1 in pure **pancreatic** juice for the diagnosis of **pancreatic** cancer. |
| 412 | 21327822 | Recurrence pattern and prognosis of **pancreatic** cancer after **pancreatic** fistula. |
| 413 | 2463138 | Role of serum **pancreatic** enzyme assays in diagnosis of **pancreatic** disease. |
| 414 | 17012836 | Differentiating **pancreatic** lesions by microarray and QPCR analysis of **pancreatic** juice RNAs. |
| 415 | 1820014 | [Effect of synthetic protease inhibitor on the oncogenesis of **pancreatic** cancer in hamsters: study on **pancreatic** endocrine cells and free radicals]. |
| 416 | 21227721 | **Pancreatic** resection in the octogenarian: a safe option for **pancreatic** malignancy. |
| 417 | 19066953 | The monoclonal anti-BCL10 antibody (clone 331.1) is a sensitive and specific marker of **pancreatic** acinar cell carcinoma and **pancreatic** metaplasia. |
| 418 | 19724273 | S100A6 binds to annexin 2 in **pancreatic** cancer cells and promotes **pancreatic** cancer cell motility. |
| 419 | 21081113 | **Pancreatic** stellate cells promote epithelial-mesenchymal transition in **pancreatic** cancer cells. |
| 420 | 16003754 | FXYD3 is overexpressed in **pancreatic** ductal adenocarcinoma and influences **pancreatic** cancer cell growth. |
| 421 | 10521973 | Sialylated MUC1 mucin expression in normal pancreas, benign **pancreatic** lesions, and **pancreatic** ductal adenocarcinoma. |
| 422 | 15097858 | **Pancreatic** enzyme extract improves survival in murine **pancreatic** cancer. |
| 423 | 16952558 | Endogenous opioids inhibit early-stage **pancreatic** pain in a mouse model of **pancreatic** cancer. |
| 424 | 20579395 | MicroRNA, hsa-miR-200c, is an independent prognostic factor in **pancreatic** cancer and its upregulation inhibits **pancreatic** cancer invasion but increases cell proliferation. |
| 425 | 1886892 | Various tumor markers for small **pancreatic** cancer with special reference to the present status of **pancreatic** cancer in Japan and our experience over the past 2 years. |
| 426 | 23913634 | Influence of preoperative anti-cancer therapy on resectability and perioperative outcomes in patients with **pancreatic** cancer: project study by the Japanese Society of Hepato-Biliary-**Pancreatic** Surgery. |
| 427 | 15727932 | Establishing a murine **pancreatic** cancer CaSm model: up-regulation of CaSm is required for the transformed phenotype of murine **pancreatic** adenocarcinoma. |
| 428 | 8759672 | Diagnosis of **pancreatic** cancer by K-ras point mutation and cytology of **pancreatic** juice. |
| 429 | 17302733 | Loss of expression of antigen-presenting molecules in human **pancreatic** cancer and **pancreatic** cancer cell lines. |
| 430 | 17667542 | **Pancreatic** intraepithelial neoplasia in heterotopic pancreas: evidence for the progression model of **pancreatic** ductal adenocarcinoma. |
| 431 | 20103627 | **Pancreatic** cancer risk and ABO blood group alleles: results from the **pancreatic** cancer cohort consortium. |
| 432 | 3174607 | Vasoactive intestinal peptide inhibits the growth of hamster **pancreatic** cancer but not human **pancreatic** cancer in vivo. |
| 433 | 13678692 | **Pancreatic** duct obstruction itself induces expression of alpha smooth muscle actin in **pancreatic** stellate cells. |
| 434 | 24647860 | A triple combination of atorvastatin, celecoxib and tipifarnib strongly inhibits **pancreatic** cancer cells and xenograft **pancreatic** tumors. |
| 435 | 12730869 | Risk factors for the development of **pancreatic** cancer in familial **pancreatic** cancer kindreds. |
| 436 | 12163367 | 5-Lipoxygenase and leukotriene B(4) receptor are expressed in human **pancreatic** cancers but not in **pancreatic** ducts in normal tissue. |
| 437 | 25083089 | Pain sensation in **pancreatic** diseases is not uniform: the different facets of **pancreatic** pain. |
| 438 | 24023348 | Negative impact of fresh-frozen plasma transfusion on prognosis of **pancreatic** ductal adenocarcinoma after **pancreatic** resection. |
| 439 | 25750338 | Significance of histopathological evaluation of **pancreatic** fibrosis to predict postoperative course after **pancreatic** surgery. |
| 440 | 10408850 | Phase II study of docetaxel in patients with metastatic **pancreatic** cancer: a Japanese cooperative study. Cooperative Group of Docetaxel for **Pancreatic** Cancer in Japan. |
| 441 | 10436798 | Role of tumor markers and mutations in cells and **pancreatic** juice in the diagnosis of **pancreatic** cancer. |
| 442 | 17571071 | Both HIV- and EIAV-based lentiviral vectors mediate gene delivery to **pancreatic** cancer cells and human **pancreatic** primary patient xenografts. |
| 443 | 25003666 | Novel role of **pancreatic** differentiation 2 in facilitating self-renewal and drug resistance of **pancreatic** cancer stem cells. |
| 444 | 11075987 | Racial differences in **pancreatic** cancer: comparison of survival and histologic types of **pancreatic** carcinoma in Asians, blacks, and whites in the United States. |
| 445 | 15547748 | TU12B1-TY, a novel gene in the region at 12q22-q23.1 frequently deleted in **pancreatic** cancer, shows reduced expression in **pancreatic** cancer cells. |
| 446 | 23933230 | MicroRNAs as diagnostic markers for **pancreatic** ductal adenocarcinoma and its precursor, **pancreatic** intraepithelial neoplasm. |
| 447 | 23798558 | Laminin, gamma 2 (LAMC2): a promising new putative **pancreatic** cancer biomarker identified by proteomic analysis of **pancreatic** adenocarcinoma tissues. |
| 448 | 8651401 | The meaning of equivocal **pancreatic** cytology in patients thought to have **pancreatic** cancer. |
| 449 | 18525343 | Lessons from Tarceva in **pancreatic** cancer: where are we now, and how should future trials be designed in **pancreatic** cancer? |
| 450 | 22711213 | The daily practice of **pancreatic** enzyme replacement therapy after **pancreatic** surgery: a northern European survey: enzyme replacement after surgery. |
| 451 | 25132920 | Evaluation of diagnostic cytology via endoscopic naso-**pancreatic** drainage for **pancreatic** tumor. |
| 452 | 9745077 | Staging and treatment for patients with **pancreatic** cancer. How small is an early **pancreatic** cancer? |
| 453 | 11513873 | The anti-apoptotic protein BAG-3 is overexpressed in **pancreatic** cancer and induced by heat stress in **pancreatic** cancer cell lines. |
| 454 | 2140933 | [Missile therapy of colorectal and **pancreatic** cancers--clinical trial of monoclonal antibody, A7-NCS, in 73 patients with colorectal and **pancreatic** cancers]. |
| 455 | 20685603 | CD10+ **pancreatic** stellate cells enhance the progression of **pancreatic** cancer. |
| 456 | 11758254 | [An application value of detecting K-ras and p53 gene mutation in the stool and pure **pancreatic** juice for diagnosis of early **pancreatic** cancer]. |
| 457 | 12239630 | Cytokine regulation of constitutive production of interleukin-8 and -6 by human **pancreatic** cancer cell lines and serum cytokine concentrations in patients with **pancreatic** cancer. |
| 458 | 12451037 | Main **pancreatic** duct dilatation: a sign of high risk for **pancreatic** cancer. |
| 459 | 9261609 | Preoperative differential diagnosis of benign and malignant **pancreatic** lesions--the value of **pancreatic** secretory trypsin inhibitor, procarboxypeptidase B, CA19-9 and CEA. |
| 460 | 9888665 | Angiogenesis, angiogenic growth factors, and cell adhesion molecules are upregulated in chronic **pancreatic** diseases: angiogenesis in chronic **pancreatitis** and in **pancreatic** cancer. |
| 461 | 16424060 | DNA methylation alterations in the **pancreatic** juice of patients with suspected **pancreatic** disease. |
| 462 | 23912084 | Genetic alterations associated with progression from **pancreatic** intraepithelial neoplasia to invasive **pancreatic** tumor. |
| 463 | 22738386 | Factors influencing receptivity to future screening options for **pancreatic** cancer in those with and without **pancreatic** cancer family history. |
| 464 | 21630184 | Diagnostic value of quantitative EUS elastography for malignant **pancreatic** tumors: relationship with **pancreatic** fibrosis. |
| 465 | 22876381 | **Pancreatic** cancer and the tumor microenvironment: Mesenchyme’s role in **pancreatic** carcinogenesis |
| 466 | 22984426 | Unlike **pancreatic** cancer cells **pancreatic** cancer associated fibroblasts display minimal gene induction after 5-aza-2'-deoxycytidine. |
| 467 | 8927616 | Detection of K-ras point mutations at codon 12 in pure **pancreatic** juice for the diagnosis of **pancreatic** cancer by PCR-RFLP analysis. |
| 468 | 20473331 | The zinc-finger protein KCMF1 is overexpressed during **pancreatic** cancer development and downregulation of KCMF1 inhibits **pancreatic** cancer development in mice. |
| 469 | 10096564 | The FHIT gene is expressed in **pancreatic** ductular cells and is altered in **pancreatic** cancers. |
| 470 | 24650449 | Chronic stress accelerates **pancreatic** cancer growth and invasion: a critical role for beta-adrenergic signaling in the **pancreatic** microenvironment. |
| 471 | 18086771 | Distinctive heavy metal composition of **pancreatic** juice in patients with **pancreatic** carcinoma. |
| 472 | 10982594 | Significance of K-ras mutation and CEA level in **pancreatic** juice in the diagnosis of **pancreatic** cancer. |
| 473 | 24462078 | Use of a temporary intraoperative mesentericoportal shunt for **pancreatic** resection for locally advanced **pancreatic** cancer with portal vein occlusion and portal hypertension. |
| 474 | 25766398 | **Pancreatic** polypeptide response to a mixed meal is blunted in **pancreatic** head cancer associated with diabetes mellitus. |
| 475 | 10449979 | A surgical and pathological based classification of resective treatment of **pancreatic** cancer. Summary of an international workshop on surgical procedures in **pancreatic** cancer. |
| 476 | 23561977 | Improved survival and quality of life in patients undergoing R1 **pancreatic** resection compared to patients with locally advanced unresectable **pancreatic** adenocarcinoma. |
| 477 | 12673124 | Diagnosing **pancreatic** cancer using methylation specific PCR analysis of **pancreatic** juice. |
| 478 | 16979953 | **Pancreatic** cancer in patients with **pancreatic** cystic lesions: a prospective study in 197 patients. |
| 479 | 11872274 | Accelerated radiochemotherapy in **pancreatic** cancer is not necessarily related to a pathologic **pancreatic** function decline in the early period. |
| 480 | 21245863 | Poly(A) RT-PCR measurement of diagnostic genes in **pancreatic** juice in **pancreatic** cancer. |
| 481 | 21667013 | Novel monoclonal antibodies against **pancreatic** juice from **pancreatic** cancer patients and their possible application in differential diagnosis. |
| 482 | 19077468 | **Pancreatic** islet and stellate cells are the main sources of endocrine gland-derived vascular endothelial growth factorprokineticin-1 in **pancreatic** cancer. |
| 483 | 24884871 | Serum cytokine biomarker panels for discriminating **pancreatic** cancer from benign **pancreatic** disease. |
| 484 | 23966578 | Family history of diabetes and **pancreatic** cancer as risk factors for **pancreatic** cancer: the PACIFIC study. |
| 485 | 24452875 | Glucagoninsulin ratio in preoperative screening before **pancreatic** surgery: correlation with hemoglobin A1C in subjects with and without **pancreatic** cancer. |
| 486 | 16292095 | Endoscopic **pancreatic** duct stenting for relief of **pancreatic** cancer pain. |
| 487 | 21691750 | Proteomic analysis of **pancreatic** juice for the identification of biomarkers of **pancreatic** cancer. |
| 488 | 24305593 | Role of the Hypoxia-inducible factor-1 alpha induced autophagy in the conversion of non-stem **pancreatic** cancer cells into CD133+ **pancreatic** cancer stem-like cells. |
| 489 | 17616662 | The RON receptor tyrosine kinase mediates oncogenic phenotypes in **pancreatic** cancer cells and is increasingly expressed during **pancreatic** cancer progression. |
| 490 | 20049842 | Family history of cancer and risk of **pancreatic** cancer: a pooled analysis from the **Pancreatic** Cancer Cohort Consortium (PanScan). |
| 491 | 11893932 | Expression of ROCK-1 in human **pancreatic** cancer: its down-regulation by morpholino oligo antisense can reduce the migration of **pancreatic** cancer cells in vitro. |
| 492 | 17224648 | Expression of extracellular matrix metalloproteinase inducer (EMMPRINCD147) in **pancreatic** neoplasm and **pancreatic** stellate cells. |
| 493 | 20371681 | Crosstalk between mast cells and **pancreatic** cancer cells contributes to **pancreatic** tumor progression. |
| 494 | 23869654 | Effect of **pancreatic** juice cytology andor endoscopic ultrasound-guided fine-needle aspiration biopsy for **pancreatic** tumor. |
| 495 | 22523087 | Pathway analysis of genome-wide association study data highlights **pancreatic** development genes as susceptibility factors for **pancreatic** cancer. |
| 496 | 20564084 | Enhanced cell migration and invasion of CD133+ **pancreatic** cancer cells cocultured with **pancreatic** stromal cells. |
| 497 | 15788678 | Quantitative assessment of telomerase activity and human telomerase reverse transcriptase messenger RNA levels in **pancreatic** juice samples for the diagnosis of **pancreatic** cancer. |
| 498 | 22203495 | CA 19-9 in **pancreatic** cancer: retrospective evaluation of patients with suspicion of **pancreatic** cancer. |
| 499 | 10146434 | Chronic **pancreatitis** and the risk of **pancreatic** cancer: 20 year multicenter follow-up study of 1500 patients. |
| 500 | 15803824 | [Acute and chronic **pancreatitis** and **pancreatic** cancer: a chain of sequential events or independent diseases?]. |
| 501 | 15879669 | Lymphoplasmacytic sclerosing **pancreatitis** mimicking **pancreatic** cancer. |
| 502 | 10503143 | Chronic **pancreatitis** and other risk factors for **pancreatic** cancer. |
| 503 | 12755133 | [**Pancreatitis** and **pancreatic** cancer]. |
| 504 | 16223149 | [Chronic **pancreatitis**, **pancreatic** cancer and life style]. |
| 505 | 10862441 | Neuroendocrine **pancreatic** cancer: an unusual case of **pancreatitis**. |
| 506 | 10872428 | Screening for early **pancreatic** ductal adenocarcinoma in hereditary **pancreatitis**. |
| 507 | 14307949 | [**PANCREATIC** CANCER DEVELOPING AFTER **PANCREATITIS**?2 CASES]. |
| 508 | 14526127 | The race from chronic **pancreatitis** to **pancreatic** cancer. |
| 509 | 16457214 | [Chronic **pancreatitis** as a risk factor for **pancreatic** cancer]. |
| 510 | 1129110 | [Chronic **pancreatitis**, **pancreatic** cancer: differenciation by ultrasonics]. |
| 511 | 11448279 | Cigarette smoking as a risk factor for **pancreatic** cancer in patients with hereditary **pancreatitis**. |
| 512 | 15150585 | Low value of detection of KRAS2 mutations in circulating DNA to differentiate chronic **pancreatitis** to **pancreatic** cancer. |
| 513 | 15197505 | **Pancreatic** cancer or chronic **pancreatitis**? An answer from PETMRI image fusion. |
| 514 | 11770320 | [Chronic **pancreatitis** as a risk of **pancreatic** cancer]. |
| 515 | 12120218 | **Pancreatic** cancer in hereditary **pancreatitis**: consensus guidelines for prevention, screening and treatment. |
| 516 | 12145418 | Chronic **pancreatitis** and **pancreatic** cancer. |
| 517 | 17895854 | Contrast-enhanced endosonographic Doppler spectrum analysis is helpful in discrimination between focal chronic **pancreatitis** and **pancreatic** cancer. |
| 518 | 20700843 | Current research of the RAS in **pancreatitis** and **pancreatic** cancer. |
| 519 | 19432728 | Autoimmune **pancreatitis** masquerading as **pancreatic** cancer: unusual case of jaundice. |
| 520 | 18090248 | Is autoimmune **pancreatitis** a risk factor for **pancreatic** cancer? |
| 521 | 19550277 | Th1 and Th2 profiles in patients with **pancreatic** cancer compared with chronic **pancreatitis**. |
| 522 | 2102970 | Diagnosis of chronic **pancreatitis** and **pancreatic** cancer at the centre of gastroenterology. |
| 523 | 18326937 | Transversal descriptive study of xenobiotic exposures in patients with chronic **pancreatitis** and **pancreatic** cancer. |
| 524 | 19654461 | [**Pancreatitis**-associated genes and development of **pancreatic** cancer]. |
| 525 | 18418920 | [Smoking as a possible factor of chronic **pancreatitis** and **pancreatic** cancer (literature review)]. |
| 526 | 19797461 | Autoimmune **pancreatitis**: a mimic of **pancreatic** cancer. |
| 527 | 21412121 | Molecular angiogenesis profile as a tool to discriminate chronic **pancreatitis** (CP) from **pancreatic** cancer (PC). |
| 528 | 20065564 | Autoimmune **pancreatitis**, **pancreatic** cancer and immunoglobulin-G4. |
| 529 | 2293711 | Adenocarcinoma of the pancreas producing **pancreatitis** and **pancreatic** abscess. |
| 530 | 22066314 | Exenatide and sitagliptin: **pancreatitis** and **pancreatic** cancer. Harms on the rise. |
| 531 | 22372808 | Differential diagnosis between autoimmune **pancreatitis** and **pancreatic** cancer about a case. |
| 532 | 24434269 | **Pancreatic** cancer in a case of idiopathic chronic **pancreatitis**. |
| 533 | 23463371 | Glucagonlike Peptide 1-based drugs and **pancreatitis**: clarity at last, but what about **pancreatic** cancer? |
| 534 | 23622128 | Relevance of animal models of **pancreatic** cancer and **pancreatitis** to human disease. |
| 535 | 22732835 | Autoimmune **pancreatitis** (AIP) masquerading as **pancreatic** cancer: cutting is not a crime … for now. |
| 536 | 24713674 | Comment on Andersen et al, **pancreatitis**-diabetes-**pancreatic** cancer: summary of an NIDDK-NCI workshop. |
| 537 | 22766150 | EZH2 links **pancreatitis** to tissue regeneration and **pancreatic** cancer. |
| 538 | 24967284 | Imaging of focal autoimmune **pancreatitis** and differentiating it from **pancreatic** cancer. |
| 539 | 24968399 | Autoimmune **pancreatitis** mimicking **pancreatic** tumor. |
| 540 | 24979529 | Chronic **pancreatitis** and **pancreatic** cancer. |
| 541 | 8479469 | Chronic **pancreatitis** and **pancreatic** cancer. |
| 542 | 2842555 | Early diagnosis of chronic **pancreatitis** and **pancreatic** cancer. |
| 543 | 8608916 | Chronic **pancreatitis** and **pancreatic** cancer. |
| 544 | 3209862 | International Symposium on **Pancreatitis** and **Pancreatic** Cancer: Biochemical and Clinical Aspects. October 24-25, 1986, Padova, Italy. |
| 545 | 3610265 | **Pancreatic** cancer in siblings with tropical **pancreatitis**. |
| 546 | 3713123 | [2 cases of hyperosmolar coma associated with suppurative **pancreatitis** and **pancreatic** cancer]. |
| 547 | 8981517 | **Pancreatitis** and the risk of **pancreatic** cancer. |
| 548 | 3774710 | Occult **pancreatic** cancer with recurrent acute **pancreatitis**. |
| 549 | 3993032 | [Chronic **pancreatitis** and **pancreatic** cancer]. |
| 550 | 423380 | [Ultrasonically guided percutaneous fine needle aspiration biopsy of the pancreas. II. Clinical significance in the differential diagnosis of chronic **pancreatitis** and **pancreatic** cancer (author's transl)]. |
| 551 | 917560 | [Diagnostic value of endoscopic retrograde cholangiopancreatography in the differential diagnosis of **pancreatitis** and **pancreatic** cancer]. |
| 552 | 491281 | [Two cases with **pancreatic** cancer presenting as relapsing **pancreatitis** (author's transl)]. |
| 553 | 569440 | **Pancreatic** cancer presenting as **pancreatitis** of pregnancy. Case report. |
| 554 | 592515 | [Diagnostic value of various examination methods for **pancreatic** cancer--in comparison with chronic **pancreatitis** (author's transl)]. |
| 555 | 6176039 | [Value of immunological tests in the differential diagnosis of **pancreatitis** and **pancreatic** cancer]. |
| 556 | 934010 | [Surgical treatment of chronic **pancreatitis** and **pancreatic** cancer]. |
| 557 | 25354007 | [Autoimmune **pancreatitis** is a differential diagnosis to **pancreatic** cancer.] |
| 558 | 9373592 | Chronic **pancreatitis** and **pancreatic** carcinoma. |
| 559 | 6250209 | [**Pancreatic** cancer associated with **pancreatitis** and pseudocysts]. |
| 560 | 9438603 | Hereditary **pancreatitis** and familial **pancreatic** cancer. |
| 561 | 6522955 | Differential diagnosis of **pancreatic** cancer and chronic **pancreatitis** by a monoclonal antibody detecting a new cancer-associated antigen (CA 19-9). |
| 562 | 6645929 | [Acute hemorrhagic-necrotic **pancreatitis** as a principal symptom of **pancreatic** cancer]. |
| 563 | 6745623 | Chronic **pancreatitis**, **pancreatic** cancer, alcohol, and smoking. |
| 564 | 729226 | Acute **pancreatitis** and **pancreatic** cancer. |
| 565 | 7301185 | [Relationship between **pancreatic** cancer and chronic **pancreatitis**]. |
| 566 | 9762188 | [**Pancreatic** cancer or chronic **pancreatitis**?]. |
| 567 | 7441995 | [Differential diagnosis of chronic **pancreatitis** and **pancreatic** cancer (an electron microscopic study)]. |
| 568 | 7442042 | [Difficulties in the differential diagnosis of chronic **pancreatitis** and **pancreatic** cancer]. |
| 569 | 8079951 | Chronic **pancreatitis** and **pancreatic** cancer: a slow-burning wick or an explosive event? |
| 570 | 8092921 | [Segmental autotransplantation of the pancreas for **pancreatic** cancer or chronic **pancreatitis**]. |
| 571 | 8413467 | **Pancreatitis** and the risk of **pancreatic** cancer. |
| 572 | 21769291 | **Pancreatic** ductal adenocarcinoma associated with autoimmune **pancreatitis**. |
| 573 | 10570339 | EUS and K-ras analysis of pure **pancreatic** juice collected via a duodenoscope after secretin stimulation for diagnosis of **pancreatic** mass lesion: a prospective study. |
| 574 | 18154939 | Detection of human telomerase reverse transcriptase (hTERT) expression in tissue and **pancreatic** juice from **pancreatic** cancer. |
| 575 | 24025713 | Targeting of NAD metabolism in **pancreatic** cancer cells: potential novel therapy for **pancreatic** tumors. |
| 576 | 18725222 | Murine embryonic stem cell-derived **pancreatic** acinar cells recapitulate features of early **pancreatic** differentiation. |
| 577 | 10353750 | Detection of mutations of p53 tumor suppressor gene in **pancreatic** juice and its application to diagnosis of patients with **pancreatic** cancer: comparison with K-ras mutation. |
| 578 | 16052519 | Quantitative analysis of MUC1 and MUC5AC mRNA in **pancreatic** juice for preoperative diagnosis of **pancreatic** cancer. |
| 579 | 11385254 | Prevention of **pancreatic** cancer and strategies for management of familial **pancreatic** cancer. |
| 580 | 19425054 | An integrated humoral and cellular response is elicited in **pancreatic** cancer by alpha-enolase, a novel **pancreatic** ductal adenocarcinoma-associated antigen. |
| 581 | 22361500 | Autoimmune **pancreatitis** mimicking **pancreatic** cancer. |
| 582 | 24290142 | Comparison of F-18-FDG PETCT findings between **pancreatic** solid pseudopapillary tumor and **pancreatic** ductal adenocarcinoma. |
| 583 | 23397095 | **Pancreatic** cancer and supportive care--**pancreatic** exocrine insufficiency negatively impacts on quality of life. |
| 584 | 24856668 | Extended pancreatectomy in **pancreatic** ductal adenocarcinoma: definition and consensus of the International Study Group for **Pancreatic** Surgery (ISGPS). |
| 585 | 6305473 | Assessment of weight loss, food intake, fat metabolism, malabsorption, and treatment of **pancreatic** insufficiency in **pancreatic** cancer. |
| 586 | 21088419 | Is early diagnosis of **pancreatic** cancer fiction? Surveillance of individuals at high risk for **pancreatic** cancer. |
| 587 | 1860169 | Activation of c-K-ras is frequent in **pancreatic** carcinomas of Syrian hamsters, but is absent in **pancreatic** tumors of rats. |
| 588 | 22504379 | Value of cytodiagnosis using endoscopic nasopancreatic drainage for early diagnosis of **pancreatic** cancer: establishing a new method for the early detection of **pancreatic** carcinoma in situ. |
| 589 | 10503150 | The role of endoscopy in acute recurrent and chronic **pancreatitis** and **pancreatic** cancer. |
| 590 | 16713745 | Do cytokine concentrations in **pancreatic** juice predict the presence of **pancreatic** diseases? |
| 591 | 11522750 | Biglycan is overexpressed in **pancreatic** cancer and induces G1-arrest in **pancreatic** cancer cell lines. |
| 592 | 12065097 | Increased expression of heterogeneous nuclear ribonucleoprotein A2B1 (hnRNP) in **pancreatic** tissue from smokers and **pancreatic** tumor cells. |
| 593 | 17236203 | Twist, a novel oncogene, is upregulated in **pancreatic** cancer: clinical implication of Twist expression in **pancreatic** juice. |
| 594 | 25354268 | **Pancreatic** cancer immunotherapy using a tumor lysate vaccine, engineered to express α-gal epitopes, targets **pancreatic** cancer stem cells. |
| 595 | 25760059 | Overexpression of serinethreonine‑protein kinase-1 in **pancreatic** cancer tissue: Serinethreonine-protein kinase-1 knockdown increases the chemosensitivity of **pancreatic** cancer cells. |
| 596 | 12358243 | Quantitative analysis of K-ras gene mutation in **pancreatic** tissue obtained by endoscopic ultrasonography-guided fine needle aspiration: clinical utility for diagnosis of **pancreatic** tumor. |
| 597 | 16391791 | Analysis of the invasion-metastasis mechanism in **pancreatic** cancer: involvement of plasmin(ogen) cascade proteins in the invasion of **pancreatic** cancer cells. |
| 598 | 3970548 | [Early diagnosis of **pancreatic** cancer by routine CT scanning; significance of the **pancreatic** bile duct scanning]. |
| 599 | 25725585 | Apoptosis and anergy of T cell induced by **pancreatic** stellate cells-derived galectin-1 in **pancreatic** cancer. |
| 600 | 15246966 | Inflammation and Cancer V. Chronic **pancreatitis** and **pancreatic** cancer. |
| 601 | 25057164 | Diabetes, antidiabetic medications, and **pancreatic** cancer risk: an analysis from the International **Pancreatic** Cancer Case-Control Consortium. |
| 602 | 8792713 | Detection of K-ras point mutation at codon 12 in pure **pancreatic** juice collected 3 years and 6 months before the clinical diagnosis of **pancreatic** cancer. |
| 603 | 25347153 | Proteins associated with **pancreatic** cancer survival in patients with resectable **pancreatic** ductal adenocarcinoma. |
| 604 | 24856119 | Borderline resectable **pancreatic** cancer: a consensus statement by the International Study Group of **Pancreatic** Surgery (ISGPS). |
| 605 | 24189457 | Efficient targeting and tumor retardation effect of **pancreatic** adenocarcinoma up-regulated factor (PAUF)-specific RNA replacement in **pancreatic** cancer mouse model. |
| 606 | 15867264 | Expression of HOXB2, a retinoic acid signaling target in **pancreatic** cancer and **pancreatic** intraepithelial neoplasia. |
| 607 | 24111684 | **Pancreatic** intraepithelial neoplasia and histological changes in non-neoplastic pancreas associated with neoadjuvant therapy in patients with **pancreatic** ductal adenocarcinoma. |
| 608 | 20458087 | Anthropometric measures, body mass index, and **pancreatic** cancer: a pooled analysis from the **Pancreatic** Cancer Cohort Consortium (PanScan). |
| 609 | 25557640 | Interaction between **pancreatic** cancer cells and tumor-associated macrophages promotes the invasion of **pancreatic** cancer cells and the differentiation and migration of macrophages. |
| 610 | 18373165 | Ultrasound-guided percutaneous **pancreatic** tumor biopsy in **pancreatic** cancer: a comparison with metastatic liver tumor biopsy, including sensitivity, specificity, and complications. |
| 611 | 10464348 | Endoscopic **pancreatic** stenting in **pancreatic** cancer. |
| 612 | 15753353 | Gene expression profiles in **pancreatic** intraepithelial neoplasia reflect the effects of Hedgehog signaling on **pancreatic** ductal epithelial cells. |
| 613 | 19276868 | Identification and characterization of a novel anticancer agent with selectivity against deleted in **pancreatic** cancer locus 4 (DPC4)-deficient **pancreatic** and colon cancer cells. |
| 614 | 18404646 | Geminin is overexpressed in human **pancreatic** cancer and downregulated by the bioflavanoid apigenin in **pancreatic** cancer cell lines. |
| 615 | 6627206 | Significance of carcinoembryonic antigen levels and cytology of pure **pancreatic** juice in diagnosis of **pancreatic** cancer. |
| 616 | 25502147 | CCL18 promotes epithelial-mesenchymal transition, invasion and migration of **pancreatic** cancer cells in **pancreatic** ductal adenocarcinoma. |
| 617 | 19687327 | **Pancreatic** proteolytic enzyme therapy compared with gemcitabine-based chemotherapy for the treatment of **pancreatic** cancer. |
| 618 | 19690548 | A randomised phase III trial comparing gemcitabine with surgery-only in patients with resected **pancreatic** cancer: Japanese Study Group of Adjuvant Therapy for **Pancreatic** Cancer. |
| 619 | 7598300 | Identification of K-ras mutations in **pancreatic** juice in the early diagnosis of **pancreatic** cancer. |
| 620 | 18803764 | Immunoglobulin E antibodies from **pancreatic** cancer patients mediate antibody-dependent cell-mediated cytotoxicity against **pancreatic** cancer cells. |
| 621 | 22963768 | Comparative testing of various **pancreatic** cancer stem cells results in a novel class of **pancreatic**-cancer-initiating cells. |
| 622 | 22323115 | Transient receptor potential melastatin-related 7 channel is overexpressed in human **pancreatic** ductal adenocarcinomas and regulates human **pancreatic** cancer cell migration. |
| 623 | 17416753 | S100A6 is increased in a stepwise manner during **pancreatic** carcinogenesis: clinical value of expression analysis in 98 **pancreatic** juice samples. |
| 624 | 21571068 | Functional analysis of a **pancreatic** secretory trypsin inhibitor-like protein in insects: silencing effects resemble the human **pancreatic** autodigestion phenotype. |
| 625 | 23552468 | The 'N-factors' in **pancreatic** cancer: functional relevance of NF-κB, NFAT and Nrf2 in **pancreatic** cancer. |
| 626 | 11900678 | Differentiating **pancreatic** cancer from pseudotumorous chronic **pancreatitis**. |
| 627 | 23200980 | Mutant TP53 in duodenal samples of **pancreatic** juice from patients with **pancreatic** cancer or high-grade dysplasia. |
| 628 | 17227797 | **Pancreatic** stellate cells are an important source of MMP-2 in human **pancreatic** cancer and accelerate tumor progression in a murine xenograft model and CAM assay. |
| 629 | 21472101 | Proteomic analysis of **pancreatic** intraepithelial neoplasia and **pancreatic** carcinoma in rat models. |
| 630 | 10372940 | Autoimmune **pancreatitis**, **pancreatic** mass, and lower gastrointestinal bleed. |
| 631 | 15851011 | mda-7IL24 kills **pancreatic** cancer cells by inhibition of the WntPI3K signaling pathways: identification of IL-20 receptor-mediated bystander activity against **pancreatic** cancer. |
| 632 | 20971884 | Variant ABO blood group alleles, secretor status, and risk of **pancreatic** cancer: results from the **pancreatic** cancer cohort consortium. |
| 633 | 9211490 | c-met expression in **pancreatic** cancer and effects of hepatocyte growth factor on **pancreatic** cancer cell growth. |
| 634 | 24077287 | Epithelial splicing regulatory protein 1 is a favorable prognostic factor in **pancreatic** cancer that attenuates **pancreatic** metastases. |
| 635 | 22497024 | Randomized clinical trial of external stent drainage of the **pancreatic** duct to reduce postoperative **pancreatic** fistula after pancreaticojejunostomy. |
| 636 | 8641983 | Detection of K-ras point mutations at codon 12 in **pancreatic** juice for the diagnosis of **pancreatic** cancer by hybridization protection assay: a simple method for the determination of the types of point mutation. |
| 637 | 19106745 | Inhibition of **pancreatic** cancer cell proliferation by propranolol occurs through apoptosis induction: the study of beta-adrenoceptor antagonist's anticancer effect in **pancreatic** cancer cell. |
| 638 | 6881993 | [**Pancreatic** cancer-related antigen (PCAA) and **pancreatic** tissue antigen (PaA)--their chemical compositions, tissue localization, and clinical significance]. |
| 639 | 11206832 | The impact of laparoscopic biopsy of **pancreatic** lymph nodes with helium and carbon dioxide on port site and liver metastasis in BOP-induced **pancreatic** cancer in hamster. |
| 640 | 18097568 | Characterisation of a novel matrix metalloproteinase inhibitor on **pancreatic** adenocarcinoma cells in vitro and in an orthotopic **pancreatic** cancer model in vivo. |
| 641 | 20395845 | Nerve growth factor and artemin are paracrine mediators of **pancreatic** neuropathy in **pancreatic** adenocarcinoma. |
| 642 | 17303479 | Plasma proteomics of **pancreatic** cancer patients by multi-dimensional liquid chromatography and two-dimensional difference gel electrophoresis (2D-DIGE): up-regulation of leucine-rich alpha-2-glycoprotein in **pancreatic** cancer. |
| 643 | 18316392 | Proliferation of colo-357 **pancreatic** carcinoma cells and survival of patients with **pancreatic** carcinoma are not altered by insulin glargine. |
| 644 | 24075515 | Simvastatin delay progression of **pancreatic** intraepithelial neoplasia and cancer formation in a genetically engineered mouse model of **pancreatic** cancer. |
| 645 | 23904839 | From Acute **Pancreatitis** to Stage IV **Pancreatic** Cancer in 12 Weeks. |
| 646 | 22811080 | Human **pancreatic** cancer fusion 2 (HPC2) 1-B3: a novel monoclonal antibody to screen for **pancreatic** ductal dysplasia. |
| 647 | 22188668 | **Pancreatic** cancer cells and normal **pancreatic** duct epithelial cells express an autocrine catecholamine loop that is activated by nicotinic acetylcholine receptors α3, α5, and α7. |
| 648 | 16186663 | Is zinc concentration in **pancreatic** fluid a marker for **pancreatic** diseases? |
| 649 | 24366368 | Enteric duplication cyst of the pancreas associated with chronic **pancreatitis** and **pancreatic** cancer. |
| 650 | 654297 | [Endoscopic retrograde cholangiopancreatography in the differential diagnosis of chronic **pancreatitis** and **pancreatic** cancer (author's transl)]. |
| 651 | 23475261 | Serine protease inhibitor Kazal type 1 and epidermal growth factor receptor are expressed in **pancreatic** tubular adenocarcinoma, intraductal papillary mucinous neoplasm, and **pancreatic** intraepithelial neoplasia. |
| 652 | 23924790 | Evaluation of ipilimumab in combination with allogeneic **pancreatic** tumor cells transfected with a GM-CSF gene in previously treated **pancreatic** cancer. |
| 653 | 21403843 | Detection of precursor lesions of **pancreatic** adenocarcinoma in PET-CT in a genetically engineered mouse model of **pancreatic** cancer. |
| 654 | 10415865 | Hereditary **pancreatitis** and **pancreatic** carcinoma. |
| 655 | 2800494 | [Laboratory diagnosis of **pancreatitis** and **pancreatic** cancer]. |
| 656 | 3859414 | CA 19-9 in the differential diagnosis between **pancreatic** cancer and chronic **pancreatitis**. |
| 657 | 20510833 | **Pancreatic** enzyme replacement therapy in chronic **pancreatitis**. |
| 658 | 9051867 | Etiologic links between chronic **pancreatitis** and **pancreatic** cancer. |
| 659 | 19208745 | KRAS2 mutations in human **pancreatic** acinar-ductal metaplastic lesions are limited to those with PanIN: implications for the human **pancreatic** cancer cell of origin. |
| 660 | 12409332 | Low sensitivity of the ki-ras polymerase chain reaction for diagnosing **pancreatic** cancer from **pancreatic** juice and bile: a multicenter prospective trial. |
| 661 | 9168704 | Local staging of **pancreatic** cancer: criteria for unresectability of major vessels as revealed by **pancreatic**-phase, thin-section helical CT. |
| 662 | 21994333 | Variations of oral microbiota are associated with **pancreatic** diseases including **pancreatic** cancer. |
| 663 | 2730018 | [Diagnostic usefulness and limitation of measuring **pancreatic** cancer associate antigen, SPan-1, in patients with **pancreatic** cancer]. |
| 664 | 8012983 | Detection of K-ras mutations in the stool of patients with **pancreatic** adenocarcinoma and **pancreatic** ductal hyperplasia. |
| 665 | 21196815 | **Pancreatic** adenocarcinoma up-regulated factor (PAUF) enhances the expression of β-catenin, leading to a rapid proliferation of **pancreatic** cells. |
| 666 | 23179793 | Multiple small "imaging" branch-duct type intraductal papillary mucinous neoplasms (IPMNs) in familial **pancreatic** cancer: indicator for concomitant high grade **pancreatic** intraepithelial neoplasia? |
| 667 | 22791813 | Effects of chronic nicotine on the autocrine regulation of **pancreatic** cancer cells and **pancreatic** duct epithelial cells by stimulatory and inhibitory neurotransmitters. |
| 668 | 1553375 | Pancreas divisum with early **pancreatic** cancer--presenting as chronic obstructive **pancreatitis**. |
| 669 | 17067749 | **Pancreatic** cancer mortality in Egypt: comparison to the United States **pancreatic** cancer mortality rates. |
| 670 | 25528826 | [Persistence of chronic inflammatory responses, role in the development of chronic **pancreatitis**, obesity and **pancreatic** cancer]. |
| 671 | 22844642 | Vascularisation pattern of chronic **pancreatitis** compared with **pancreatic** carcinoma: results from contrast-enhanced endoscopic ultrasound. |
| 672 | 20657174 | Werner syndrome as a hereditary risk factor for exocrine **pancreatic** cancer: potential role of WRN in **pancreatic** tumorigenesis and patient-tailored therapy. |
| 673 | 24828013 | Quantitative assessment of the diagnostic role of human telomerase activity from **pancreatic** juice in **pancreatic** cancer. |
| 674 | 1668625 | [Differential diagnosis of cholestasis in **pancreatic** cancer and chronic **pancreatitis**]. |
| 675 | 17018631 | Identification of an agent selectively targeting DPC4 (deleted in **pancreatic** cancer locus 4)-deficient **pancreatic** cancer cells. |
| 676 | 23847240 | Hyperglycemia, insulin resistance, impaired **pancreatic** β-cell function, and risk of **pancreatic** cancer. |
| 677 | 18493433 | Diagnostic evaluation of **pancreatic** carcinoma and chronic **pancreatitis** by pancreatoscopy. |
| 678 | 10228761 | Diagnosis of **pancreatic** cancer by cytology and measurement of oncogene and tumor markers in pure **pancreatic** juice aspirated by endoscopy. |
| 679 | 25393586 | Mutant KRAS and GNAS DNA Concentrations in Secretin-Stimulated **Pancreatic** Fluid Collected from the **Pancreatic** Duct and the Duodenal Lumen. |
| 680 | 23981573 | Chronic **pancreatitis**: a path to **pancreatic** cancer. |
| 681 | 20484960 | Pre-diagnostic levels of anionic trypsinogen, cationic trypsinogen, and **pancreatic** secretory trypsin inhibitor in relation to **pancreatic** cancer risk. |
| 682 | 18239323 | **Pancreatic** cancer associated with autoimmune **pancreatitis** in remission. |
| 683 | 10454945 | Early diagnosis and treatment of **pancreatic** dysplasia in patients with a family history of **pancreatic** cancer. |
| 684 | 19755388 | Down-regulation of ZIP4 by RNA interference inhibits **pancreatic** cancer growth and increases the survival of nude mice with **pancreatic** cancer xenografts. |
| 685 | 25684468 | Establishment of a **pancreatic** cancer stem cell model using the SW1990 human **pancreatic** cancer cell line in nude mice. |
| 686 | 24595374 | **Pancreatic** satellite cells derived galectin-1 increase the progression and less survival of **pancreatic** ductal adenocarcinoma. |
| 687 | 19292977 | Acute **pancreatitis** markedly accelerates **pancreatic** cancer progression in mice expressing oncogenic Kras. |
| 688 | 2486016 | [Chronic **pancreatitis** and **pancreatic** cancer. An often difficult differential diagnosis]. |
| 689 | 11711770 | Clinical usefulness of K-ras gene mutation detection and cytology in **pancreatic** juice in the diagnosis and screening of **pancreatic** cancer. |
| 690 | 21054984 | [Establish a gemcitabine-resistant **pancreatic** cancer cell line SW1990GZ and research the relationship between SW1990GZ and **pancreatic** cancer stem cell]. |
| 691 | 21780106 | High expression of Galectin-1 in **pancreatic** stellate cells plays a role in the development and maintenance of an immunosuppressive microenvironment in **pancreatic** cancer. |
| 692 | 10211418 | A prospective multicenter trial evaluating diagnostic validity of multivariate analysis and individual serum marker in differential diagnosis of **pancreatic** cancer from benign **pancreatic** diseases. |
| 693 | 9802880 | The cell-surface heparan sulfate proteoglycan glypican-1 regulates growth factor action in **pancreatic** carcinoma cells and is overexpressed in human **pancreatic** cancer. |
| 694 | 6687017 | Potentiation of **pancreatic** carcinogenesis in the rat by DL-ethionine-induced **pancreatitis**. |
| 695 | 17344598 | [Expression of KiSS-1mRNA in **pancreatic** ductal adenocarcinoma and non-cancerous **pancreatic** tissues in SD rats]. |
| 696 | 24566656 | [Autoimmune **pancreatitis** mimicking **pancreatic** tumor]. |
| 697 | 11798705 | [K-ras mutation in **pancreatic** juice from patients with **pancreatic** carcinoma]. |
| 698 | 9821175 | Quantitative determination of K-ras mutations in **pancreatic** juice for diagnosis of **pancreatic** cancer using hybridization protection assay. |
| 699 | 17507610 | Evaluation of matrix metalloproteinase 7 in plasma and **pancreatic** juice as a biomarker for **pancreatic** cancer. |
| 700 | 24278292 | Novel **pancreatic** cancer cell lines derived from genetically engineered mouse models of spontaneous **pancreatic** adenocarcinoma: applications in diagnosis and therapy. |
| 701 | 21455033 | Inactivation of Brca2 cooperates with Trp53(R172H) to induce invasive **pancreatic** ductal adenocarcinomas in mice: a mouse model of familial **pancreatic** cancer. |
| 702 | 20183795 | Morphometric studies in human **pancreatic** cancer argues against the etiological role of type 2 diabetes in **pancreatic** cancer. |
| 703 | 22640743 | SIRT1 inhibits proliferation of **pancreatic** cancer cells expressing **pancreatic** adenocarcinoma up-regulated factor (PAUF), a novel oncogene, by suppression of β-catenin. |
| 704 | 7797131 | Detection of c-Ki-ras gene codon 12 mutations from **pancreatic** duct brushings in the diagnosis of **pancreatic** tumours. |
| 705 | 24600409 | **Pancreatic** cancer risk in hereditary **pancreatitis**. |
| 706 | 22815775 | Evaluation of a gene-directed enzyme-product therapy (GDEPT) in human **pancreatic** tumor cells and their use as in vivo models for **pancreatic** cancer. |
| 707 | 21109934 | Overexpression of fibroblast growth factor receptor 4 in high-grade **pancreatic** intraepithelial neoplasia and **pancreatic** ductal adenocarcinoma. |
| 708 | 20428826 | Identification of **pancreatic** juice proteins as biomarkers of **pancreatic** cancer. |
| 709 | 19435915 | Up-regulation of L1CAM in **pancreatic** duct cells is transforming growth factor beta1- and slug-dependent: role in malignant transformation of **pancreatic** cancer. |
| 710 | 22517435 | Serum autoantibodies to **pancreatic** cancer antigens as biomarkers of **pancreatic** cancer in a San Francisco Bay Area case-control study. |
| 711 | 20005738 | The expression of IL-8 and IL-8 receptors in **pancreatic** adenocarcinomas and **pancreatic** neuroendocrine tumours. |
| 712 | 22605658 | Development and validation of a symptom index for advanced hepatobiliary and **pancreatic** cancers: the National Comprehensive Cancer Network Functional Assessment of Cancer Therapy (NCCN-FACT) Hepatobiliary-**Pancreatic** Symptom Index (NFHSI). |
| 713 | 16818649 | Pim-3, a proto-oncogene with serinethreonine kinase activity, is aberrantly expressed in human **pancreatic** cancer and phosphorylates bad to block bad-mediated apoptosis in human **pancreatic** cancer cell lines. |
| 714 | 24876152 | The role of the hepatocyte growth factorc-MET pathway in **pancreatic** stellate cell-endothelial cell interactions: antiangiogenic implications in **pancreatic** cancer. |
| 715 | 21667806 | **Pancreatic** adenocarcinoma presenting as acute **pancreatitis** during pregnancy: clinical and radiologic manifestations. |
| 716 | 24981695 | [Pain in chronic **pancreatitis** and **pancreatic** cancer - treatment options]. |
| 717 | 4082285 | Serum deoxyribonuclease and ribonuclease in **pancreatic** cancer and chronic **pancreatitis**. |
| 718 | 20660600 | A combination of DR5 agonistic monoclonal antibody with gemcitabine targets **pancreatic** cancer stem cells and results in long-term disease control in human **pancreatic** cancer model. |
| 719 | 21386640 | Exposure during **pancreatic** surgery. Do we have to examine the falciform and round ligaments for **pancreatic** cancer metastasis? |
| 720 | 21590256 | Analysis of K-ras gene mutations in human **pancreatic** cancer cell lines and in bile samples from patients with **pancreatic** and biliary cancers. |
| 721 | 25175624 | Plasma carotenoids, vitamin C, retinol and tocopherols levels and **pancreatic** cancer risk within the European Prospective Investigation into Cancer and Nutrition: a nested case-control study: plasma micronutrients and **pancreatic** cancer risk. |
| 722 | 15686632 | Chronic **pancreatitis**, **pancreatic** adenocarcinoma and the black box in-between. |
| 723 | 23494612 | **Pancreatic** cancer causing acute **pancreatitis**: a comparative study with cancer patients without **pancreatitis** and **pancreatitis** patients without cancer. |
| 724 | 9094151 | Clinical evaluation and safety of loxiglumide (CCK-A receptor antagonist) in nonresectable **pancreatic** cancer patients. Italian **Pancreatic** Cancer Study Group. |
| 725 | 17533083 | Pain in chronic **pancreatitis** and **pancreatic** cancer. |
| 726 | 18706098 | Proteomic analysis identifies MMP-9, DJ-1 and A1BG as overexpressed proteins in **pancreatic** juice from **pancreatic** ductal adenocarcinoma patients. |
| 727 | 19277793 | Intraoperative assessment of **pancreatic** neck margin at the time of pancreaticoduodenectomy increases likelihood of margin-negative resection in patients with **pancreatic** cancer. |
| 728 | 9456949 | Small peripancreatic veins: improved assessment in **pancreatic** cancer patients using thin-section **pancreatic** phase helical CT. |
| 729 | 9696930 | Genetic polymorphism of N-acetyltransferases, glutathione S-transferase M1 and NAD(P)H:quinone oxidoreductase in relation to malignant and benign **pancreatic** disease risk. The International **Pancreatic** Disease Study Group. |
| 730 | 17226905 | Detection of disseminated **pancreatic** cells by amplification of cytokeratin-19 with quantitative RT-PCR in blood, bone marrow and peritoneal lavage of **pancreatic** carcinoma patients. |
| 731 | 20068108 | Interleukin 13 mediates signal transduction through interleukin 13 receptor alpha2 in **pancreatic** ductal adenocarcinoma: role of IL-13 Pseudomonas exotoxin in **pancreatic** cancer therapy. |
| 732 | 22859495 | Mutant GNAS detected in duodenal collections of secretin-stimulated **pancreatic** juice indicates the presence or emergence of **pancreatic** cysts. |
| 733 | 22496584 | In vivo SPECT imaging with 111In-DOTA-c(RGDfK) to detect early **pancreatic** cancer in a hamster **pancreatic** carcinogenesis model. |
| 734 | 15958547 | In vitro modeling of human **pancreatic** duct epithelial cell transformation defines gene expression changes induced by K-ras oncogenic activation in **pancreatic** carcinogenesis. |
| 735 | 21272935 | **Pancreatic** cancer bears overexpression of neurotensin and neurotensin receptor subtype-1 and SR 48692 counteracts neurotensin induced cell proliferation in human **pancreatic** ductal carcinoma cell line PANC-1. |
| 736 | 18617777 | Expression of COX-2 is associated with accumulation of p53 in **pancreatic** cancer: analysis of COX-2 and p53 expression in premalignant and malignant ductal **pancreatic** lesions. |
| 737 | 24737347 | BRCA1 and BRCA2 germline mutations are frequently demonstrated in both high-risk **pancreatic** cancer screening and **pancreatic** cancer cohorts. |
| 738 | 16532285 | Increased levels of NAD(P)H: quinone oxidoreductase 1 (NQO1) in **pancreatic** tissues from smokers and **pancreatic** adenocarcinomas: A potential biomarker of early damage in the pancreas. |
| 739 | 19302292 | **Pancreatic** adenocarcinoma up-regulated factor (PAUF), a novel up-regulated secretory protein in **pancreatic** ductal adenocarcinoma. |
| 740 | 11385249 | Differentiation of chronic **pancreatitis** from **pancreatic** cancer: recent advances in molecular diagnosis. |
| 741 | 18791122 | Recent progress on normal and malignant **pancreatic** stemprogenitor cell research: therapeutic implications for the treatment of type 1 or 2 diabetes mellitus and aggressive **pancreatic** cancer. |
| 742 | 21653254 | Integrated proteomic profiling of cell line conditioned media and **pancreatic** juice for the identification of **pancreatic** cancer biomarkers. |
| 743 | 23890140 | Role of adipocytokines and its correlation with endocrine **pancreatic** function in patients with **pancreatic** cancer. |
| 744 | 20818121 | **Pancreatic** actinomycosis as a cause of retroperitoneal fibrosis in a patient with chronic **pancreatitis**. Case report and literature review. |
| 745 | 12598343 | Treatment of **pancreatic** cancer with a combination of irinotecan (CPT-11) and gemcitabine: a multicenter phase II study by the Greek Cooperative Group for **Pancreatic** Cancer. |
| 746 | 25117591 | Incidental **pancreatic** cystic lesions: is there a relationship with the development of **pancreatic** adenocarcinoma and all-cause mortality? |
| 747 | 16269378 | Current practice patterns in **pancreatic** surgery: results of a multi-institutional analysis of seven large surgical departments in Germany with 1454 **pancreatic** head resections, 1999 to 2004 (German Advanced Surgical Treatment study group). |
| 748 | 22022519 | Recruitment and activation of **pancreatic** stellate cells from the bone marrow in **pancreatic** cancer: a model of tumor-host interaction. |
| 749 | 21276229 | In vivo imaging of **pancreatic** tumours and liver metastases using 7 Tesla MRI in a murine orthotopic **pancreatic** cancer model and a liver metastases model. |
| 750 | 24250822 | Clinical significance and revisiting the meaning of CA 19-9 blood level before and after the treatment of **pancreatic** ductal adenocarcinoma: analysis of 1,446 patients from the **pancreatic** cancer cohort in a single institution. |
| 751 | 23828595 | Calorie restriction delays the progression of lesions to **pancreatic** cancer in the LSL-KrasG12D; Pdx-1Cre mouse model of **pancreatic** cancer. |
| 752 | 19749458 | **Pancreatic** carcinogenesis: The impact of chronic **pancreatitis** and its clinical relevance. |
| 753 | 18575732 | Adoptive immunotherapy for **pancreatic** cancer: cytotoxic T lymphocytes stimulated by the MUC1-expressing human **pancreatic** cancer cell line YPK-1. |
| 754 | 25120674 | Chemotherapy-induced fulminant acute **pancreatitis** in **pancreatic** carcinoma: A case report. |
| 755 | 23300742 | CD271⁺ subpopulation of **pancreatic** stellate cells correlates with prognosis of **pancreatic** cancer and is regulated by interaction with cancer cells. |
| 756 | 23622129 | Inflammation, autophagy, and obesity: common features in the pathogenesis of **pancreatitis** and **pancreatic** cancer. |
| 757 | 742614 | Angiography in chronic **pancreatitis** and **pancreatic** cancer. A critical evaluation. |
| 758 | 10829519 | Chronic **pancreatitis**: relation to acute **pancreatitis** and **pancreatic** cancer. |
| 759 | 25050737 | Rottlerin suppresses growth of human **pancreatic** tumors in nude mice, and **pancreatic** cancer cells isolated from Kras(G12D) mice. |
| 760 | 16211233 | Serum amyloid A as a tumor marker in sera of nude mice with orthotopic human **pancreatic** cancer and in plasma of patients with **pancreatic** cancer. |
| 761 | 24502441 | Expression of DNA-repair proteins and their significance in **pancreatic** cancer and non-cancerous **pancreatic** tissues of Sprague-Dawley rats. |
| 762 | 25259922 | Vitamin D receptor-mediated stromal reprogramming suppresses **pancreatitis** and enhances **pancreatic** cancer therapy. |
| 763 | 15367883 | Desmoplastic reaction in **pancreatic** cancer: role of **pancreatic** stellate cells. |
| 764 | 24570946 | Histamine regulation of **pancreatitis** and **pancreatic** cancer: a review of recent findings. |
| 765 | 17034997 | Matrix metalloproteinase inhibitor RO 28-2653 decreases liver metastasis by reduction of MMP-2 and MMP-9 concentration in BOP-induced ductal **pancreatic** cancer in Syrian Hamsters: inhibition of matrix metalloproteinases in **pancreatic** cancer. |
| 766 | 25178600 | Chronic calcific **pancreatitis** and **pancreatic** cancer. |
| 767 | 12649567 | N34S, a **pancreatitis** associated SPINK1 mutation, is not associated with sporadic **pancreatic** cancer. |
| 768 | 1561055 | [CT findings of mucin-producing **pancreatic** cancer--differentiation from chronic **pancreatitis**]. |
| 769 | 19896101 | Distinguishing **pancreatic** cancer from autoimmune **pancreatitis**: a comparison of two strategies. |
| 770 | 21909380 | Targeting epigenetic regulation of miR-34a for treatment of **pancreatic** cancer by inhibition of **pancreatic** cancer stem cells. |
| 771 | 8513440 | p53 mutations are common in **pancreatic** cancer and are absent in chronic **pancreatitis**. |
| 772 | 25000673 | Two different types of diabetes mellitus in **pancreatic** cancer population. Comparative study between new onset and long standing diabetes mellitus on 76 patients with **pancreatic** cancer. |
| 773 | 17323023 | [Autoimmune **pancreatitis**--a rare and difficult differential diagnosis to **pancreatic** cancer]. |
| 774 | 19896097 | Ras activity in acinar cells links chronic **pancreatitis** and **pancreatic** cancer. |
| 775 | 21665147 | **Pancreatitis**-induced inflammation contributes to **pancreatic** cancer by inhibiting oncogene-induced senescence. |
| 776 | 22423237 | Histopathologically proven autoimmune **pancreatitis** mimicking neuroendocrine tumor or **pancreatic** cancer. |
| 777 | 20455050 | [Chronic **pancreatitis** as a risk factor for the development of **pancreatic** cancer--diagnostic challenges]. |
| 778 | 18392050 | Early diagnosis of **pancreatic** cancer: neutrophil gelatinase-associated lipocalin as a marker of **pancreatic** intraepithelial neoplasia. |
| 779 | 18346883 | **Pancreatic** cancer within a UK cancer network with special emphasis on locally advanced non-metastatic **pancreatic** cancer. |
| 780 | 19260744 | Chronic **pancreatitis** and the differential diagnosis versus **pancreatic** cancer. |
| 781 | 1651876 | Labelled antibody imaging in **pancreatic** cancer, cholangiocarcinoma, chronic **pancreatitis** and sclerosing cholangitis. |
| 782 | 15550766 | Cell death pathways in **pancreatitis** and **pancreatic** cancer. |
| 783 | 25516685 | Focal autoimmune **pancreatitis** and chronic sclerosing sialadenitis mimicking **pancreatic** cancer and neck metastasis. |
| 784 | 16556492 | Impact of polyunsaturated fatty acids on hepato-**pancreatic** prostaglandin and leukotriene concentration in ductal **pancreatic** cancer -- is there a correlation to tumour growth and liver metastasis? |
| 785 | 6640255 | Coeliac plexus block for pain in **pancreatic** cancer and chronic **pancreatitis**. |
| 786 | 11727090 | The impact of laparoscopic biopsy of **pancreatic** lymph nodes on lipid peroxidation using helium and carbon dioxide in BOP-induced **pancreatic** cancer in hamsters. |
| 787 | 15932149 | [Epidemiological and molecular-genetic aspects of the association between chronic **pancreatitis** and **pancreatic** cancer]. |
| 788 | 20424980 | Distinguishing **pancreatic** cancer from autoimmune **pancreatitis**. |
| 789 | 20460943 | Primers on molecular pathways: lipopolysaccharide signaling - potential role in **pancreatitis** and **pancreatic** cancer. |
| 790 | 24694877 | Embelin suppresses growth of human **pancreatic** cancer xenografts, and **pancreatic** cancer cells isolated from KrasG12D mice by inhibiting Akt and Sonic hedgehog pathways. |
| 791 | 11746913 | Serum protein profiles of patients with **pancreatic** cancer and chronic **pancreatitis**: searching for a diagnostic protein pattern. |
| 792 | 19734641 | Ascaris lumbricoides-induced acute **pancreatitis**: diagnosis during EUS for a suspected small **pancreatic** tumor. |
| 793 | 23294585 | Clinical Value of Dual-energy CT in Detection of **Pancreatic** Adenocarcinoma: Investigation of the Best **Pancreatic** Tumor Contrast to Noise Ratio. |
| 794 | 4092355 | Copper, zinc and copperzinc ratio in chronic **pancreatitis** and **pancreatic** cancer. |
| 795 | 23291204 | [**Pancreatic** cancer or autoimmune **pancreatitis**: endosonography as a diagnostic reviser]. |
| 796 | 12783204 | Peptides designed from molecular modeling studies of the ras-p21 protein induce phenotypic reversion of a **pancreatic** carcinoma cell line but have no effect on normal **pancreatic** acinar cell growth. |
| 797 | 1300550 | [Value of plasma testosterone, carcinoembryonic antigen and CA 19-9 in the differential diagnosis of **pancreatic** carcinoma and chronic **pancreatitis**]. |
| 798 | 11297271 | Increased risk of incident **pancreatic** cancer among first-degree relatives of patients with familial **pancreatic** cancer. |
| 799 | 2297660 | Role of endoscopic retrograde cholangiopancreatography in differentiating **pancreatic** cancer coexisting with chronic **pancreatitis**. |
| 800 | 12134623 | **Pancreatitis** as a risk for **pancreatic** cancer. |
| 801 | 8532652 | Quantitative analysis of collagen and collagen subtypes I, III, and V in human **pancreatic** cancer, tumor-associated chronic **pancreatitis**, and alcoholic chronic **pancreatitis**. |
| 802 | 15942268 | History of the European **pancreatic** club: the first 40 years 1965-2005. The development of the European **pancreatic** club as a scientific society. |
| 803 | 9427770 | Effects of lithium gammalinolenate on the perfusion of liver and **pancreatic** tissues in **pancreatic** cancer. |
| 804 | 19880966 | The angiotensin-I-converting enzyme inhibitor enalapril and aspirin delay progression of **pancreatic** intraepithelial neoplasia and cancer formation in a genetically engineered mouse model of **pancreatic** cancer. |
| 805 | 18981953 | The clinical and radiological characteristics of focal mass-forming autoimmune **pancreatitis**: comparison with chronic **pancreatitis** and **pancreatic** cancer. |
| 806 | 19175829 | Expression of intercellular adhesion molecule (ICAM)-1 or ICAM-2 is critical in determining sensitivity of **pancreatic** cancer cells to cytolysis by human gammadelta-T cells: implications in the design of gammadelta-T-cell-based immunotherapies for **pancreatic** cancer. |
| 807 | 17220612 | 4. Chronic **pancreatitis** and **pancreatic** cancer, lifestyle-related diseases. |
| 808 | 15942269 | History of the European **pancreatic** club: the first 40 years 1965-2005. The scientific profile of the European **pancreatic** club and what stood the test of time. |
| 809 | 24476826 | Follicular **pancreatitis**, report of a case clinically mimicking **pancreatic** cancer and literature review. |
| 810 | 25288201 | Differentiating autoimmune **pancreatitis** from **pancreatic** cancer. |
| 811 | 12832972 | Postoperative cytology for drained fluid from the **pancreatic** bed after "curative" resection of **pancreatic** cancers: does it predict both the patient's prognosis and the site of cancer recurrence? |
| 812 | 8479461 | **Pancreatitis** and the risk of **pancreatic** cancer. International **Pancreatitis** Study Group. |
| 813 | 23891972 | Activated **pancreatic** stellate cells sequester CD8+ T cells to reduce their infiltration of the juxtatumoral compartment of **pancreatic** ductal adenocarcinoma. |
| 814 | 23170143 | Role of janus kinasesignal transducers and activators of transcription in the pathogenesis of **pancreatitis** and **pancreatic** cancer. |
| 815 | 12870734 | **Pancreatic** cancer after surgery for chronic **pancreatitis**. |
| 816 | 20510834 | **Pancreatic** cancer in chronic **pancreatitis**; aetiology, incidence, and early detection. |
| 817 | 23622135 | The epidemiology of **pancreatitis** and **pancreatic** cancer. |
| 818 | 8061340 | Serum soluble interleukin-2 receptor in **pancreatic** cancer and chronic **pancreatitis**. |
| 819 | 9815650 | Telomerase activity in **pancreatic** juice differentiates ductal carcinoma from adenoma and **pancreatitis**. |
| 820 | 9700949 | Role of fibroblast growth factors and their receptors in **pancreatic** cancer and chronic **pancreatitis**. |
| 821 | 8776168 | [**Pancreatic** carcinoma in chronic **pancreatitis** with inflammatory tumor of the head of the pancreas]. |
| 822 | 10547195 | Clonal preservation of human **pancreatic** cell line derived from primary **pancreatic** adenocarcinoma. |
| 823 | 10206479 | Increased angiogenin expression in obstructive chronic **pancreatitis** surrounding **pancreatic** cancer but not in pure chronic **pancreatitis**. |
| 824 | 17957501 | Excess premature (3-month) mortality in advanced **pancreatic** cancer could be related to fatal vascular thromboembolic events. A hypothesis based on a systematic review of phase III chemotherapy studies in advanced **pancreatic** cancer. |
| 825 | 2931922 | [Chronic **pancreatitis** as a predisposing factor in the development of **pancreatic** cancer. Histological and histochemical studies]. |
| 826 | 9324130 | Absence of K-ras mutations in the **pancreatic** parenchyma of patients with chronic **pancreatitis**. |
| 827 | 12918127 | p53 protein expression and CA19.9 values in differential cytological diagnosis of **pancreatic** cancer complicated with chronic **pancreatitis** and chronic **pancreatitis**. |
| 828 | 7671233 | Microsatellite instability and K-ras mutations associated with **pancreatic** adenocarcinoma and **pancreatitis**. |
| 829 | 12757156 | Overexpression of **pancreatitis**-associated protein (PAP) in human **pancreatic** ductal adenocarcinoma. |
| 830 | 7187280 | [Computer tomographic diagnosis of **pancreatitis** and **pancreatic** cancer]. |
| 831 | 8108373 | Risk of **pancreatic** carcinoma in tropical calcifying **pancreatitis**: an epidemiologic study. |
| 832 | 22196670 | Endoscopic versus surgical treatment of downstream **pancreatic** duct stones in chronic **pancreatitis**. |
| 833 | 23781378 | A case of **pancreatic** cancer in the setting of autoimmune **pancreatitis** with nondiagnostic serum markers. |
| 834 | 16533471 | Techniques and results of neurolysis for chronic **pancreatitis** and **pancreatic** cancer pain. |
| 835 | 24936576 | [Autoimmune **pancreatitis** in a patient with ulcerative colitis simulating a **pancreatic** tumor]. |
| 836 | 21054452 | Review article: enzyme supplementation in cystic fibrosis, chronic **pancreatitis**, **pancreatic** and periampullary cancer. |
| 837 | 22228050 | Diagnostic Strategy for Differentiating Autoimmune **Pancreatitis** From **Pancreatic** Cancer: Is an Endoscopic Retrograde Pancreatography Essential? |
| 838 | 19137428 | EUS-guided celiac plexus neurolysis for pain due to chronic **pancreatitis** or **pancreatic** cancer pain: a meta-analysis and systematic review. |
| 839 | 18563497 | Obesity, **pancreatitis**, and **pancreatic** cancer. |
| 840 | 20720447 | Helicobacter pylori in autoimmune **pancreatitis** and **pancreatic** carcinoma. |
| 841 | 19169171 | Amino acid malnutrition in patients with chronic **pancreatitis** and **pancreatic** carcinoma. |
| 842 | 2669339 | [The differential diagnosis of chronic **pancreatitis** and **pancreatic** cancer]. |
| 843 | 443902 | Non-operative differentiation between **pancreatic** cancer and chronic **pancreatitis**. |
| 844 | 25117030 | [Focal autoimmune **pancreatitis** versus **pancreatic** cancer: value of steroid treatment in the diagnosis]. |
| 845 | 11912681 | [**Pancreatic** excisions for chronic **pancreatitis** and cancer: their rationale for "factual" surgery. Evidence-based medicine]. |
| 846 | 17523325 | Relation between chronic **pancreatitis** and **pancreatic** cancer in the light of surgical management. |
| 847 | 17710478 | Comparison of intrahepatic and **pancreatic** perfusion on fusion images using a combined SPECTCT system and assessment of efficacy of combined continuous arterial infusion and systemic chemotherapy in advanced **pancreatic** carcinoma. |
| 848 | 10582692 | Differential diagnosis of chronic **pancreatitis** and **pancreatic** cancer in brush cytology specimens. |
| 849 | 12427788 | Risk of **pancreatic** adenocarcinoma in chronic **pancreatitis**. |
| 850 | 16553193 | [Thoracoscopic splanchnicectomy--a method of pain palliation in non-resectable **pancreatic** cancer and chronic **pancreatitis**]. |
| 851 | 20811916 | Autoimmune **pancreatitis** mimicking **pancreatic** cancer. |
| 852 | 15721759 | Radioiodinated phenylalanine derivatives to image **pancreatic** cancer: a comparative study with [18F]fluoro-2-deoxy-D-glucose in human **pancreatic** carcinoma xenografts and in inflammation models. |
| 853 | 1689141 | Immunohistochemical staining of **pancreatic** cancer with CA19-9, KM01, unabsorbed CEA, and absorbed CEA. A comparison with normal pancreas and chronic **pancreatitis**. |
| 854 | 3218913 | C reactive protein in **pancreatic** cancer and chronic **pancreatitis**. |
| 855 | 15672060 | Differential diagnosis of **pancreatic** cancer and focal **pancreatitis** by using EUS-guided FNA. |
| 856 | 24293974 | Nutritional status and nutritional support before and after pancreatectomy for **pancreatic** cancer and chronic **pancreatitis**. |
| 857 | 23057427 | [Differential diagnosis of the chronic **pancreatitis** and the **pancreatic** ductal adenocarcinoma]. |
| 858 | 21947719 | Autoimmune **pancreatitis** versus **pancreatic** cancer: a comprehensive review with emphasis on differential diagnosis. |
| 859 | 23400785 | [Chronic **pancreatitis** or **pancreatic** malignancy: clinical and radiological differential diagnosis of pancreas head space-occupying mass]. |
| 860 | 21734390 | Environmental risk factors for chronic **pancreatitis** and **pancreatic** cancer. |
| 861 | 21039739 | Potential value of serum total IgE for differentiation between autoimmune **pancreatitis** and **pancreatic** cancer. |
| 862 | 19407482 | Identification of a novel kindred with familial **pancreatitis** and **pancreatic** cancer. |
| 863 | 19760029 | **Pancreatitis** and **pancreatic** cancer in two large pooled case-control studies. |
| 864 | 10448129 | **Pancreatic** cancer in patients with chronic **pancreatitis**: a challenge from a surgical perspective. |
| 865 | 12397770 | Prolidase activity disregulation in chronic **pancreatitis** and **pancreatic** cancer. |
| 866 | 17575540 | Adiponectin as a potential differential marker to distinguish **pancreatic** cancer and chronic **pancreatitis**. |
| 867 | 18815540 | Strategy for differentiating autoimmune **pancreatitis** from **pancreatic** cancer. |
| 868 | 16240234 | Determination of plasma trypsin-like activity in healthy subjects, patients with mild to moderate alcoholic chronic **pancreatitis**, and patients with nonjaundice **pancreatic** cancer. |
| 869 | 1691065 | Renal handling of amylase and immunoreactive trypsin in **pancreatic** cancer and chronic **pancreatitis**. |
| 870 | 2408788 | Immunoreactive phospholipase A2 in serum in acute **pancreatitis** and **pancreatic** cancer. |
| 871 | 7797022 | **Pancreatitis** is a risk factor for **pancreatic** cancer. |
| 872 | 16995472 | Comparative evaluation of p53 mutation in **pancreatic** adenocarcinoma and chronic **pancreatitis**. |
| 873 | 22140463 | Acute **pancreatitis** accelerates initiation and progression to **pancreatic** cancer in mice expressing oncogenic Kras in the nestin cell lineage. |
| 874 | 25785724 | Serum IgG4 Elevation in **Pancreatic** Cancer: Diagnostic and Prognostic Significance and Association With Autoimmune **Pancreatitis**. |
| 875 | 18192887 | Is **pancreatic** core biopsy sufficient to diagnose autoimmune chronic **pancreatitis**? |
| 876 | 20705257 | Relapsed acute **pancreatitis** as the initial presentation of **pancreatic** cancer in a young man: a case report. |
| 877 | 21661440 | Incidence of and risk factors for developing **pancreatic** cancer in patients with chronic **pancreatitis**. |
| 878 | 19012035 | Mechanisms of **pancreatic** fibrosis and applications to the treatment of chronic **pancreatitis**. |
| 879 | 22369017 | [CFTR F508DEL mutation and 5T allele in patients with chronic **pancreatitis** and **pancreatic** adenocarcinoma]. |
| 880 | 24152948 | **Pancreatitis**-diabetes-**pancreatic** cancer: summary of an NIDDK-NCI workshop. |
| 881 | 24440214 | Increased risk of **pancreatic** adenocarcinoma after acute **pancreatitis**. |
| 882 | 10021685 | **Pancreatic** cancer. |
| 883 | 12181237 | Advanced **pancreatic** cancer--5 years on. |
| 884 | 12185230 | Eosinophilic gastroenteritis mimicking **pancreatic** cancer. |
| 885 | 10026856 | Inhibition of human **pancreatic** cancer growth by the adenovirus-mediated introduction of a novel growth suppressing gene, tob, in vitro. |
| 886 | 12187833 | [Dominant negative HIF-1 alpha reduces tumorigenicity of a human **pancreatic** cancer cell line]. |
| 887 | 10030405 | Chemoradiation for localized **pancreatic** cancer: another perspective. |
| 888 | 10047695 | [A case of branch duct type mucin producing **pancreatic** cancer in which endoscopic ultrasonography was useful in determining operative procedure]. |
| 889 | 10051796 | [Treatment of **pancreatic** cancer]. |
| 890 | 10075463 | Effect of flutamide on survival in patients with **pancreatic** cancer. Results are impressive. |
| 891 | 10075464 | Effect of flutamide on survival in patients with **pancreatic** cancer. No recommendations can be made. |
| 892 | 10076778 | Risk and genetic factors in **pancreatic** cancer. |
| 893 | 10101463 | [Activation of blood coagulation in patients with **pancreatic** cancer]. |
| 894 | 1010707 | Diagnosis of **pancreatic** cancer. |
| 895 | 1019833 | Unique features of serially transplanted human **pancreatic** cancer in nude mice. |
| 896 | 1019937 | Development of an animal model of **pancreatic** cancer. |
| 897 | 15708131 | Regarding "invasive **pancreatic** cancer presenting as gastrointestinal hemorrhage-a case report". |
| 898 | 15708136 | Palliation in **pancreatic** cancer: the controversies continue. |
| 899 | 15739061 | [A rare case of isolated prostate metastasis from primary **pancreatic** cancer]. |
| 900 | 15750203 | The genetic basis of sporadic **pancreatic** cancer. |
| 901 | 12217773 | Aspirin may lower risk of **pancreatic** cancer. |
| 902 | 12297741 | EUS in **pancreatic** cancer. |
| 903 | 12297755 | Screening and surveillance for hereditary **pancreatic** cancer. |
| 904 | 12370627 | **Pancreatic** cancer: evidence-based diagnosis and treatment. |
| 905 | 1020260 | [ABO blood-group system in patients with **pancreatic** cancer]. |
| 906 | 10206478 | Workup of a patient with familial **pancreatic** cancer. |
| 907 | 10214082 | [A case of small **pancreatic** cancer with sarcoid reaction]. |
| 908 | 10218452 | The impact of different types of surgery in **pancreatic** cancer. |
| 909 | 10218453 | (Neo)adjuvant treatment in **pancreatic** cancer--the need for future trials. |
| 910 | 1022377 | The present status of carcinoembryonic antigen (CEA) in diagnosis, detection of recurrence, prognosis and evaluation of therapy of colonic and **pancreatic** cancer. |
| 911 | 12399802 | **Pancreatic** cancer. |
| 912 | 1240415 | [Pain of the lower abdomen and dryness of the mouth (with chyliform ascites and glycosuria): **pancreatic** cancer (with chyliform ascites)]. |
| 913 | 12404258 | Discussion on 3-hydroxy-3-methylglutaryl-coenzyme a reductase inhibitors reduce human **pancreatic** cancer cell invasion and metastasis. |
| 914 | 12410435 | [**Pancreatic** cancer--Case report]. |
| 915 | 12410436 | [**Pancreatic** cancer--diagnostic]. |
| 916 | 12410437 | [**Pancreatic** cancer--treatment]. |
| 917 | 12418474 | Symposium on Gastrointestinal, Liver and **Pancreatic** Cancer. Venice, Italy, 6-8 June 2002. |
| 918 | 12425453 | Multidetector row CT with dual-phase CT angiography in the preoperative evaluation of **pancreatic** cancer. |
| 919 | 12432282 | Inactivation of tumor suppressor genes by promoter methylation is important in the pathogenesis of **pancreatic** cancer. |
| 920 | 12441320 | Chemoradiation protocols offer only incremental gains for **pancreatic** cancer patients. |
| 921 | 12441321 | Stat bite: **Pancreatic** cancer incidence in U.S. blacks and whites, 1973-1999. |
| 922 | 12451033 | Diagnostic clues for early **pancreatic** cancer. |
| 923 | 10228756 | Early detection and high risk of **pancreatic** cancer: an introduction. |
| 924 | 10228757 | Early diagnosis of **pancreatic** cancer. |
| 925 | 10228851 | A case of aberrant **pancreatic** cancer in the jejunum. |
| 926 | 10232158 | Treatment for **pancreatic** cancer. |
| 927 | 10232159 | Symptom management for patients with **pancreatic** cancer. |
| 928 | 10235084 | [Conclusions of the First Workshop of the oncology societies AIOM, AIRO and SICO about **pancreatic** cancer]. |
| 929 | 10323871 | Towards immunotherapy of **pancreatic** cancer. |
| 930 | 10336818 | Isolated hypoxic perfusion with mitomycin C in patients with advanced **pancreatic** cancer. |
| 931 | 10356699 | A 38-year-old man with **pancreatic** cancer. |
| 932 | 15782110 | The first initiative targeted to increase the training of African-American scientists in **pancreatic** cancer research: the Mayo Clinic College of Medicine-Oakwood College alliance. |
| 933 | 15809471 | Computed tomography versus endoscopic ultrasonography for staging of **pancreatic** cancer. |
| 934 | 15827787 | Does size matter most? Reassessing clinical staging for **pancreatic** cancer. |
| 935 | 10382724 | Platelet counts and prognosis of **pancreatic** cancer. |
| 936 | 12459728 | **Pancreatic** cancer biology and genetics. |
| 937 | 12469795 | **Pancreatic** cancer--a major health problem requiring centralization and multi-disciplinary team-work for improved results. |
| 938 | 12476120 | Intraocular metastasis of **pancreatic** cancer: report of two cases. |
| 939 | 15830299 | Dietary intake as a risk factor for **pancreatic** cancer in Japan: high cholesterol and low vitamin C diet. |
| 940 | 10388102 | **Pancreatic** Cancer: Local Success and Distant Failure. |
| 941 | 10401749 | **Pancreatic** cancer: "true, false, or just a start?". |
| 942 | 10409101 | [Surgical treatment of **pancreatic** cancer--balancing the radicality with the quality of life]. |
| 943 | 10413417 | Stat bite: U.S. **pancreatic** cancer survival rates by age. |
| 944 | 10415858 | p22PRG1: a novel early response gene in **pancreatic** cancer cells regulated by p53 and NF kappa B. |
| 945 | 10415869 | CD44, bFGF and hyaluronan in human **pancreatic** cancer cell lines. |
| 946 | 10415876 | Genetic prodrug activation therapy for **pancreatic** cancer. |
| 947 | 10419716 | Intraoperative celiac plexus block in the surgical palliation for unresectable **pancreatic** cancer. |
| 948 | 1587372 | Probable **pancreatic** cancer in a pre-eclamptic patient. |
| 949 | 15882933 | Adding irinotecan to first-line gemcitabine improves tumour response in advanced **pancreatic** cancer. |
| 950 | 15887154 | **Pancreatic** cancer: basic and clinical aspects. |
| 951 | 15888741 | ESMO Minimum Clinical Recommendations for diagnosis, treatment and follow-up of **pancreatic** cancer. |
| 952 | 15888770 | Guidelines for the management of patients with **pancreatic** cancer periampullary and ampullary carcinomas. |
| 953 | 12517564 | Preoperative imaging of **pancreatic** cancer: a management-oriented approach. |
| 954 | 12531614 | **Pancreatic** cancer and basal-cell carcinoma. |
| 955 | 15905855 | Immunotherapy for **pancreatic** cancer - science driving clinical progress. |
| 956 | 10436939 | [Status and prospects of **pancreatic** cancer diagnosis in China]. |
| 957 | 12556242 | The relationship between diabetes and **pancreatic** cancer. |
| 958 | 12556803 | Acute portal vein thrombosis after EUS-guided FNA of **pancreatic** cancer: case report. |
| 959 | 12556976 | Reply: Gallstones, cholecystectomy, and the risk for developing **pancreatic** cancer. |
| 960 | 10442915 | Epidemiology of **pancreatic** cancer and diet in Australia. |
| 961 | 12569133 | Familial **pancreatic** cancer: where are we in 2003? |
| 962 | 10449812 | Assessment and treatment of the patient with **pancreatic** cancer: introduction. |
| 963 | 15941975 | Lack of association of physical activity and obesity with incident **pancreatic** cancer in elderly women. |
| 964 | 12601933 | [Gemcitabine treatment of **pancreatic** cancer peritonitis]. |
| 965 | 12619625 | Reducing your risk of **pancreatic** cancer. |
| 966 | 12622409 | Emerging cellular and molecular themes in **pancreatic** cancer research. |
| 967 | 15941976 | No association between dietary glycemic index or load and **pancreatic** cancer incidence in postmenopausal women. |
| 968 | 15944164 | Comparison of **pancreatic** cancer mortality in five countries: France, Italy, Japan, UK and USA from WHO mortality database (1960-2000). |
| 969 | 15948320 | Combination chemotherapy for advanced **pancreatic** cancer--has its time finally come? |
| 970 | 15959788 | Osteonecrosis induced by intraoperative radiotherapy for **pancreatic** cancer. |
| 971 | 10452696 | Hepatocyte growth factor and **pancreatic** cancer cells. |
| 972 | 10462670 | Hemobilia in advanced **pancreatic** cancer with portal vein obstruction and a metal endobiliary stent: a case report. |
| 973 | 10470520 | The continuing challenge of exocrine **pancreatic** cancer. |
| 974 | 10473729 | **Pancreatic** cancer in a young adult after treatment for Hodgkin's disease. |
| 975 | 10481775 | [Multimodality therapy of operable **pancreatic** cancer]. |
| 976 | 12642681 | An introduction to **pancreatic** adenocarcinoma genetics, pathology and therapy. |
| 977 | 15987923 | Case records of the Massachusetts General Hospital. Case 20-2005. A 58-year-old man with locally advanced **pancreatic** cancer. |
| 978 | 16002845 | Locally advanced **pancreatic** cancer. |
| 979 | 10491423 | **Pancreatic** cancer research: putting all their ducts in a row. |
| 980 | 10503148 | Endoscopic ultrasound in **pancreatic** diseases. Indications, limitations, and the future. |
| 981 | 10527614 | Surgical palliation of **pancreatic** cancer. |
| 982 | 16041106 | Mixed exocrine-endocrine **pancreatic** carcinoma in childhood. |
| 983 | 1606410 | [Early diagnosis of **pancreatic** cancer--is it senseless?]. |
| 984 | 1606411 | [Staging of **pancreatic** cancer: why and how?]. |
| 985 | 12658505 | Value of 18-F-deoxyglucose (FDG) positron emission tomography (PET) for the diagnosis of **pancreatic** cancer. |
| 986 | 12659113 | Physical activity, body weight, and **pancreatic** cancer mortality. |
| 987 | 12673125 | In search of an early warning system for **pancreatic** cancer. |
| 988 | 12673738 | Familial **pancreatic** cancer. |
| 989 | 12680287 | [Extended surgery in locally invasive **pancreatic** cancer. An original revascularization technique]. |
| 990 | 12683400 | Environmental factors and risk of **pancreatic** cancer. |
| 991 | 12683401 | Unfriendly chemicals in **pancreatic** cancer. |
| 992 | 12687975 | [Complementary tests and **pancreatic** cancer]. |
| 993 | 10612968 | Effect of curative versus palliative resection for stage III **pancreatic** cancer patients. 1999; 44(4): 231-5. |
| 994 | 10616684 | **Pancreatic** cancer: any prospects for prevention? |
| 995 | 10637261 | Docetaxel chemotherapy for **pancreatic** cancer: Do results support certainty? Italian Group for the Study of Gastrointestinal Tract Carcinomas. |
| 996 | 10638041 | [Epidemiology of **pancreatic** cancer]. |
| 997 | 10638042 | [Molecular genetics of human **pancreatic** cancer]. |
| 998 | 10638044 | [The laboratory in the diagnosis of **pancreatic** cancer]. |
| 999 | 10638047 | [Adjuvant and palliative treatment of **pancreatic** cancer]. |
