# Supplementary material for: PubMedPortable: A Framework for Supporting the Development of Text Mining Applications
Source: PLoS One. 2016 Oct 5;11(10):e0163794. doi: 10.1371/journal.pone.0163794 (PMC5051953; doi:10.1371/journal.pone.0163794)
Supplement: S1 File — (ZIP) [file pone.0163794.s001.zip › PubMedPortable-master/full_text_index/Xapian_query_results_NEAR.html]

Xapian\_query\_results\_NEAR


| Rank | PubMed-ID | Title (query term highlighted) |
| --- | --- | --- |
| 0 | 16317266 | **Erlotinib** and chemoradiation followed by maintenance **erlotinib** for locally advanced **pancreatic** **cancer**: a phase I study. |
| 1 | 17878488 | **Erlotinib** in **pancreatic** **cancer** patients: do we need more information from the NCIC CTG trial? |
| 2 | 17906218 | Does a statistically significant survival benefit of **erlotinib** plus gemcitabine for advanced **pancreatic** **cancer** translate into clinical significance and value? |
| 3 | 18089885 | **Erlotinib** in **pancreatic** **cancer**: are tumor cells the (only) target? |
| 4 | 18615326 | Irreversible ototoxicity associated with the use of **erlotinib** in a patient with **pancreatic** **cancer**. |
| 5 | 18360654 | Role of **erlotinib** in the management of **pancreatic** **cancer**. |
| 6 | 17704662 | Severe lung and skin toxicity during treatment with gemcitabine and **erlotinib** for metastatic **pancreatic** **cancer**. |
| 7 | 20868602 | [Efficacy of gemcitabine combined with **erlotinib** in patients with advanced **pancreatic** **cancer**]. |
| 8 | 17935272 | Tyrosine kinase inhibitors in non-small cell lung and **pancreatic** **cancer**: the emerging role of **erlotinib**. |
| 9 | 23378339 | **Erlotinib** prolongs survival in **pancreatic** **cancer** by blocking gemcitabine-induced MAPK signals. |
| 10 | 20107864 | A phase II study of isoflavones, **erlotinib**, and gemcitabine in advanced **pancreatic** **cancer**. |
| 11 | 22272248 | Phase I evaluation of intravenous ascorbic acid in combination with gemcitabine and **erlotinib** in patients with metastatic **pancreatic** **cancer**. |
| 12 | 20927525 | Phase II study of a fixed dose-rate infusion of gemcitabine associated with **erlotinib** in advanced **pancreatic** **cancer**. |
| 13 | 23435671 | KRAS mutation status is not predictive for objective response to anti-EGFR treatment with **erlotinib** in patients with advanced **pancreatic** **cancer**. |
| 14 | 24413779 | Management of advanced **pancreatic** **cancer** with gemcitabine plus **erlotinib**: efficacy and safety results in clinical practice. |
| 15 | 25145842 | Response to GEMOX plus **erlotinib** in **pancreatic** **cancer** is associated with ERCC1 overexpression. |
| 16 | 23235319 | Phase I trial of gemcitabine combined with capecitabine and **erlotinib** in advanced **pancreatic** **cancer**: a clinical and pharmacological study. |
| 17 | 17947726 | Capecitabine plus **erlotinib** in gemcitabine-refractory advanced **pancreatic** **cancer**. |
| 18 | 25247318 | Dose escalation to rash for **erlotinib** plus gemcitabine for metastatic **pancreatic** **cancer**: the phase II RACHEL study. |
| 19 | 25864651 | Amiloride sensitizes human **pancreatic** **cancer** cells to **erlotinib** in vitro through inhibition of the PI3KAKT signaling pathway. |
| 20 | 24857345 | A phase II study of **erlotinib** in gemcitabine refractory advanced **pancreatic** **cancer**. |
| 21 | 24574334 | Phase II trial of sorafenib and **erlotinib** in advanced **pancreatic** **cancer**. |
| 22 | 20130876 | A phase II study of bevacizumab plus **erlotinib** for gemcitabine-refractory metastatic **pancreatic** **cancer**. |
| 23 | 19307500 | Phase III trial of bevacizumab in combination with gemcitabine and **erlotinib** in patients with metastatic **pancreatic** **cancer**. |
| 24 | 22614154 | Comparison of the efficacy and the toxicity between gemcitabine with capecitabine (GC) and gemcitabine with **erlotinib** (GE) in unresectable **pancreatic** **cancer**. |
| 25 | 22699203 | The association of rash severity with overall survival: findings from patients receiving **erlotinib** for **pancreatic** **cancer** in the community setting. |
| 26 | 25688740 | Assessing the benefit-risk of new treatments using generalised pairwise comparisons: the case of **erlotinib** in **pancreatic** **cancer**. |
| 27 | 21549514 | Phase I study of conformal radiotherapy and concurrent full-dose gemcitabine with **erlotinib** for unresected **pancreatic** **cancer**. |
| 28 | 17457047 | ErbB3 expression and dimerization with EGFR influence **pancreatic** **cancer** cell sensitivity to **erlotinib**. |
| 29 | 25164437 | pERK, pAKT and p53 as tissue biomarkers in **erlotinib**-treated patients with advanced **pancreatic** **cancer**: a translational subgroup analysis from AIO-PK0104. |
| 30 | 17079479 | Potentiation of the effect of **erlotinib** by genistein in **pancreatic** **cancer**: the role of Akt and nuclear factor-kappaB. |
| 31 | 18803351 | Anti-tumor activity of **erlotinib** in the BxPC-3 **pancreatic** **cancer** cell line. |
| 32 | 23472089 | Gemcitabine plus **erlotinib** for advanced **pancreatic** **cancer**: a systematic review with meta-analysis. |
| 33 | 23169292 | EGFR pathway biomarkers in **erlotinib**-treated patients with advanced **pancreatic** **cancer**: translational results from the randomised, crossover phase 3 trial AIO-PK0104. |
| 34 | 23180942 | Insights into **erlotinib** action in **pancreatic** **cancer** cells using a combined experimental and mathematical approach. |
| 35 | 25503169 | Radiation recall gastritis secondary to **erlotinib** in a patient with **pancreatic** **cancer**. |
| 36 | 22593509 | Combination therapy with gemcitabine (GEM) and **erlotinib** (E) in exocrine **pancreatic** **cancer** under special reference to RASH and the tumour marker CA19-9. |
| 37 | 19858399 | Dose finding and early efficacy study of gemcitabine plus capecitabine in combination with bevacizumab plus **erlotinib** in advanced **pancreatic** **cancer**. |
