# Supplementary material for: PubMedPortable: A Framework for Supporting the Development of Text Mining Applications
Source: PLoS One. 2016 Oct 5;11(10):e0163794. doi: 10.1371/journal.pone.0163794 (PMC5051953; doi:10.1371/journal.pone.0163794)
Supplement: S1 File — (ZIP) [file pone.0163794.s001.zip › PubMedPortable-master/full_text_index/Xapian_query_results_OR.html]

Xapian\_query\_results OR


| Rank | PubMed-ID | Title (query term highlighted) | Abstract (query term highlighted) |
| --- | --- | --- | --- |
| 0 | 22977306 | Weekly paclitaxel, gemcitabine, and external irradiation followed by randomized farnesyl transferase inhibitor **R115777** for locally advanced pancreatic cancer. | PURPOSE: The Radiation Therapy Oncology Group (RTOG) multi-institutional Phase II study 98-12, evaluating paclitaxel and concurrent radiation (RT) for locally advanced pancreatic cancer, demonstrated a median survival of 11.3 months and a 1-year survival of 43%. The purpose of the randomized Phase II study by RTOG 0020 was to evaluate the addition of weekly low- dose gemcitabine with concurrent paclitaxelRT and to evaluate the efficacy and safety of the farnesyl transferase inhibitor **R115777** following chemoradiation. PATIENTS AND METHODS: Patients with unresectable, nonmetastatic adenocarcinoma of the pancreas were eligible. Patients in Arm 1 received gemcitabine, 75 mgm(2)week, and paclitaxel, 40 mgm(2)week, for 6 weeks, with 50.4 Gy radiation (CXRT). Patients in Arm 2 received an identical chemoradiation regimen but then received maintenance **R115777**, 300 mg twice a day for 21 days every 28 days (CXRT+**R115777**), until disease progression or unacceptable toxicity. RESULTS: One hundred ninety-five patients were entered into this study, and 184 were analyzable. Grade 4 nonhematologic toxicities occurred in less than 5% of CXRT patients. The most common grade 34 toxicity from **R115777** was myelosuppression; however, grade 34 hepatic, metabolic, musculoskeletal, and neurologic toxicities were also reported. The median survival time was 11.5 months and 8.9 months for the CXRT and CXRT+**R115777** arms, respectively. CONCLUSIONS: The CXRT arm achieved a median survival of almost 1-year, supporting chemoradiation as an important therapeutic modality for locally advanced pancreatic cancer. Maintenance **R115777** is not effective and is associated with a broad range of toxicities. These findings provide clinical evidence that inhibition of farnesylation affects many metabolic pathways, underscoring the challenge of developing an effective K-ras inhibitor. |
| 1 | 15805288 | Farnesyl transferase inhibitor (**R115777**)-induced inhibition of STAT3(Tyr705) phosphorylation in human pancreatic cancer cell lines require extracellular signal-regulated kinases. | In this study, we report that **R115777**, a nonpeptidomimetic farnesyl transferase inhibitor, suppresses the growth of human pancreatic adenocarcinoma cell lines and that this growth inhibition is associated with modulation in the phosphorylation levels of signal transducers and activators of transcription 3 (STAT3) and extracellular signal-regulated kinases (ERK). Treatment of cells with **R115777** inhibited the tyrosine phosphorylation of STAT3((Tyr705)), while increasing the serine phosphorylation of STAT3((Ser727)). We found the differential phosphorylation of STAT3 was due to an increased and prolonged activation of ERKs. The biological significance of ERK-mediated inhibition of STAT3((Tyr705)) phosphorylation was further assessed by treating the cells with an inhibitor (PD98059) of mitogen-activated protein kinase kinase (MEK) or by transfecting the cells with a vector that expresses constitutively active MEK-1. Expression of constitutively active MEK-1 caused an increase of ERK activity and inhibited STAT3((Tyr705)) phosphorylation. Conversely, inhibition of ERK activity by PD98059 reversed the **R115777**-induced inhibition of STAT3((Tyr705)) phosphorylation. **R115777** also caused the inhibition of the binding of STAT3 to its consensus binding element. An increase in the activation of ERKs either by overexpressing MEK-1 or treatment of cells with **R115777** caused an up-regulation in the levels of a cyclin-dependent kinase (cdk) inhibitor, p21(cip1waf1). These observations suggest that **R115777**-induced growth inhibition is partly due to the prolonged activation of ERKs that mediates an inhibition of STAT3((Tyr705)) phosphorylation and an increase in the levels of p21(cip1waf1) in human pancreatic adenocarcinoma cell lines. |
| 2 | 12663718 | Phase II and pharmacodynamic study of the farnesyltransferase inhibitor **R115777** as initial therapy in patients with metastatic pancreatic adenocarcinoma. | PURPOSE: **R115777** is a selective nonpeptidomimetic inhibitor of farnesyltransferase (FTase), one of several enzymes responsible for posttranslational modification that is required for the function of p21(ras) and other proteins. Given that RAS mutations are nearly universal in pancreatic cancer and **R115777** demonstrated preclinical activity against pancreatic cell lines and xenografts, this phase II study was undertaken to determine its clinical activity and effect on target proteins in patients with measurable metastatic pancreatic adenocarcinoma. PATIENTS AND METHODS: Twenty patients who had not received prior therapy for metastatic disease were treated with 300 mg of **R115777** orally every 12 hours for 21 of 28 days. Inhibition of FTase activity in peripheral-blood mononuclear cells was measured using a lamin B C-terminus peptide as substrate. Western blot analysis was performed to monitor farnesylation status of the chaperone protein HDJ-2. RESULTS: No objective responses were seen. Median time to progression was 4.9 weeks, and median survival time was 19.7 weeks. The estimated 6-month survival rate was 25%, with no patients progression-free at 6 months. Grade 34 toxicities were liver enzyme elevation, anemia, neutropenia, thrombocytopenia, fatigue, nauseavomiting, rash, and anorexia. FTase activity (mean +- SD) decreased by 49.8% +- 9.8% 4 hours after treatment on day 1 and 36.1% +- 24.8% before treatment on day 15. HDJ-2 farnesylation (mean +- SD) decreased by 33.4% +- 19.8% on day 15. CONCLUSION: Although treatment with **R115777** resulted in partial inhibition of FTase activity in mononuclear cells, it did not exhibit single-agent antitumor activity in patients with previously untreated metastatic pancreatic cancer. |
| 3 | 16133800 | A phase II study of farnesyl transferase inhibitor **R115777** in pancreatic cancer: a Southwest oncology group (SWOG 9924) study. | Ninety per cent of pancreatic adenocarcinomas (PC) contain mutations of the K-Ras proto-oncogene resulting in constitutively activated Ras protein. A critical step in Ras activation is farnesylation of Ras protein. Farnesyl transferase inhibitors are compounds that inhibit farnesylation. We report the results of a phase II trial of **R115777**, an oral farnesyl transferase inhibitor, in patients with surgically incurable locally advanced or metastatic PC. Between 612000 and 11202001, 58 cases were accrued, 53 of whom were eligible and analyzable. Patients were required to have a performance status (PS) 0 to 1, be able to take oral medications, and to have adequate renal, hepatic, and hematologic functions. Fifty-five percent were male. Median age was 64.7 years (38.9 to 80.6), and patients had no previous systemic therapy for advanced PC. Treatment consisted of **R115777** 300 mg po bid given for 3 out of every 4 weeks. Toxicities were as follows: Grade 3 in 1953 (36%), grade 4 in 53 (173%), and grade 5 in 53 (8%). Most frequent toxicities were: anemia 3553 (66%), fatigue and malaise 3353 (62%), nausea 3153 (58%). Grade 5 toxicities included: thromboembolism 1, infection 2, other 1. Median survival was 2.6 months (mo) (95% CI 2.1-3.6), 6-mo survival is 19% (95% CI, 8-29%), median time to treatment failure was 1.4 mo (95% GI 1.1-1.6). **R115777** is ineffective as monotherapy in advanced pancreatic cancer. |
| 4 | 12783599 | Farnesyl transferase inhibitors in clinical development. | Farnesyl transferase (FT) inhibitors block the main post-translational modification of the Ras protein, thus interfering with its localisation to the inner surface of the plasma membrane and subsequent activation of downstream effectors. Although initially developed as a strategy to target Ras in cancer, FT inhibitors have subsequently been acknowledged as acting by additional and more complex mechanisms that may extend beyond Ras, involving RhoB, centromere-binding proteins and probably other farnesylated proteins. SCH66336 (lonafarnib, Sarasar( trade mark ); Schering-Plough), a tricyclic orally active FT inhibitor, was the first of these compounds to undergo clinical development. Gastrointestinal tract toxicities and fatigue have qualified as dose-limiting toxicities in all Phase III studies. Evidence of clinical activity has been reported. Lonafarnib combination studies with both gemcitabine and paclitaxel have been carried out. No unexpected toxicities were observed in these Phase I studies, while encouraging clinical activity was observed mainly in pancreatic cancer and non-small cell lung cancer. Further combination studies are ongoing. **R115777** (Zarnestra( trade mark ); Janssen Pharmaceutica) is another orally active FT competitive inhibitor in clinical development. Single-agent Phase III studies have shown that myelotoxicity and neurotoxicity are dose-limiting toxicities; intermittent schedule is probably better tolerated; antitumour activity is observed particularly in breast cancer and haematological malignancies. A number of combination studies with **R115777** have been carried out. A recently completed, large Phase III trial comparing gemcitabine plus **R115777** versus gemcitabine plus placebo in advanced pancreatic cancer has failed to demonstrate any survival benefit in the **R115777** arm. BMS-214662 is the third FT inhibitor in clinical development. It has the main advantage of being cytotoxic in nature, rather than cytostatic, and potent in vivo antitumour activity has been reported. A major drawback for BMS-214662 is its severe gastrointestinal and liver toxicities, which prevent the achievement of adequate systemic exposures following the oral route. Alternative ways of interference with the ras oncogene pathway, in particular inhibition of ras downstream effectors, are discussed and early clinical data are presented. |
| 5 | 16015551 | Docetaxel in the management of advanced pancreatic cancer. | The poor outcome of pancreatic cancer with conventional treatment options emphasizes the need for continued research. The benefits of gemcitabine in improving quality of life and survival have been established in patients with advanced pancreatic cancer. Randomized clinical trials studying the addition of a second drug to gemcitabine, either a classic cytotoxic (5-fluorouracil, cisplatin, irinotecan, pemetrexed, oxaliplatin, or exatecan) or targeted agents (ie, the farnesyl transferase inhibitor **R115777** or the metalloproteinase inhibitor marimastat) have not resulted in improvement in survival compared with gemcitabine alone. Although limited activity of docetaxel in patients with pancreatic adenocarcinoma has been reported in single-agent studies, attractive efficacy results have been documented with docetaxel in combination with other chemotherapeutic agents for the management of advanced pancreatic cancer. Phase I and II trials of docetaxel in combination with gemcitabine, irinotecan, 5-fluorouracil, or thalidomide, as well as trials of docetaxel and radiotherapy, suggest that docetaxel combinations in pancreatic cancer should be further studied in randomized trials. |
| 6 | 12826249 | Clinical research in pancreatic cancer: the Radiation Therapy Oncology Group trials. | To summarize the clinical research activities of the Radiation Therapy Oncology Group program in the treatment of patients with locally advanced, as well as resected, pancreatic cancer. Phase II and III clinical trials are underway, examining novel cytotoxic and targeted agents with irradiation (RT) for patients with locally advanced and resected pancreatic cancer.A Phase II study incorporating concurrent paclitaxel with external beam radiotherapy in the locally advanced setting has been completed and recently analyzed. This experience has served as the foundation of a Phase II study using concurrent paclitaxel and gemcitabine with RT followed by **R115777**, a farnesyltransferase inhibitor, as maintenance therapy. In the adjuvant treatment of pancreatic cancer, an Intergroup Phase III trial has compared "conventional" postoperative chemoirradiation (5-fluorouracil before, after, and during RT) and gemcitabine before and after RT (with 5-fluorouracil during RT). This study has recently closed, meeting its accrual goal. The successor study will evaluate the use of gemcitabine given concurrently with RT, as well as in a maintenance schedule. This report summarizes current and future Radiation Therapy Oncology Group clinical trials in the treatment of patients with localized pancreatic cancer. |
| 7 | 15084616 | Phase III trial of gemcitabine plus tipifarnib compared with gemcitabine plus placebo in advanced pancreatic cancer. | PURPOSE: To determine whether addition of the farnesyltransferase inhibitor tipifarnib (Zarnestra, **R115777**; Johnson and Johnson Pharmaceutical Research and Development, Beerse, Belgium) to standard gemcitabine therapy improves overall survival in advanced pancreatic cancer. PATIENTS AND METHODS: This randomized, double-blind, placebo-controlled study compared gemcitabine + tipifarnib versus gemcitabine + placebo in patients with advanced pancreatic adenocarcinoma previously untreated with systemic therapy. Tipifarnib was given at 200 mg bid orally continuously; gemcitabine was given at 1,000 mgm(2) intravenously weekly x 7 for 8 weeks, then weekly x 3 every 4 weeks. The primary end point was overall survival; secondary end points included 6-month and 1-year survival rates, progression-free survival, response rate, safety, and quality of life. RESULTS: Six hundred eighty-eight patients were enrolled. Baseline characteristics were well balanced between the two treatment arms. No statistically significant differences in survival parameters were observed. The median overall survival for the experimental arm was 193 v 182 days for the control arm (P =.75); 6-month and 1-year survival rates were 53% and 27% v 49% and 24% for the control arm, respectively; median progression-free survival was 112 v 109 days for the control arm. Ten drug-related deaths were reported for the experimental arm and seven for the control arm. Neutropenia and thrombocytopenia grade or = 3 were observed in 40% and 15% in the experimental arm versus 30% and 12% in the control arm. Incidences of nonhematologic adverse events were similar in two groups. CONCLUSION: The combination of gemcitabine and tipifarnib has an acceptable toxicity profile but does not prolong overall survival in advanced pancreatic cancer compared with single-agent gemcitabine. |
| 8 | 15892618 | Molecularly targeted therapy for gastrointestinal cancer. | Receptor and non-receptor tyrosine kinases (TKs) have emerged as clinically useful drug target molecules for treating gastrointestinal cancer. Imatinib mesilate (STI-571, Gleevec(TM)), an inhibitior of bcr-abl TK, which was primarily designed to treat chronic myeloid leukemia is also an inhibitor of c-kit receptor TK, and is currently the drug of choice for the therapy of metastatic gastrointestinal stromal tumors (GISTs), which frequently express constitutively activated forms of the c-kit-receptor. The epidermal growth factor receptor (EGFR), which is involved in cell proliferation, metastasis and angiogenesis, is another important target. The two main classes of EGFR inhibitors are the TK inhibitors and monoclonal antibodies. Gefitinib (ZD1839, Iressa(TM)) has been on trial for esophageal and colorectal cancer (CRC) and erlotinib (OSI-774, Tarceva(TM)) on trial for esophageal, colorectal, hepatocellular, and biliary carcinoma. In addition, erlotinib has been evaluated in a Phase III study for the treatment of pancreatic cancer. Cetuximab (IMC-C225, Erbitux(TM)), a monoclonal EGFR antibody, has been FDA approved for the therapy of irinotecan resistant colorectal cancer and has been tested for pancreatic cancer. Vascular endothelial growth factor (VEGF) and its receptor (VEGFR) are critical regulators of tumor angiogenesis. Bevacizumab (Avastin(TM)), a monoclonal antibody against VEGF, was efficient in two randomized clinical trials investigating the treatment of metastatic colorectal cancer. It is also currently investigated for the therapy of pancreatic cancer in combination with gemcitabine. Other promising new drugs currently under preclinical and clinical evaluation, are VEGFR2 inhibitor PTK787ZK 222584, thalidomide, farnesyl transferase inhibitor **R115777** (tipifarnib, Zarnestra(TM)), matrix metalloproteinase inhibitors, proteasome inhibitor bortezomib (Velcade(TM)), mammalian target of rapamycin (mTOR) inhibitors, cyclooxygenase-2 (COX-2) inhibitors, platelet derived growth factor receptor (PDGF-R) inhibitors, protein kinase C (PKC) inhibitors, mitogen-activated protein kinase kinase (MEK) 12 inhibitors, Rous sarcoma virus transforming oncogene (SRC) kinase inhibitors, histondeacetylase (HDAC) inhibitors, small hypoxia-inducible factor (HIF) inhibitors, aurora kinase inhibitors, hedgehog inhibitors, and TGF-beta signalling inhibitors. |
| 9 | 22549877 | Atorvastatin inhibits pancreatic carcinogenesis and increases survival in LSL-KrasG12D-LSL-Trp53R172H-Pdx1-Cre mice. | There are several studies supporting the role of HMG-CoA reductase inhibitors such as atorvastatin against carcinogenesis, in which inhibiting the generation of prenyl intermediates involved in protein prenylation plays the crucial role. Mutation of Kras gene is the most common genetic alteration in pancreatic cancer and the Ras protein requires prenylation for its membrane localization and activity. In the present study, the effectiveness of atorvastatin against pancreatic carcinogenesis and its effect on protein prenylation were determined using the LSL-KrasG12D-LSL-Trp53R172H-Pdx1-Cre mouse model (called Pankrasp53 mice). Five-week-old Pankrasp53 mice were fed either an AIN93M diet or a diet supplemented with 100 ppm atorvastatin. Kaplan-Meier survival analysis with Log-Rank test revealed a significant increase in survival in mice fed 100 ppm atorvastatin (171.9 ± 6.2 d) compared to the control mice (144.9 ± 8.4 d, P 0.05). Histologic and immunohistochemical analysis showed that atorvastatin treatment resulted in a significant reduction in tumor volume and Ki-67-labeled cell proliferation. Mechanistic studies on primary pancreatic tumors and the cultured murine pancreatic carcinoma cells revealed that atorvastatin inhibited prenylation in several key proteins, including Kras protein and its activities, and similar effect was observed in pancreatic carcinoma cells treated with farnesyltransferase inhibitor **R115777**. Microarray assay on the global gene expression profile demonstrated that a total of 132 genes were significantly modulated by atorvastatin; and Waf1p21, cyp51A1, and soluble epoxide hydrolase were crucial atorvastatin-targeted genes which involve in inflammation and carcinogenesis. This study indicates that atorvastatin has the potential to serve as a chemopreventive agent against pancreatic carcinogenesis. |
