# Supplementary figures and images for: PubMedPortable: A Framework for Supporting the Development of Text Mining Applications
Source: PLoS One. 2016 Oct 5;11(10):e0163794. doi: 10.1371/journal.pone.0163794 (PMC5051953; doi:10.1371/journal.pone.0163794)

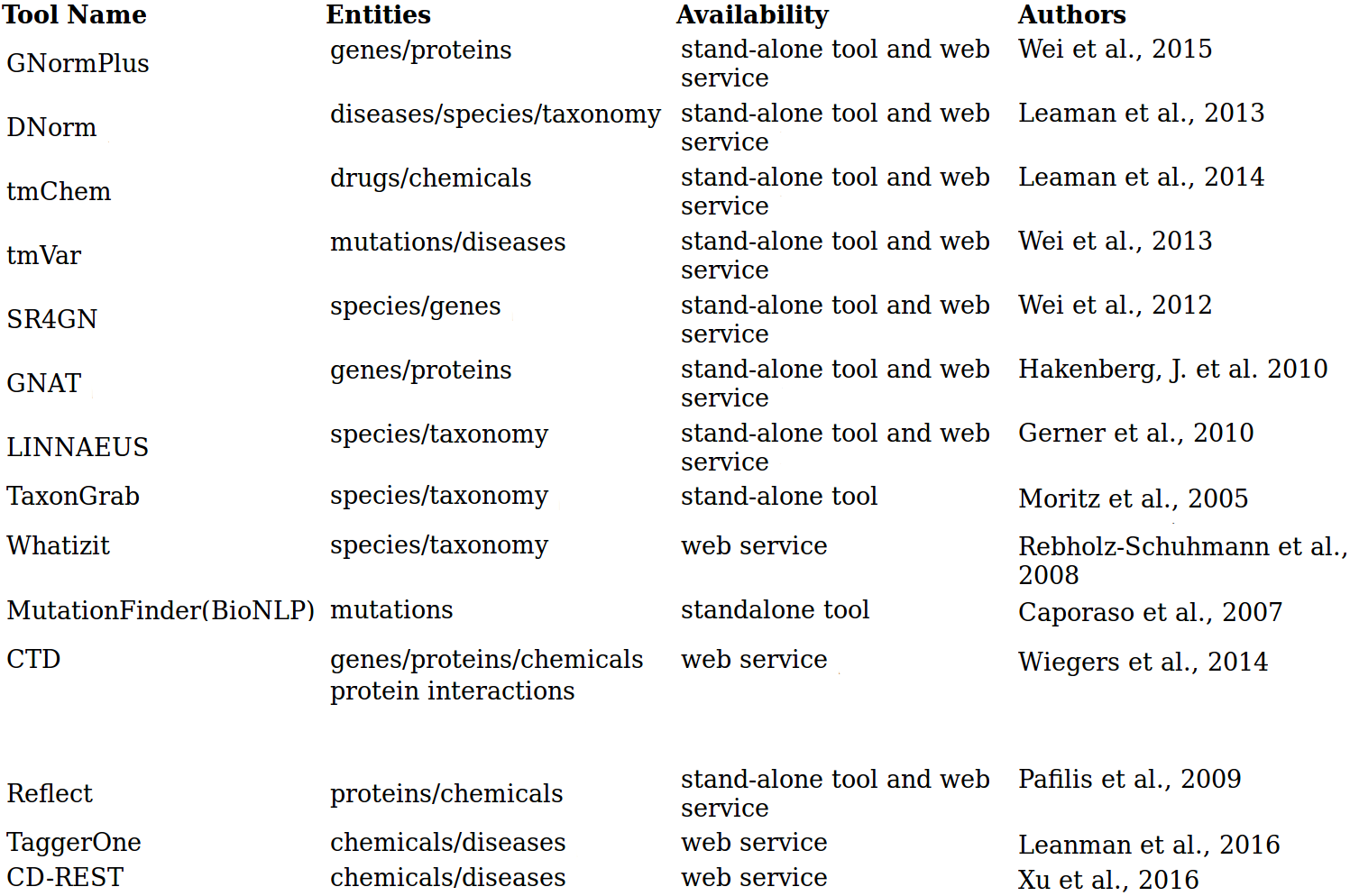

Supplement: S1 File — (ZIP) [file pone.0163794.s001.zip › PubMedPortable-master/documentation/NER_table_wiki.png]

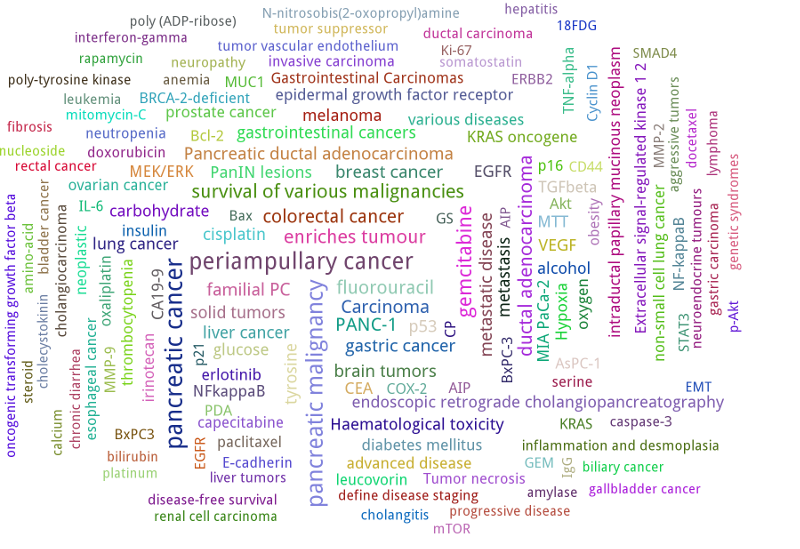

Supplement: S1 File — (ZIP) [file pone.0163794.s001.zip › PubMedPortable-master/documentation/cloud_3tools_800.png]

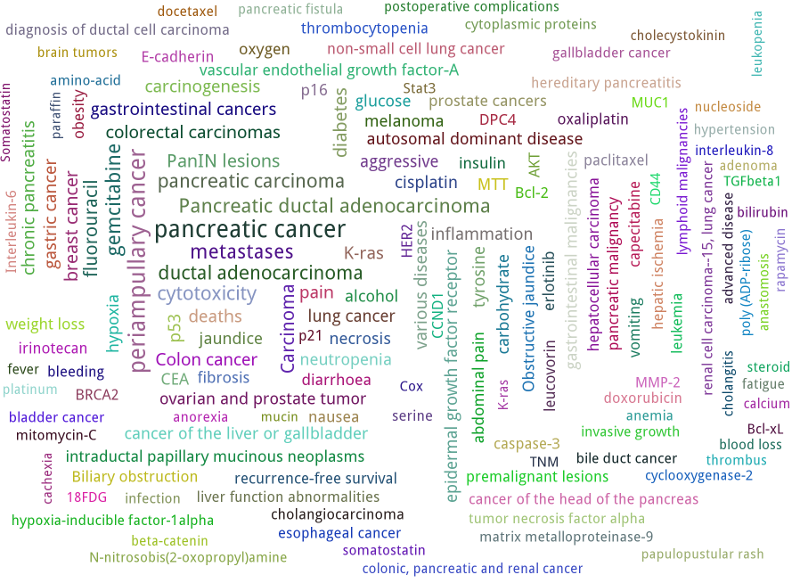

Supplement: S1 File — (ZIP) [file pone.0163794.s001.zip › PubMedPortable-master/documentation/cloud_entities_identifiers_800.png]

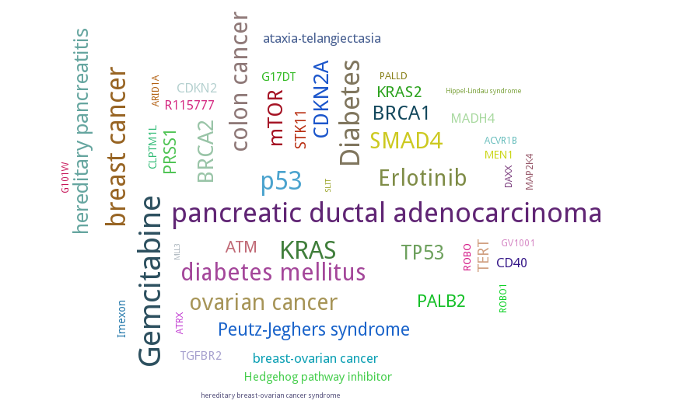

Supplement: S1 File — (ZIP) [file pone.0163794.s001.zip › PubMedPortable-master/documentation/cloud_search_terms_700_w.png]

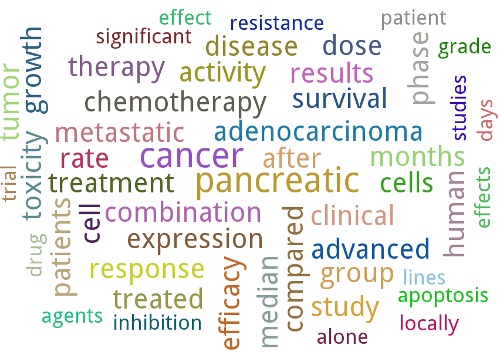

Supplement: S1 File — (ZIP) [file pone.0163794.s001.zip › PubMedPortable-master/documentation/cloud_surrounding_words_500_w.png]

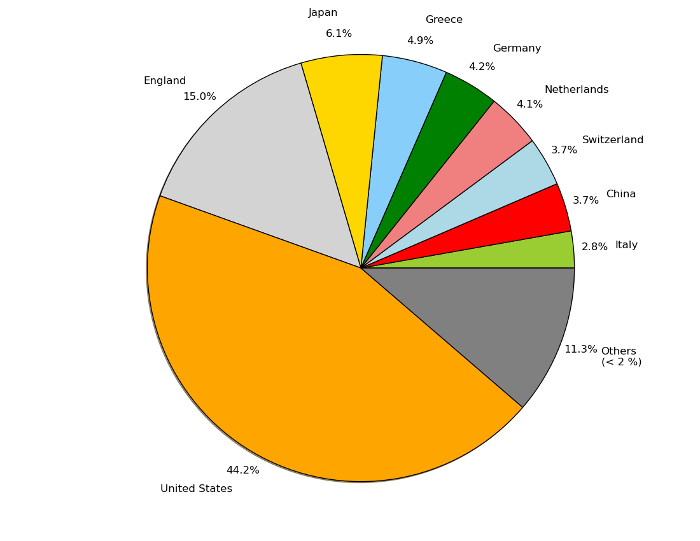

Supplement: S1 File — (ZIP) [file pone.0163794.s001.zip › PubMedPortable-master/documentation/pie_chart_countries_publications_700_w.png]

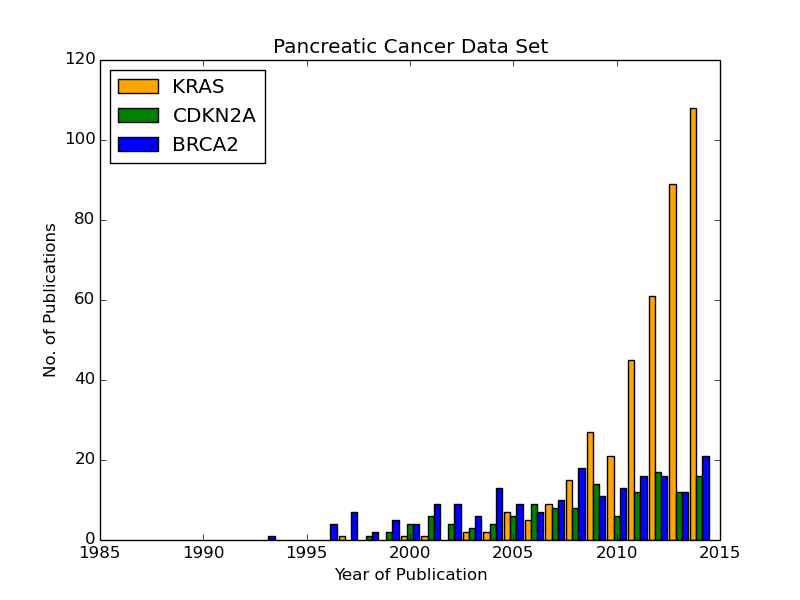

Supplement: S1 File — (ZIP) [file pone.0163794.s001.zip › PubMedPortable-master/plots/bar_chart/KRAS_BRCA2_CDKN2A.png]

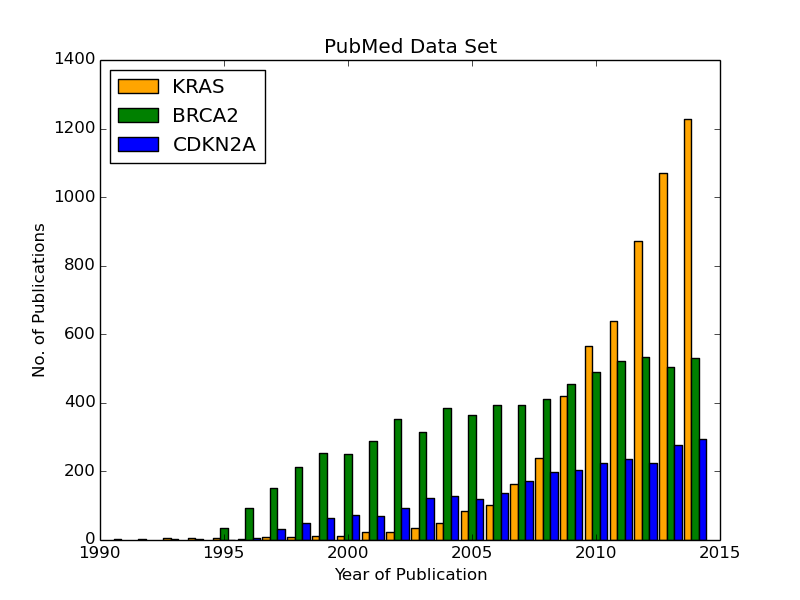

Supplement: S1 File — (ZIP) [file pone.0163794.s001.zip › PubMedPortable-master/plots/bar_chart/KRAS_BRCA2_CDKN2A_pubmed.png]

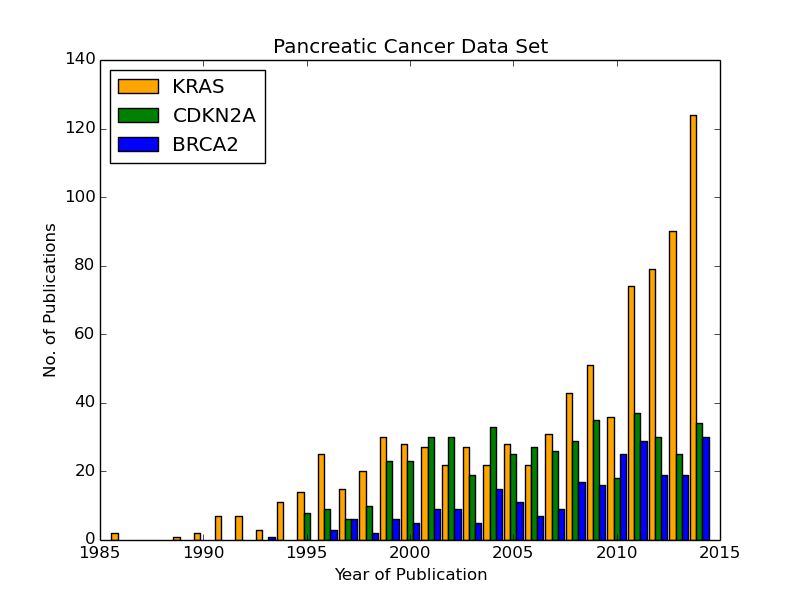

Supplement: S1 File — (ZIP) [file pone.0163794.s001.zip › PubMedPortable-master/plots/bar_chart/KRAS_CDKN2A_BRCA2.png]

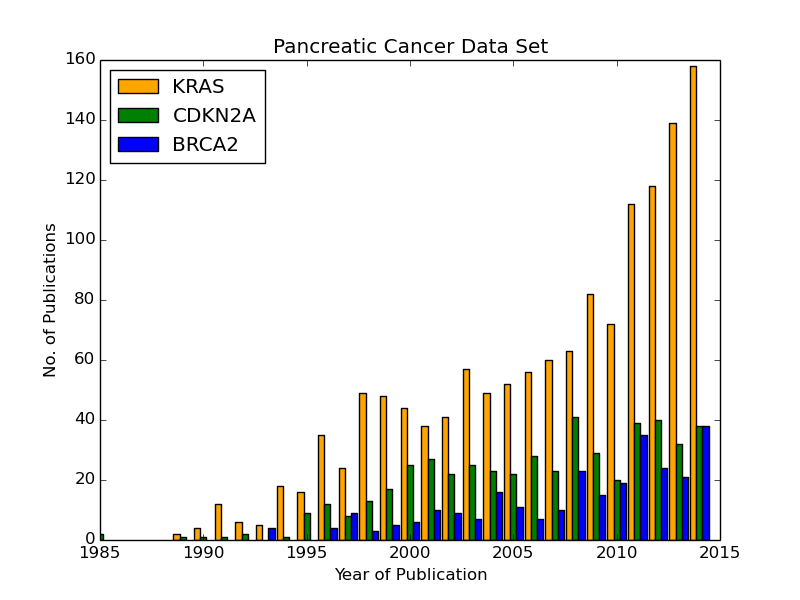

Supplement: S1 File — (ZIP) [file pone.0163794.s001.zip › PubMedPortable-master/plots/bar_chart/KRAS_CDKN2A_BRCA2_3tools.png]

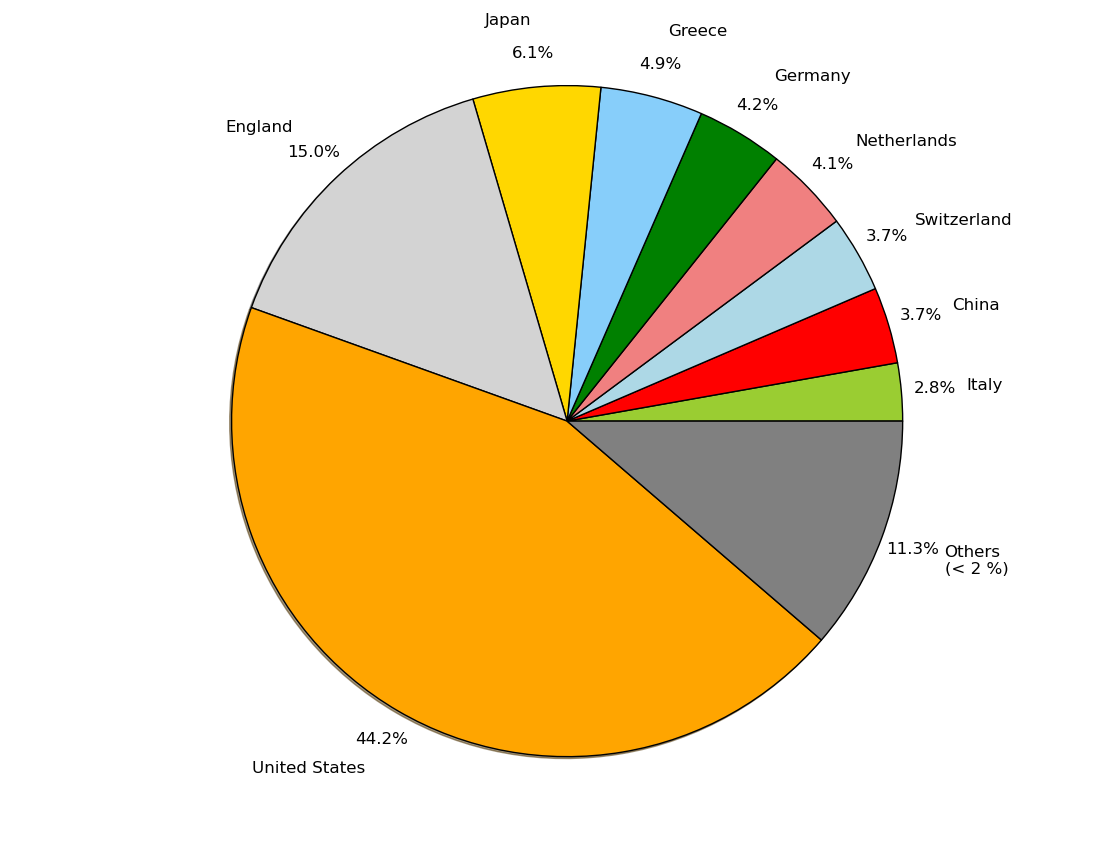

Supplement: S1 File — (ZIP) [file pone.0163794.s001.zip › PubMedPortable-master/plots/pie_chart/pie_chart_countries_publications.png]

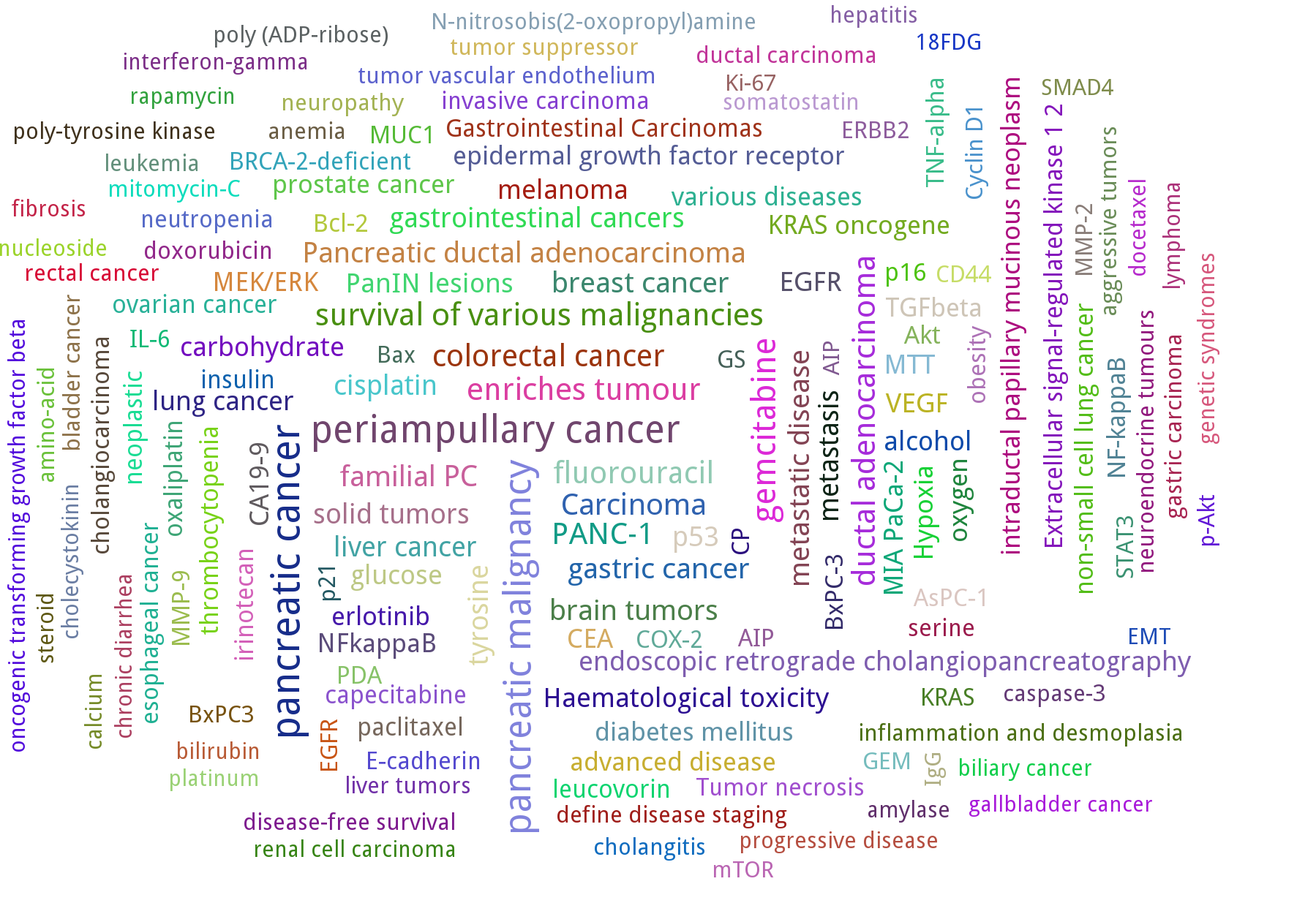

Supplement: S1 File — (ZIP) [file pone.0163794.s001.zip › PubMedPortable-master/plots/word_cloud/cloud_3tools.png]

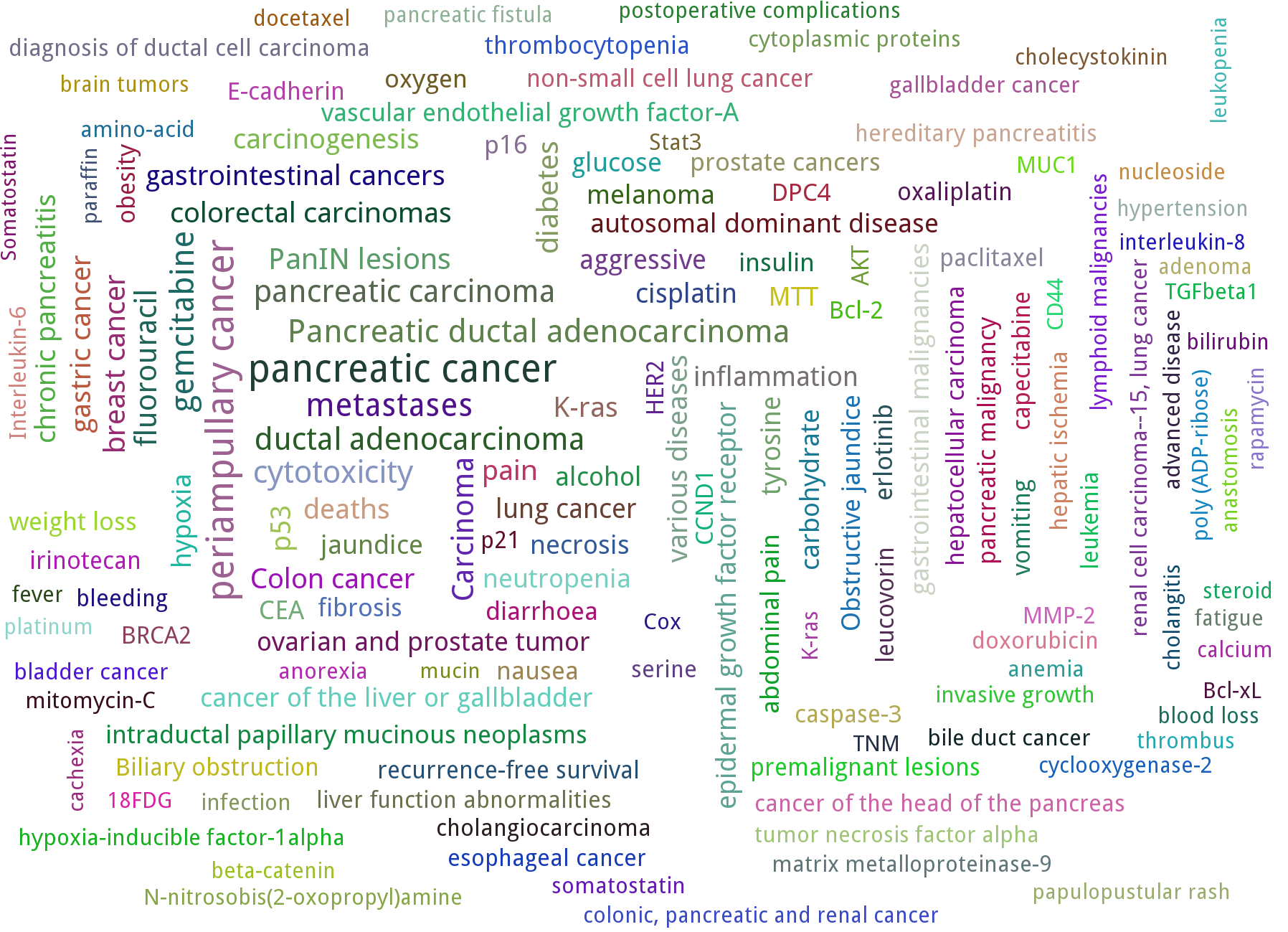

Supplement: S1 File — (ZIP) [file pone.0163794.s001.zip › PubMedPortable-master/plots/word_cloud/cloud_entities_identifiers.png]

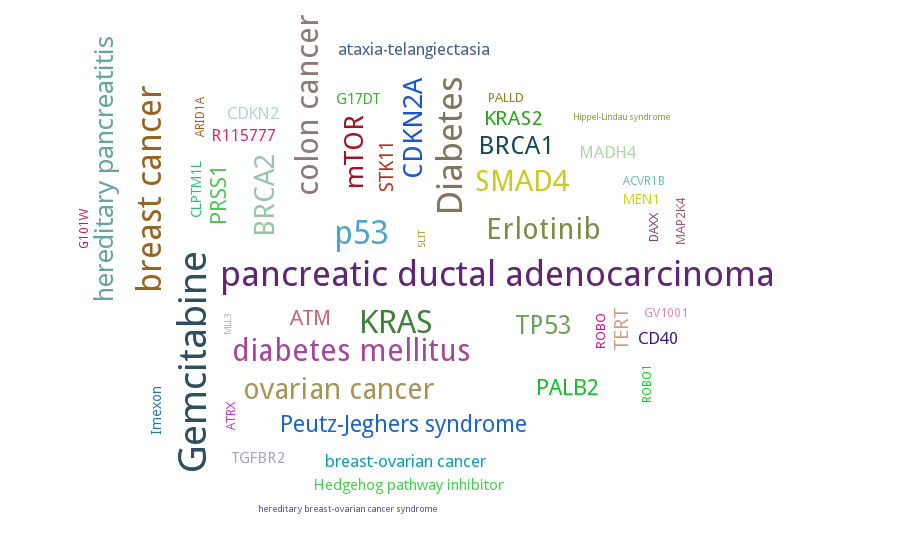

Supplement: S1 File — (ZIP) [file pone.0163794.s001.zip › PubMedPortable-master/plots/word_cloud/cloud_search_terms.png]

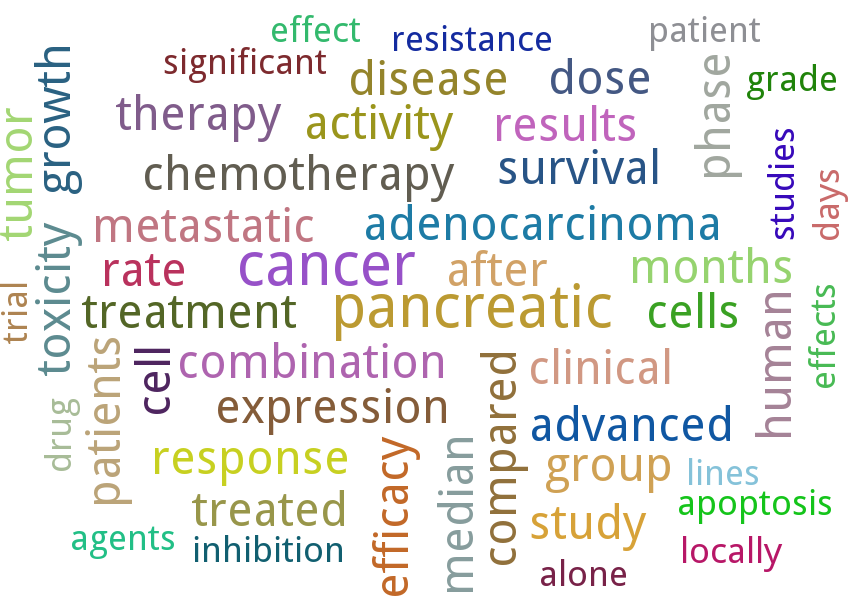

Supplement: S1 File — (ZIP) [file pone.0163794.s001.zip › PubMedPortable-master/plots/word_cloud/cloud_surrounding_words.png]
